# Comparative Analysis of the Incidence, Prevalence, and Survival of 8 Types of Parkinsonism in a Population‐Based Study with 367 Million Person Years of Observation over 21 Years

**DOI:** 10.1002/mdc3.70368

**Published:** 2025-10-22

**Authors:** Sacha E. Gandhi, Katherine A. Grosset, Prasanth A. Iruthayaraj, Romel Gravesande, Lance Lee, Cathal Doyle, Yoav Ben‐Shlomo, Donald G. Grosset

**Affiliations:** ^1^ School of Neuroscience and Psychology University of Glasgow Glasgow UK; ^2^ Parkinson's UK; ^3^ Population Health Sciences, Bristol Medical School University of Bristol Bristol UK

**Keywords:** Parkinson's, parkinsonism, prevalence, incidence, survival

## Abstract

**Background:**

Findings are contradictory regarding changes in the incidence and prevalence of Parkinson's disease (PD) over time; data for other parkinsonian disorders are rare.

**Objectives:**

To analyze temporal trends in the incidence and prevalence of eight parkinsonisms (PD, MSA, PSP, CBS, DLB, vascular parkinsonism, drug‐induced parkinsonism, and other secondary parkinsonism) from 2003 to 2023, assess their survival, and define sociodemographic factors associated with PD.

**Methods:**

Analysis of primary and linked secondary care data in a population‐based UK observational study using multivariable regression models and Kaplan–Meier survival methods.

**Results:**

Age‐ and sex‐adjusted incidence and prevalence rates of rarer parkinsonisms increased substantially pre‐Covid‐19, while adjusted PD rates declined then stabilized. From 2003 to 2010, the incidence of rarer parkinsonisms increased annually by 13.8% (95% CI 13.5, 14.0%), while PD incidence declined annually by 4.7% (4.5, 4.9%). From 2011 to 2019, the incidence of rarer parkinsonisms increased annually by 3.2% (2.9, 3.5%) while PD incidence changed by −0.3% (−0.5, 0.0%) annually. Prevalence fell for PD from 2003 to 2019 by 0.6% annually (0.6, 0.7%), but increased for other parkinsonisms by 7.1% (6.8, 7.3%) before slowing to 3.3% (3.1, 3.5%) annually. Of the degenerative parkinsonisms, 5‐year survival was best for PD at 64.2% (64.0, 64.4%) and worst for PSP at 30.5% (29.3, 31.8%). Adjusted PD incidence was 43% lower (16, 62%) in individuals of African/Caribbean ethnicity, but similar for Asian and White ethnicities.

**Conclusions:**

Increasing diagnosis of rarer parkinsonisms has contributed to changing temporal trends for PD. Ethnic variations for PD justify further exploration.

Incidence studies in Parkinson's disease (PD) have shown conflicting findings of an increase,^1^, ^2^, ^3^ no change^4^, ^5^, ^6^, ^7^ and a decrease over time^4^, ^8^, ^9^, ^10^, ^11^ (Supplementary Table S1). While increasing PD prevalence is reported worldwide even after adjustment for increased life expectancy,^12^ temporal changes between and within regions are markedly heterogenous.^13^ A global increase of 22% in age‐standardized PD prevalence was estimated between 1990 and 2016 using studies with different designs, case ascertainment and generally small case numbers.^12^ These methodological limitations question the reliability of the calculations. Secular incidence and prevalence trends for other parkinsonian disorders are largely unknown. Most prior studies have investigated individual parkinsonian disorders^2^, ^3^, ^4^, ^8^, ^10^, ^11^, ^14^; a few smaller studies combined PD with other types^1^, ^15^ or pooled findings across rarer subtypes.^9^

We hypothesized that temporal changes in the diagnosis of parkinsonian disorders could affect PD incidence and prevalence rates. We also analyzed comparative survival for eight parkinsonian disorders, which has never been undertaken previously.

Additionally, we examined sociodemographic features linked to PD risk. Rurality^16^ and lower deprivation levels^4^, ^8^, ^17^, ^18^, ^19^, ^20^ are inconsistently associated with a higher likelihood of PD.^3^, ^7^, ^16^, ^21^ Lower rates in sub‐Saharan Africa and Asia compared with Europe, North and Latin America are difficult to attribute to environmental or genetic factors due to methodological variation.^12^, ^22^, ^23^, ^24^, ^25^, ^26^, ^27^, ^28^, ^29^ Prior UK‐wide studies have not assessed ethnicity in relation to PD risk and this was a further aim.

## Methods

This population‐based observational study analyzed data in the Clinical Practice Research Datalink (CPRD) Gold and Aurum from Jan 1, 2003, to Dec 31, 2023, using the June 2024 datasets. CPRD contains anonymized data for 69 million people over 38 years, is broadly representative of the UK population,^30^ and included 27.2% of the UK population aged 20 years and over in 2023. Primary care data were the main data source and were enriched with patient data from Hospital Episodes Statistics (HES) and death registration data (Version 2022.01.001) from the Office of National Statistics (ONS). Cases were registered for at least 6 months and validated by CPRD as having acceptable data quality. The protocol was approved by CPRD's Research Data Governance Process.

We investigated the most common parkinsonism diagnoses: PD, Multiple System Atrophy (MSA), Progressive Supranuclear Palsy (PSP), Corticobasal Syndrome (CBS), Dementia with Lewy Bodies (DLB), vascular parkinsonism (VP), drug‐induced parkinsonism (DIP) and designated other secondary parkinsonism (OSP) to include rarer types (eg, post‐encephalitic parkinsonism, syphilitic parkinsonism, carbon monoxide poisoning). Two experts, KAG and DGG, defined diagnostic codes from the two systems of standardized clinical terms used in CPRD (Systemized Nomenclature of Medicine (SNOMED) and the Read code system). These codes are recorded in primary care but are invariably based on physician diagnoses from secondary care attendance with a neurologist or medicine for the elderly specialist, mostly in movement disorders clinics. Equivalent International Classification of Diseases tenth revision (ICD‐10) codes were obtained from HES and ONS (Supplementary Table S2, supplementary methods). Imputation was used to predict PD where subjects were prescribed dopaminergic medication but had no diagnosis recorded to explain this therapy (eg, parkinsonian disorder, restless legs syndrome, pituitary tumor, see supplementary methods). Coded and imputed cases were compared (Table S3).

Covariates were selected from previous studies^4^, ^16^, ^24^, ^31^, ^32^, ^33^: age, self‐reported sex, ethnicity, geographical region, rurality and, for England, socioeconomic status, proxied by an area‐based measure of deprivation. Ethnicity was defined from a CPRD algorithm combining primary and secondary care data (supplementary methods). Patient‐level socioeconomic status used 2023 Index of Multiple Deprivation (IMD) quintiles, a composite census‐derived measure of relative deprivation, based on geographic areas of around 1500 people (one most deprived to five least deprived)^34^ (supplementary methods). Rurality was determined at a GP level using the Rural–Urban classification.^35^

## Statistical Methods

Crude annual incidence rates were calculated as the number of new cases divided by the total person‐years in the CPRD sample population. Crude point prevalence rates were calculated for July 1st in each year out of all patients registered. Rates were expressed per 100,000 of the total CPRD population (all ages) and standardized to the 2013 European Standard Population. As HES data were only available in 82% of people in England, pro‐rated adjustments were applied using age‐ and sex‐specific rates. Findings were scaled to national census data and projections to estimate UK case numbers.

Visual inspection and Python *statsmodels* evaluated the best fit for secular patterns from 2003 to 2019. For incident and prevalent PD, Poisson regression models were used due to minimal overdispersion; for incidence a spline knot at 2010 minimized the Akaike Information Criterion value. For the other seven parkinsonisms, the fit of Poisson and negative binomial models was poor (large residuals), so linear Ordinary Least Squares models were fitted. Optimum spline points were 2013.5 for prevalence and 2010.5 for incidence. Model coefficients from the later periods were used to generate future projections. Male to female incidence and prevalence rate ratios (RR) were calculated using crude case counts (Poisson, or negative binomial when data overdispersed) adjusted for age and year.

For survival analyses, two controls without a parkinsonian disorder or dopaminergic medication were randomly matched by age (± 2 years), sex and GP practice, using incidence density sampling. Kaplan–Meier survival plots were censored at the last observation, transfer out date, or date of death. Follow up was partitioned into three 7‐year time intervals due to non‐proportionality over time; multivariable Cox regression calculated adjusted hazard ratios (HRs) for each period (age band at diagnosis, sex, and case/control as covariates). Life expectancy was calculated by age (in 5‐year age bands) and by sex from internally derived life tables, using the number of deaths and population years at risk.

We used Poisson regression to calculate effect estimates (rate ratios, 95% CIs, P values) for the pre‐specified risk factors for PD incidence and prevalence separately. UK nation was incorporated without deprivation data as these were only available for England. The multivariable models mutually adjusted for all included covariates. We tested for “a priori” interactions between age and (sex, rurality), sex and rurality, and ethnicity (Asian and African‐Caribbean) and (age, rurality), hypothesizing that associations would be stronger in older than younger patients and in men than women. We undertook deterministic sensitivity analyses for ethnicity, hypothesizing that cases from ethnic minorities would be more likely to have missing codes than Whites (supplementary methods). Statistical analyses were conducted in Python (version 3.11.3) and R (Version 2024.09.0 + 375). Findings are presented in accordance with RECORD recommendations.^36^

## Results

### Secular Trends in Incidence Rates

**Figure 1:**
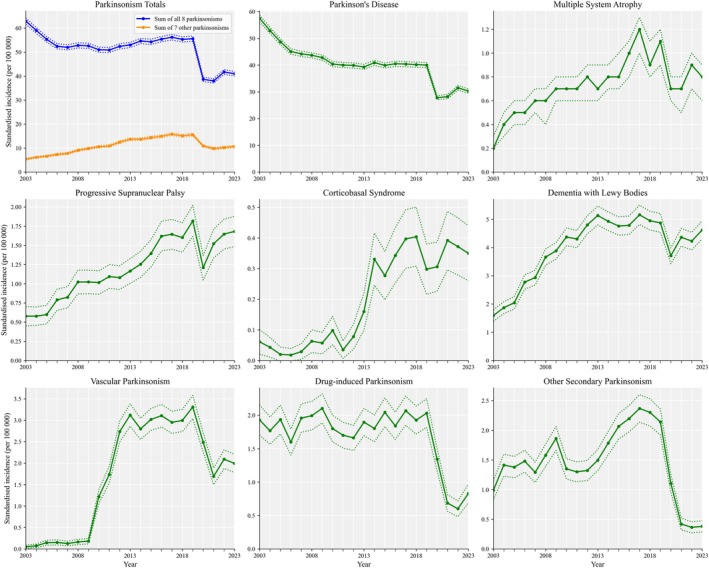
Incidence of parkinsonian disorders in the United Kingdom from 2003 to 2023. From 2003 to 2019, the incidence of Parkinson's disease declined, while other parkinsonisms increased, except for drug‐induced. Incidence rates are per 100,000 person‐years at risk and are age‐ and sex‐standardized to the 2013 European Standard Population.

Between 2003 and 2023, there were 173,560 new parkinsonism diagnoses (135,637 had PD) and 367,887,266 person‐years of observation. From 2003 to 2010, PD incidence declined by 4.7% per year (4.5, 4.9%) while the incidence of the other seven parkinsonisms increased annually by 13.8% (13.5, 14.0%). Combining all eight parkinsonisms there was an annual reduction of 2.0% (1.8, 2.2%). From 2011 to 2019, the annual change in PD incidence was −0.3% per year (−0.5, 0.0%) while the incidence of the other seven parkinsonisms increased annually by 3.2% (2.9, 3.5%) (Fig. 1); combining all eight parkinsonisms there was an annual increase of 0.6% (0.4, 0.8%). The incidence of parkinsonian disorders declined after 2019 and remained lower than pre‐Covid levels through to 2023, despite some signs of recovery. For PD, incidence declined by 20.4% (14.2, 26.1%), comparing the 4 years before and after the onset of Covid‐19 (ie, 2016–2019 compared to 2020–2023).

### Secular Trends in Prevalence Rates

**Figure 2:**
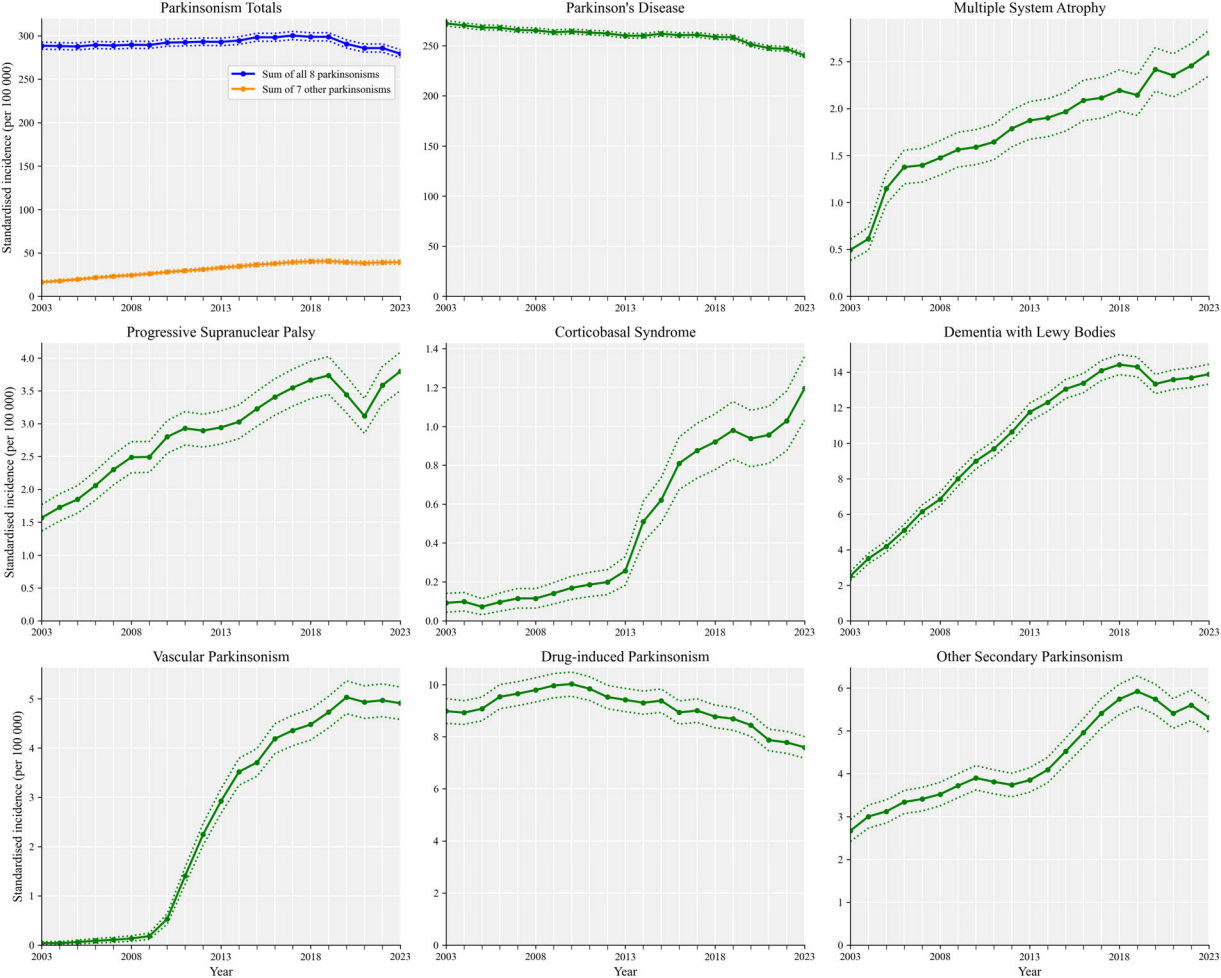
Prevalence of parkinsonian disorders in the United Kingdom from 2003 to 2023. The prevalence of Parkinson's disease declined slightly from 2003 to 2019, while other parkinsonisms increased, except for drug‐induced. Prevalence rates are per 100,000 of the population  and are age‐ and sex‐standardized to the 2013 European Standard Population.

For prevalence, the study sample varied between 16,107,943 and 18,092,540 people per year. From 2003 to 2019, PD prevalence rates fell annually by 0.6% (0.6, 0.7%) while, for the other seven parkinsonisms, the annual increase was 7.1% (6.8, 7.3%) between 2003 and 2013, and 3.3% (3.1, 3.5%) between 2014 and 2019 (Fig. 2). Combining all eight parkinsonisms the annual change was 0.02% (−0.1, 0.1%) from 2003 to 2019. The fall in PD prevalence was 3.1% (0.4, 5.8%) before to after the onset of Covid‐19.

**Figure 3:**
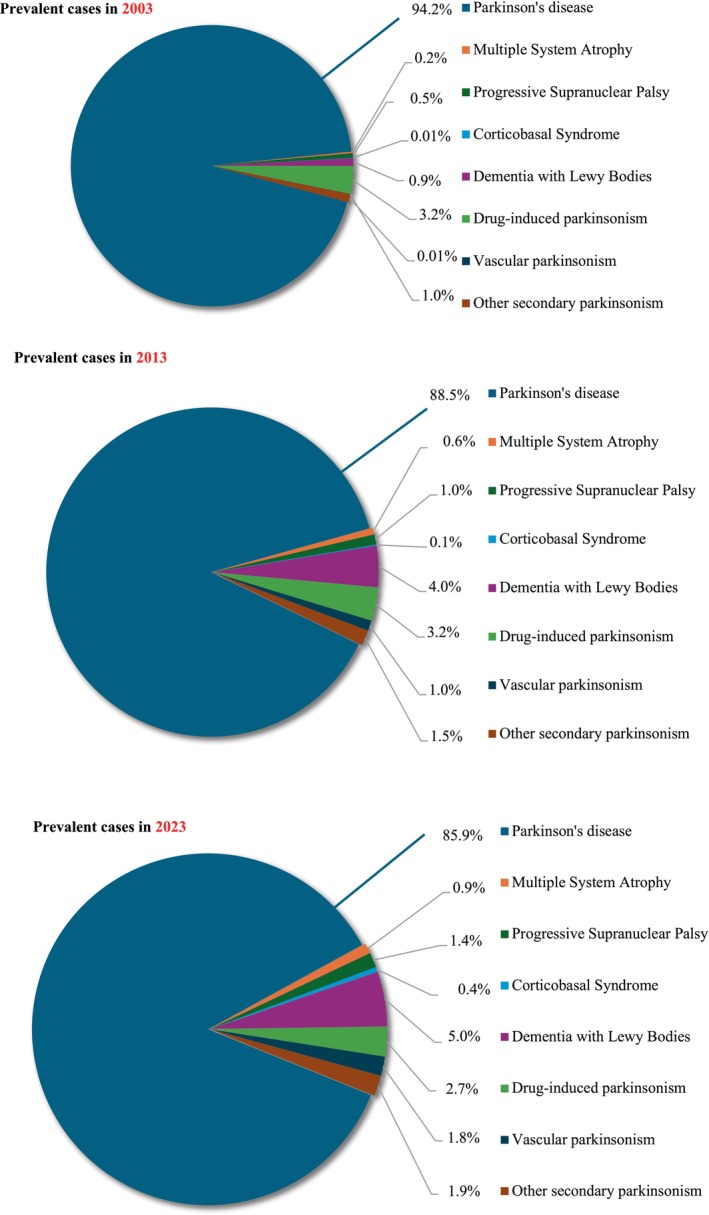
Proportion of prevalent cases of parkinsonism in the United Kingdom comparing 2003, 2013 and 2023. There was a decline in the proportion of cases with Parkinson's disease, while the proportion with other parkinsonisms, particularly Dementia with Lewy Bodies, increased.

The proportion of prevalent parkinsonism that was PD decreased from 94.2% in 2003 to 85.9% in 2023, with a corresponding increase in atypical parkinsonian disorders, being largest for DLB (Fig. 3). The increase in other parkinsonian disorders was due to both de novo diagnosis and diagnostic revision; 6.1% of incident cases with degenerative parkinsonism had their diagnoses revised, most commonly from PD to DLB (Supplementary Table 4).

### Secular Trends in Absolute Numbers of Cases

Absolute numbers of people with incident PD declined between 2003 and 2010 and then increased until Covid‐19 (Fig. S1). Prevalent PD numbers increased progressively until 2019, reflecting the increasing population size with proportionately more older people (Fig. S1). In 2023, there were an estimated 30,693 incident parkinsonism cases (23,446 incident PD) and 188,743 prevalent parkinsonism cases (162,121 prevalent PD). Future projections gave an estimate for 2025 of 31,469 incident parkinsonism (23,890 incident PD) and 193,225 prevalent parkinsonism cases (165,186 prevalent PD). For 2030 the estimates were 32,502 incident parkinsonism (24,238 incident PD) and 204,103 prevalent parkinsonism cases (172,505 prevalent PD) (Fig. S2).

### Survival Analyses

**Figure 4:**
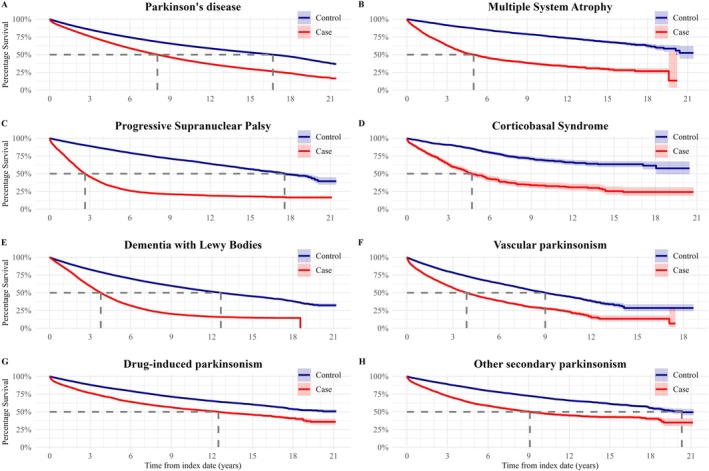
Kaplan–Meier curves of survival for eight parkinsonisms in the UK over 21 years. For each type of parkinsonism, survival was shorter for cases than their matched controls. Heavy dotted lines represent median survival in years (not shown where median survival was greater than 21 years).

**Figure 5:**
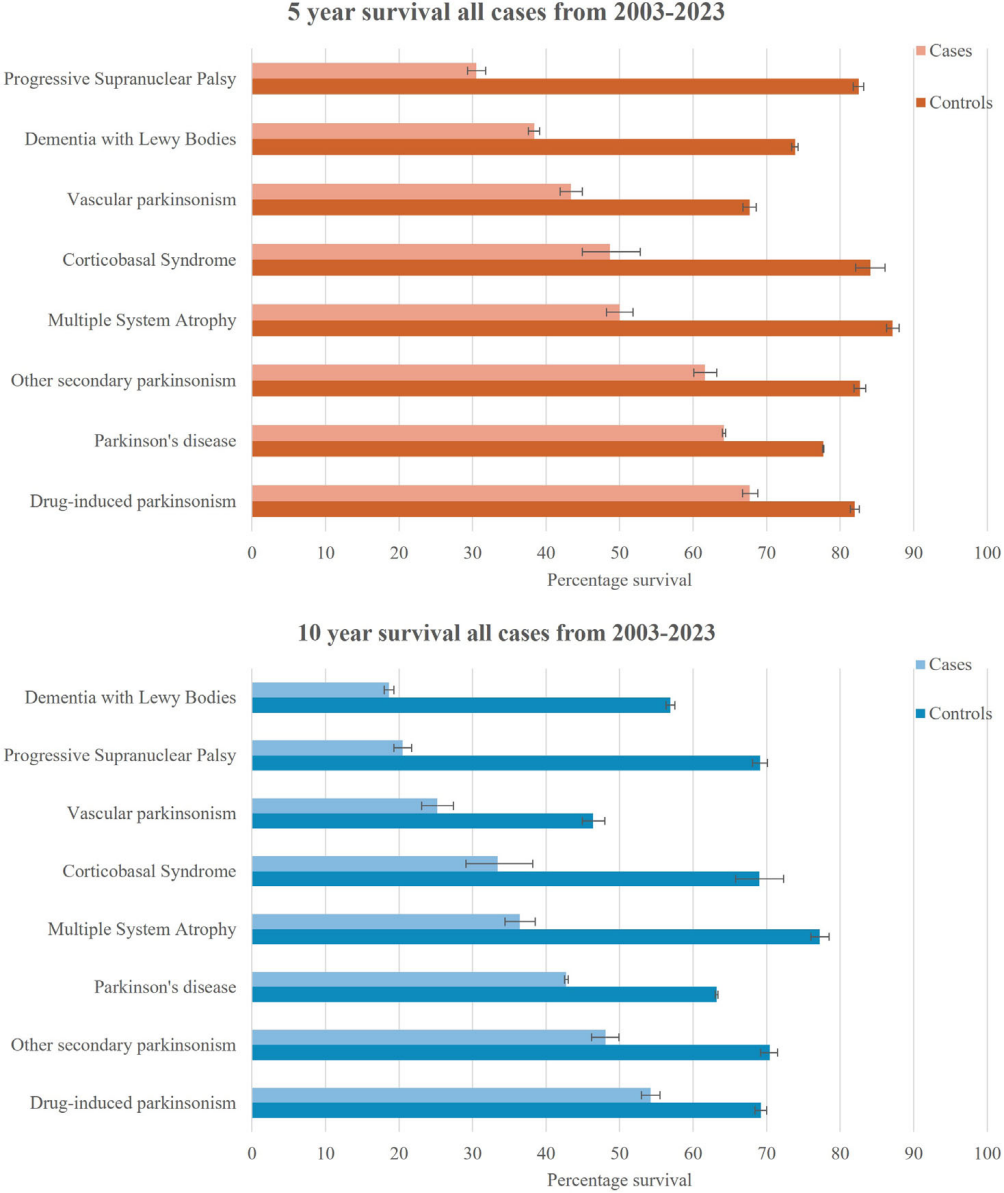
Five‐ and 10‐year percentage survival for PD and other parkinsonisms compared to matched controls from 2003 to 2023. Parkinsonism cases had worse survival than matched controls. Survival for controls varied due to age and sex differences (eg, vascular parkinsonism cases and controls were around six years older than Parkinson's disease cases and controls). Diagnoses are ordered by percentage survival.

For each parkinsonian disorder, survival and life expectancy were shorter for cases than controls (Figs. 4 and 5, Table S5–S7). Younger onset cases had longer life expectancy than older onset cases as expected, but the absolute differences in life expectancy between cases and controls decreased with increasing age (Fig. S3, Table S7). Of the degenerative parkinsonisms, 5‐year survival was best for PD at 64.2% (64.0, 64.4%) and worst for PSP at 30.5% (29.3, 31.8%). The risk of death for PD was greater in men and increased with older age at diagnosis (Table S8).

### Comparative Incidence and Prevalence of Parkinsonisms

In 2023, 7632 cases had a new diagnosis of parkinsonism, of whom 5832 had PD (76.4%), out of a population with 17,244,523 person years (Table 1, S9). This gave a European standardized incidence of 45.3 (95% CI 44.3, 46.3) for all parkinsonisms and 34.7 (95% CI 33.8, 35.6) for PD per 100,000. On July 1, 2023, there were 47,925 cases with prevalent parkinsonism, including 41,141 with PD, in a population of 18,092,540. This gave a European standardized prevalence of 279 (95% CI 277, 282) for all parkinsonisms, and 240 (95% CI 238, 242) for PD per 100,000 (Table 2, S10). Other parkinsonian disorders had substantially lower incidence and prevalence than PD; rates, demographic and clinical features are in Tables 1 and 2; S9–S11.

**TABLE 1:** Demographic and clinical features of incident degenerative parkinsonisms in 2023

	Parkinson's disease	Multiple system atrophy	Progressive supranuclear palsy	Corticobasal syndrome	Dementia with Lewy bodies
Number of cases (% of all cases)^a^	5832 (76.4%)	127 (1.7%)	282 (3.7%)	59 (0.8%)	787 (10.3%)
Crude incidence (95% CI)	33.8 (33.0, 34.7)	0.74 (0.61, 0.88)	1.6 (1.5, 1.8)	0.34 (0.26, 0.44)	4.6 (4.3, 4.9)
Standardized incidence (95% CI)	34.7 (33.8, 35.6)	0.76 (0.63, 0.90)	1.7 (1.5, 1.9)	0.35 (0.26, 0.44)	4.6 (4.3, 4.9)
Age at diagnosis, mean (SD)	74.7 (9.3)	69.3 (10)	75.2 (8.5)	73.1 (10.1)	79.1 (6.8)
Sex					
Male, *n* (%)	3205 (65.2%)	62 (53.9%)	136 (57.6%)	19 (35.2%)	471 (59.8%)
Female, *n* (%)	1710 (34.8%)	53 (46.1%)	100 (42.4%)	35 (64.8%)	316 (40.2%)
Smoking status					
Current, *n* (%)	463 (9.4%)	10 (8.7%)	26 (11.0%)	6 (11.1%)	74 (9.4%)
Former, *n* (%)	3112 (63.3%)	73 (63.5%)	149 (63.1%)	29 (53.7%)	519 (65.9%)
Never, *n* (%)	1314 (26.8%)	31 (27.0%)	60 (25.4%)	19 (35.2%)	190 (24.2%)
Missing, *n* (%)	26 (0.5%)	1 (0.9%)	1 (0.4%)	0 (0.0%)	4 (0.5%)
BMI kg/m^2^, mean (SD)	26.8 (5.0)	26.0 (6.0)	26.3 (5.0)	25.8 (5.3)	26.0 (5.4)
Dopaminergic medication					
Current, *n* (%)	4043 (82.3%)	70 (60.9%)	157 (66.5%)	31 (57.4%)	219 (27.8%)
Previous, *n* (%)	21 (0.4%)	2 (1.7%)	8 (3.4%)	4 (7.4%)	18 (2.3%)
Never, *n* (%)	851 (17.3%)	43 (37.4%)	71 (30.1%)	19 (35.2%)	550 (69.9%)
Current drug class					
L‐dopa, *n* (%)	3924 (79.8%)	67 (58.3%)	150 (63.6%)	31 (57.4%)	210 (26.6%)
Dopamine agonist, *n* (%)	249 (5.1%)	14 (12.2%)	11 (4.7%)	3 (5.6%)	25 (3.2%)
MAOB inhibitor, *n* (%)	170 (3.5%)	7 (6.1%)	7 (3.0%)	0 (0.0%)	10 (1.3%)
COMT inhibitor, *n* (%)	63 (1.3%)	5 (4.3%)	6 (2.5%)	1 (1.9%)	6 (0.8%)
Amantadine, *n* (%)	14 (0.3%)	8 (7.0%)	13 (5.5%)	4 (7.4%)	4 (0.5%)

^a^ Degenerative plus non‐degenerative parkinsonisms. Rates are per 100,000; standardization is to the European Standard Population 2013. Percentages may not sum because of rounding. Case numbers and rates include an uplift based on hospital admissions; other variables do not include an uplift.

Abbreviations: BMI = body mass index; CI = confidence interval; COMT = catechol‐O‐methyltransferase; MAOB = monoamine oxidase type B; SD = standard deviation; NA = not applicable.

**TABLE 2:** Demographic and clinical features of prevalent parkinsonism in 2023

	Parkinson's disease	Multiple system atrophy	Progressive supranuclear palsy	Corticobasal syndrome	Dementia with Lewy bodies
Number of cases (% of all cases)^a^	41,141 (85.8%)	445 (0.9%)	649 (1.3%)	204 (0.4%)	2407 (5.0%)
Crude prevalence (95% CI)	227 (225, 230)	2.5 (2.2, 2.7)	3.6 (3.3, 3.9)	1.1 (1.0, 1.3)	13.3 (12.8, 13.8)
Standardized prevalence (95% CI)	240 (238, 242)	2.6 (2.4, 2.8)	3.8 (3.5, 4.1)	1.2 (1.0, 1.4)	13.9 (13.3, 14.4)
Age at diagnosis, mean (SD)	69.1 (11.7)	64.8 (13.2)	71.9 (12.1)	69.6 (11.0)	77.3 (7.8)
Sex					
Male *n* (%)	22,619 (58.7%)	219 (50.3%)	335 (51.8%)	85 (41.9%)	1292 (53.7%)
Female *n* (%)	15,925 (41.3%)	216 (49.7%)	312 (48.2%)	118 (58.1%)	1115 (46.3%)
Smoking status					
Current, *n* (%)	3923 (10.2%)	59 (13.6%)	64 (9.9%)	29 (14.3%)	204 (8.5%)
Former, *n* (%)	22,890 (59.4%)	243 (55.9%)	386 (59.7%)	120 (59.1%)	1531 (63.6%)
Never, *n* (%)	11,314 (29.4%)	126 (29.0%)	185 (28.6%)	50 (24.6%)	612 (25.4%)
Missing, *n* (%)	417 (1.1%)	15 (3.4%)	12 (1.9%)	4 (2.0%)	60 (2.5%)
BMI kg/m^2^, mean (SD)	26.3 (5.6)	26.2 (5.5)	25.8 (5.5)	25.8 (5.9)	25.6 (5.5)
Dopaminergic medication					
Current, *n* (%)	32,273 (83.7%)	191 (43.9%)	349 (53.9%)	67 (33.0%)	598 (24.8%)
Previous, *n* (%)	2068 (5.4%)	51 (11.7%)	95 (14.7%)	41 (20.2%)	97 (4.0%)
Never, *n* (%)	4203 (10.9%)	193 (44.4%)	203 (31.4%)	95 (46.8%)	1712 (71.1%)
Current drug class					
L‐dopa, *n* (%)	31,339 (81.3%)	169 (38.9%)	325 (50.2%)	59 (29.1%)	567 (23.6%)
Dopamine agonist, *n* (%)	7640 (19.8%)	40 (9.2%)	28 (4.3%)	10 (4.9%)	64 (2.7%)
MAOB inhibitor, *n* (%)	5493 (14.3%)	7 (1.6%)	8 (1.2%)	2 (1.0%)	20 (0.8%)
COMT inhibitor, *n* (%)	4705 (12.2%)	7 (1.6%)	15 (2.3%)	3 (1.5%)	13 (0.5%)
Amantadine, *n* (%)	1835 (4.8%)	32 (7.4%)	49 (7.6%)	10 (4.9%)	2 (0.1%)
Duration of therapy in years, mean (SD)	5.4 (5.0)	3.6 (3.5)	2.6 (2.9)	2.2 (2.8)	2.7 (3.0)

*Note*: Case numbers and rates include an uplift based on hospital admissions; other variables do not include an uplift.

Abbreviations: BMI = body mass index; CI = confidence interval; COMT = catechol‐O‐methyltransferase; MAOB = monoamine oxidase type B; SD = standard deviation; NA = not applicable.

^a^ Degenerative plus non‐degenerative parkinsonisms. Rates are per 100,000; standardization is to the European Standard Population 2013. Percentages may not sum because of rounding.

### Demographic Factors

The incidence and prevalence of parkinsonian disorders increased with age but then declined, usually after the ninth decade (Figs. S4 and S5). PD, MSA, PSP, DLB, VP and OSP were significantly more common in males than females; a female preponderance was observed for prevalent, but not incident CBS. DIP incidence and prevalence were similar between sexes (Table S12).

**Figure 6:**
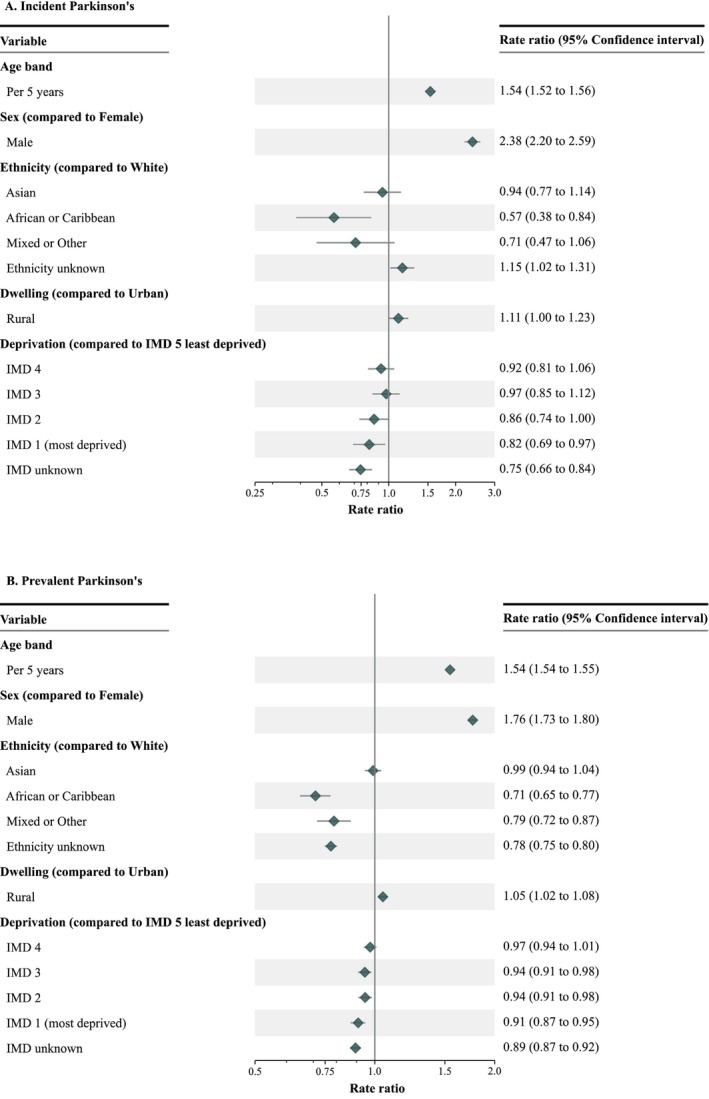
Factors relating to (A) incident and (B) prevalent Parkinson's disease in 2023. Older age, male sex, White and Asian ethnicity, rurality, and lower deprivation were associated with higher incidence and prevalence of Parkinson's disease. Individuals of African or Caribbean ethnicity had significantly lower incidence and prevalence. The models adjusted for all included covariates (ie, age, sex, ethnicity, rurality and deprivation). IMD: Index of Multiple Deprivation.

For PD, older age at diagnosis and male sex were strongly associated with higher incidence and prevalence (Fig. 6). Adjusted PD incidence, RR 0.57 (0.38, 0.84), and prevalence, RR 0.71 (0.65, 0.77), were significantly lower for individuals of African or Caribbean ethnicity. Adjusted prevalence was also lower for Mixed or Other ethnicities, RR 0.79 (0.72, 0.87), but incidence and prevalence were both equivalent for Asian and White ethnicities (Fig. 6). PD incidence and prevalence were higher in rural areas and declined with increasing deprivation (Fig. 6, Table S13). Prevalent PD was lower in Scotland, RR 0.86 (0.82, 0.90) than England, but equivalent to England in other UK countries (Table S11). Lower incidence and prevalence for African or Caribbean cases did not vary by age or rurality; no interactions were identified between age and sex or rurality, or between sex and rurality. Sensitivity analysis showed that substantial differential misclassification for African or Caribbean coding compared to Whites would be required to abolish the reduced PD risk (percent false negative coded as missing for African or Caribbean of 40% versus 1% for Whites).

## Discussion

This is the largest study comparing the incidence, prevalence and survival of PD and rarer parkinsonian disorders. It provides unique insights into evolving diagnostic patterns. We found that PD incidence declined initially pre‐Covid‐19 and then stabilized. Falling PD incidence in several studies^4^, ^8^, ^9^, ^10^, ^11^, ^37^ has been hitherto unexplained, although one study suggested that greater recognition of atypical parkinsonism might be contributory,^8^ which is precisely what we have confirmed. Other studies that found stable or increasing incidence rates for PD may reflect methodological variation including case definitions,^13^ as well as variation in the way diagnostic patterns evolved in different settings and secular periods. Our findings are inconsistent with estimated large increases in incident PD case numbers for the UK pre‐Covid‐19 in the Global Burden of Disease (GBD) analysis.^38^ GBD calculations are based on a steep increase in incident case numbers (eg, for 2003 they had 44.1% fewer incident case numbers than we estimated, and for 2019 they had 15.5% fewer). It is likely that combining data from smaller studies with differing designs has produced an unreliable estimate of rates of change.

The most directly comparable study to ours is the Rochester Epidemiology Project, which, in contrast, found an increase in pre‐Covid‐19 PD incidence rates over time for men but not women,^1^ while MSA^39^, ^40^ and PSP^15^, ^40^ rates were stable, although their small population size (<150,000) made the study underpowered for atypical disorders.

Potential reasons for evolving diagnostic approaches in the UK include improved diagnostic criteria, more specialists, and greater use of imaging; presynaptic dopaminergic imaging became available in 2000 and may have improved the recognition of benign tremors, DLB and VP^41^. In the UK, DLB diagnosis improved through the Diamond Lewy study^42^; an increase in DLB case numbers likely includes cases previously considered as Alzheimer's disease. As we considered DLB diagnostic codes made over 1 year after a PD diagnosis as Parkinson's disease dementia (PDD) rather than DLB, miscoding of PDD as DLB would not account for the observed increase in DLB over time. Given the evolving diagnostic patterns over time, considering PD alongside other parkinsonisms is therefore suggested in future studies of secular trends.

For prevalence, a global rise has been reported but is much lower in high income countries.^24^, ^38^ However, comparing different studies even within the same country has limitations.^32^ Meta‐analysis of eight UK studies in 2009 noted between‐study heterogeneity but found no temporal changes over the preceding 42 years.^43^ Our secular model suggested prevalence declined by 0.6% per year pre‐Covid‐19. Our numbers for prevalent PD cases in the UK were around 13% lower than those estimated by GBD for 2003 and around 15% lower for 2019.^38^ However, we found a 1.4% annual increase from 2003 to 2019 in the absolute numbers of people with PD attributable to the increasing population size with proportionate increases in older people.

Incidence and prevalence rates vary considerably in other studies (Table S1). Our 2023 prevalence estimate of 240 per 100,000 is higher than earlier UK prevalence studies,^31^, ^32^ likely reflecting methodological differences. Prior UK studies were substantially smaller and analyzed varying combinations of primary and secondary care data at a regional rather than national level.^32^ The only prior UK‐wide PD prevalence study^31^ predated a major expansion in the CPRD dataset and did not include linked data or cases imputed from dopaminergic therapy. National multisource healthcare database system analyses are likely to be more inclusive and therefore give higher rates.^44^, ^45^, ^46^, ^47^ However, broad inclusion of dopaminergic medication prescriptions to define PD^4^, ^8^, ^12^, ^48^ rather than our selective imputation approach appears to substantially overestimate rates. Around 10% of people with PD never received dopaminergic therapy, which is about half the rate in a prior UK analysis,^49^ reflecting a trend towards earlier treatment post diagnosis. For MSA 44% never received dopaminergic therapy, which is higher than the 27% of cases from a specialist center^50^ and may be affected by the proportion of cases with predominantly cerebellar features.

Decreased rates of parkinsonism since Covid‐19 were much greater for incidence than prevalence, suggesting that reduced healthcare access was a stronger contributor than increased mortality to rate changes. This is consistent with large incidence rate reductions for several other long‐term conditions.^51^, ^52^, ^53^

To our knowledge, this is the first study to examine the effects of ethnicity on PD epidemiology across the UK. A smaller east London study found no overall relationship between PD and ethnic group.^54^ Lower PD incidence and prevalence in people of African or Caribbean ethnicity compared to White ethnicity confirms findings from previous US record‐based studies^55^, ^56^, ^57^ and was not explained by age or rurality. Whilst ethnicity coding was missing in around 10% of records, sensitivity analyses suggested that it is implausible that this could have artefactually produced such a large effect. One US study reported that Black males had over double the incidence of White males and a lower prevalence,^58^ potentially reflecting reduced survival or methodological issues in defining the size of the denominator population.^56^ Studies within Africa found even lower rates^25^, ^27^, ^28^, ^29^ but may have ascertainment bias from reduced healthcare access and inadequate adjustment for deprivation. A door‐to‐door study in the US and Nigeria found age‐adjusted PD prevalence to be five times higher in Black Americans than in those residing in Nigeria, suggesting a greater relative contribution from environmental than genetic factors.^59^

Lower PD prevalence in African or Caribbean populations may reflect lower rates of genetic mutations and risk variants: *GBA1* variants vary substantially across populations,^60^ and the *LRRK2 Gly2019Ser* mutation may be lower in sub‐Saharan Africa than in Europe and North Africa.^61^, ^62^, ^63^, ^64^, ^65^ Our findings regarding PD risk in British Asians are novel; the lower reported PD prevalence in Asians^24^, ^33^ was not seen in British Asians. Differing case definitions and case ascertainment might explain this.^33^ Taken together, the observations on PD risk related to ethnicity suggest varying effects in different populations, which requires further research exploring genetics, environmental factors and differential access to healthcare for individuals of non‐European origin.

Scotland had a 14% lower prevalence than other UK nations in 2023, potentially contributed by higher age‐standardized mortality from both cardiovascular disease and Covid‐19, a possible effect of higher cigarette smoking, assuming this is causally protective, greater use of dopamine transporter scans, and lower rates of PD risk genes, which may merit further specific study. Although we were unable to adjust for deprivation in the model including UK nation as it was unavailable in CPRD data outside England, deprivation levels are equivalent in Scotland and England, and deprivation alone would not explain the magnitude of the difference. We confirmed previous findings that PD is more common in the least deprived^4^, ^8^ and in rural settings.^16^ For rurality, effects were not greater in men than women, suggesting that associations were not due to higher occupational exposures in men alone.

We quantified the effects of female longevity on the sex ratio of incident and prevalent PD: there were around 6% fewer men with prevalent than incident PD, and absolute numbers of women with prevalent PD exceeded men above age 90 years. Average age at diagnosis was around 6 years younger for prevalent than incident PD due to selective survival of younger cases. Men had higher standardized incidence and prevalence than women for PSP and VP, confirming previous findings,^21^, ^66^ and this was also true for MSA, DLB and OSP.

Median survival for PD was around 1.6 years less than a meta‐analysis of mortality studies up to 2012.^67^ This may reflect our older more representative sample and the inclusion of non‐inception cohorts in the meta‐analysis, which will under‐represent cases with poorer outcomes. Our hazard ratios for risk of death are, however, consistent with two meta‐analyses of pooled mortality data.^67^, ^68^ We quantified this for different age bands, and findings are consistent with indirect life expectancy calculations.^69^, ^70^ PD cases aged under 50 years at diagnosis lose 10 or more years of life compared to less than 5 years in those aged over 75 years at diagnosis. Reducing this PD mortality gap is a key outcome for future research. For DLB, our median survival of 3.8 years was slightly lower than the 4.1 years reported in a meta‐analysis.^71^ Shorter survival for other degenerative parkinsonisms in our study likely relates to differences in age and study design, as most prior survival estimates are from small retrospective hospital‐based studies of younger cases, and many report survival after symptom onset rather than after diagnosis.^72^, ^73^, ^74^, ^75^, ^76^, ^77^

### Limitations

Case ascertainment from routine healthcare coding without clinical re‐examination or autopsy confirmation will contribute to diagnostic error, which might affect differences such as those found in relation to ethnicity. PD diagnoses in CPRD have 90% validity,^78^ but there are no validation studies for other parkinsonian disorders. Some cases coded as PD may have atypical features insufficient to fulfill an alternative diagnosis, which might result in overestimation of PD numbers. Although our imputation models were accurate at predicting PD based on dopaminergic medication use and key discriminating features, some imputed PD cases may have an alternative explanation for their medication use. Including smoking as a covariate was not possible as it was not provided in the denominator data; the association of smoking with both reduced PD risk and greater deprivation might partly explain the association between lower PD prevalence in more deprived areas. Rurality was determined at a GP practice rather than patient‐level, so some PD patients living rurally will be registered at an urban practice and vice versa.

### Conclusion

Our findings present several new and important observations that are generalizable to the UK and suggest the need for similar analyses in other countries. Considering rates of change in incidence and prevalence for all eight types of parkinsonism combined in the pre‐Covid years, the changes observed in incidence and prevalence rates for PD were predominantly due to greater recognition of rarer types of parkinsonism. Lower rates of PD in people of African or Caribbean ethnicity, but equivalent rates between those of Asian and White ethnicity, require further exploration including more detailed subgroup analyses. The major adverse impact of a diagnosis of parkinsonism on survival reinforces the need for treatments to reduce both comorbid complications and rates of progression. The projected rise in the numbers of people living with parkinsonian disorders will necessitate progressively greater healthcare provision and social care input.

## Author Roles

(1) Research project: A. Conception, B. Organization, C. Execution; (2) Statistical Analysis: A. Design, B. Execution, C. Review and Critique; (3) Manuscript Preparation: A. Writing of the first draft, B. Review and Critique.

S.E.G.: IB, IC, 2A, 2B, 2C, 3A, 3B.

K.A.G.: 1A, 1B, 1C, 2A, 2C, 3B.

P.A.I.: 1B, 1C, 2A, 2B, 2C, 3B.

R.G.: 1B, 1C, 2C, 3B.

L.L.: 1B, 1C, 2A, 2B, 2C, 3B.

C.D.: 1A, 1B, 1C, 2C, 3B.

Y.B.S.: 1A, 1B, 1C, 2A, 2B, 2C, 3B.

D.G.G.: 1A, 1B, 1C, 2A, 2C, 3B.

## Disclosures

**Ethical compliance statement:** The protocol and access to the study data were approved by the CPRD's Research Data Governance Process. Informed patient consent was not necessary for this work. CPRD has research ethical approval from the UK Health Research Authority (HRA) Research Ethics Committee for observational public health research. We confirm that we have read the Journal's position on issues involved in ethical publication and affirm that the work is consistent with those guidelines.

**Funding Sources and Conflict of Interest:** This work was performed under a CPRD multi‐study license, paid for by Parkinson's UK, who defined the broad remit of the work. The academic team defined the design, analysis methods and interpretation of the data. Employees of Parkinson's UK worked collaboratively with the academic team at each stage of the project. The academic team had a primary role in writing the report and in the decision to submit for publication. Parkinson's UK provided grant funding for the Tracking Parkinson's study for which DGG and YBS were principal investigators; and the Bone Health Improvement in Parkinson's project for which DGG is a principal investigator. SEG received financial support from these grants for research time in preparation for the submission of a PhD thesis. SEG also received financial support for conference travel and attendance related to this work. KAG, YBS and DGG have received consultancy payments from Parkinson's UK related to this research. PAI, RG, LL are CD are employees of Parkinson's UK.

**Financial Disclosures for the Previous 12 Months:** DGG received consultancy payments from Parkinson's UK related and unrelated to this research. KAG and YBS received consultancy payments from Parkinson's UK related to this research. SEG received financial support from Parkinson's UK grants for research time in preparation for the submission of a PhD thesis. SEG also received financial support for conference travel and attendance related to this work. PAI, RG, LL and CD have no additional disclosures to report.

## Supporting information


**Data S1.** Supplementary methods. Supplementary methods describe data access, cleaning and linkage, sample size considerations, ethnicity and deprivation data, and the handling of quantitative variables. Additional details are provided for the deterministic sensitivity analysis, imputation model and diagnosis of Dementia with Lewy Bodies.

**TABLE S1.** Summary of incidence and prevalence studies of parkinsonism. Original research articles and reviews on the incidence, prevalence and secular trends are summarized for the eight parkinsonian disorders. Most studies investigated individual parkinsonian disorders; several smaller studies combined PD with other parkinsonisms or pooled findings across rarer subtypes.

**TABLE S2.** Diagnostic codes used for case definitions of eight types of parkinsonism. Case definitions for the eight parkinsonian disorders were based on diagnostic Read and SNOMED codes in CPRD, and International Classification of Diseases tenth revision (ICD‐10) codes in HES and ONS.
**TABLE S3.** Demographic and clinical features of coded and imputed PD cases. Coded and imputed cases of Parkinson's disease were compared by t‐test (numerical data) or Chi‐squared tests (categorical data). Imputed incident cases were older than coded cases, but imputed prevalent cases were younger and had a shorter treatment duration.
**TABLE S4.** Summary of diagnostic sequence for incident cases of degenerative parkinsonism, 2003 to 2023. From 2003 to 2023, diagnoses were revised for 6.1% of incident cases with degenerative parkinsonism and for 4.6% of incident cases of Parkinson's disease.
**TABLE S5.** Summary of case counts for Kaplan–Meier graphs of survival shown in Figure 4. The population at risk and number of deaths, corresponding to the Kaplan–Meier survival curves in Figure 4, are presented for cases and controls for each parkinsonian disorder over the 21‐years of follow‐up.
**TABLE S6.** Survival after a diagnosis of parkinsonism compared to matched controls. For each type of parkinsonism, median, 5‐ and 10‐year percentage survival were reduced for cases compared to their matched controls. Among the degenerative parkinsonisms, median and 5‐year survival were longest for Parkinson's disease and shortest for Progressive Supranuclear Palsy.
**TABLE S7.** Life expectancy estimates for eight types of parkinsonism by age and sex. Life expectancy values are at the start of the age interval. For all parkinsonian disorders, life expectancy was reduced for cases than controls. Greater differences in life expectancy between cases and controls were observed for younger than older onset cases.
**TABLE S8.** Extended Cox model of survival in Parkinson's cases versus matched controls. Hazard ratios are adjusted for all covariates. Reference groups are controls for diagnosis and under 65 years for age.
**TABLE S9.** Demographic and clinical features of incident non‐degenerative parkinsonism cases in 2023. The table describes each of the non‐degenerative parkinsonisms; the total column is a sum of all degenerative cases (Table 1) and the non‐degenerative cases. Percentage of cases is out of the overall total.
**TABLE S10.** Demographic and clinical features of prevalent non‐degenerative parkinsonian cases in 2023. The table describes each of the non‐degenerative parkinsonisms; the total column is a sum of all degenerative cases (Table 2) and the non‐degenerative cases. Percentage of cases is out of the overall total.
**TABLE S11A.** Incidence rates of parkinsonism in the United Kingdom in 2023. Incidence rates are expressed per 100,000 person years at risk; standardization is to the European Standard Population 2013.
**TABLE S11B.** Prevalence rates of parkinsonism in the United Kingdom in 2023. Prevalence rates are expressed per 100,000 of the population; standardization is to the European Standard Population 2013.
**TABLE S12.** Male to female incidence and prevalence rate ratios for the eight parkinsonian disorders. Incidence and prevalence rate ratios are based on person years in crude case counts from 2003 to 2023, adjusted for age and year. Most parkinsonian disorders, except CBS and DIP, were significantly more common in men.
**TABLE S13.** Sociodemographic factors in cases of Parkinson's disease in 2023. Incidence is calculated by person‐years; prevalence by persons. Percentage values are out of the total with known values. Data are not included for uplifted cases as they are not available. Rates are expressed per 100,000 of the total population.

**Figure S1.** Estimated numbers of incident and prevalent Parkinson's in the UK from 2003 to 2023 inclusive. Absolute case numbers with incident Parkinson's disease initially declined before increasing between 2003 and 2019. Case numbers declined in 2020, due to Covid‐19, but are recovering. Prevalent case numbers steadily increased until 2019, after which there was a slight fall.

**Figure S2.** Future projections of UK cases of incident and prevalent Parkinson's disease and other forms of parkinsonism. Future projections of total UK case numbers, based upon pre‐Covid temporal trends and predicted population numbers. A proportionate increase in older people is expected to increase case numbers. Data are mean and 95% confidence intervals.

**Figure S3.** Age‐stratified Kaplan–Meier plots for Parkinson's disease compared with controls. The difference in median survival between Parkinson's disease cases and matched controls decreased progressively with increasing age. Accordingly, the greatest impact of Parkinson's on survival was in the youngest cases.

**Figure S4.** Crude incidence of Parkinson's disease and other types of parkinsonism by age and sex. For all parkinsonisms, crude incidence increased with age but then began to decline. Crude incidence was higher in men than women, except for corticobasal syndrome and drug‐induced parkinsonism.

**Figure S5.** Crude prevalence of Parkinson's disease and other types of parkinsonism in 2023 by age and sex. Crude prevalence of parkinsonisms increased to a peak, then declined (numbers were very small for some diagnoses in the oldest age band). Corticobasal syndrome was more common in women, while the remaining parkinsonisms, except drug‐induced, were more common in men.

## Data Availability

We will deposit in tabular data summaries and key processing code used in Python and R in Github; this will be available at publication. Access to the raw data requires a license application to CPRD.
