# Supplementary material for: Comparative Analysis of the Incidence, Prevalence, and Survival of 8 Types of Parkinsonism in a Population‐Based Study with 367 Million Person Years of Observation over 21 Years
Source: Mov Disord Clin Pract. 2025 Oct 22;13(4):933–48. doi: 10.1002/mdc3.70368 (PMC13071333; doi:10.1002/mdc3.70368)
Supplement: Supplementary file 1 — Data S1. Supplementary methods. Supplementary methods describe data access, cleaning and linkage, sample size considerations, ethnicity and deprivation data, and the handling of quantitative variables. Additional details are provided for the deterministic sensitivity analysis, imputation model and diagnosis of Dementia with Lewy Bodies. [file MDC3-13-933-s008.pdf]

Supplementary Methods

Data access and cleaning

PAI, RG and DGG had direct access to the raw CPRD datasets. Data extracts were made independently by PAI/RG and by DGG, and these were compared for accuracy and consistency and any differences resolved by discussion. Cases included were classed as having quality data using parameters set by CPRD using their established algorithms. Data cleaning included removal of cases with nonsense dates of birth and cases with a death recorded in the Office of National Statistics (ONS) that predated an existing record in CPRD. For BMI data, cleaning involved removal of outlying (implausible) values that indicated mistyped entries or miscalculations.

Linkage

Linkage between CPRD, HES and ONS data was undertaken by NHS Digital using their established hierarchical system based on matching multiple variables within each dataset.

Sample size considerations

After excluding cases aged under 20 years and with less than 6 months of follow-up, data were available for around 18 092 540 million people in 2023. Sample size calculations indicated that, at 5% significance and 90% power, 3746 cases and controls were required at a 1:1 ratio to detect a 25% increase in the odds ratios across two quintiles of IMD score with either incidence or prevalence.

Read, SNOMED and ICD-10 diagnostic codes

The Read codes are five-character alphanumeric codes that have provided a comprehensive standardised, semi-hierarchical clinical classification system for electronic health records in the NHS since 1985[1]. However, they are being gradually superseded by the Systematised Nomenclature of Medicine Clinical Terms (SNOMED CT). The SNOMED CT system was first developed in 1999 by combining Read codes with the SNOMED Reference Terminology by the College of American Pathologists and is considered one of the most comprehensive and precise clinical classification systems globally[2]. It provides a common shared clinical language, improves upon the accuracy and consistency of recorded data and incorporates a broader range of terms than Read codes[2]. International Classification of Disease tenth edition (ICD-10) codes are used in Hospital Episodes Statistics and the Office of National Statistics.

Ethnicity

Using the most recent version of the CPRD ethnicity algorithm, primary and secondary care data were used to assign cases to one of the following categories: White ethnicity: British (White), Irish (White), any other White background. Asian ethnicity: Bangladeshi, Bangladeshi (Asian or Asian British), Chinese, Chinese (other ethnic group), Indian, Indian (Asian or Asian British), Pakistani, Pakistani (Asian or Asian British), any other Asian background. African or Caribbean ethnicity: African (Black or Black British), Black African, Black Caribbean, Caribbean (Black or Black British). Mixed or other ethnicity: White and Asian (mixed), White and Black

African (mixed), White and Black Caribbean (mixed), any other mixed background, Black other, any other Black background, any other ethnic group. Unknown: Not given, not known, not stated.

Index of multiple deprivation

CPRD does not have a direct individual measure of socioeconomic status (SES), so to account for potential confounding or mediation by SES one has to use an ecological measure of area deprivation whose source data is the census which is then linked to individual records at lower super output areas with population sizes of around 1 500 individuals. We used the Index of Multiple Deprivation (IMD) score which was ranked and grouped into quintiles from most deprived to least deprived. IMD is based on seven different domains: Income Deprivation, Employment Deprivation, Education, Skills and Training Deprivation, Health Deprivation and Disability, Crime, Barriers to Housing and Services, Living Environment Deprivation. The domains are combined according to their respective weights into a single score[3].

Handling of quantitative variables

For the extended Cox survival model, cases and controls were grouped by age at diagnosis into the following predefined categories on a pragmatic basis: under 65 years, 65 to 74 years, 75 to 84 years, and 85 years and older, with cases under 65 years set as the reference group. Absolute estimates of remaining life expectancy were calculated for each 5-year age band consistent with previously established methodology[4, 5]. Other quantitative variables that were not grouped were treated as continuous variables.

Deterministic sensitivity analysis

Having observed an inverse association between African or Caribbean ethnicity and incidence/prevalence of PD, we explored if this could have been generated by miscoding of ethnicity, as around 13% of subjects had missing ethnicity coding. We used the methods proposed by Greenland (1996)[6] and implemented in Stata using the "episensi" command using incidence rate data as written by Orsini and colleagues[7]. We assumed that there was no false positive rate (specificity=100%) for both Black and White subjects but there was differential misclassification for sensitivity so that Black subjects were more likely to be coded as missing (false negative) than White subjects. We then examined a range of possible assumptions: we assumed that the sensitivity of White subjects was 99% and reduced the sensitivity of Black subjects until we attenuated the association completely to check empirically the degree of miscoding required to generate a biased association that we had observed.

Imputation model for cases prescribed dopaminergic therapy

Patients with at least one prescription of L-dopa, a dopamine agonist, a monoamine oxidase type B (MAOB) inhibitor, catechol-o-methyltransferase (COMT) inhibitor, or amantadine, and without an explanatory diagnostic code (i.e. one of the parkinsonian disorders, restless legs syndrome, pituitary tumour, dystonia, or neuroleptic malignant syndrome) in CPRD, HES or ONS were defined. For incidence, prescriptions from 2003 to 2023 inclusive were included, while for prevalence prescriptions in the 12 months to Jul 1, 2023 were included. The imputation models used cases with an explanatory diagnostic code, and a random 70:30 train:test split. Features included were based on known differences according to diagnosis and standard therapeutic approaches: age, sex,

smoking history, body mass index (BMI), age at first therapy, symptoms, and for medication, each of treatment duration, drug class, and dose expressed as levodopa-equivalent daily dose (LEDD) by standard formulas[8, 9]. Multiple Imputed Chained Equations (MICE) imputation was performed using the ImputationKernel from the miceforest package for missing BMI values (incidence 11.0%, prevalence 3.3% of cases) and smoking (incidence 1.9%, prevalence 1.1% of cases), with the random_state parameter set to forty-two and iterations set to five. Common symptoms noted in primary care records were compared between treated PD and other parkinsonian disorders, and the twelve symptoms with the highest percentage difference were each included as binary variables. Geographical region and source database (Gold or Aurum) were included as possible confounding factors. The imputation was performed using the RandomForestClassifier version 0.22 (Python scikit-learn; default settings including one hundred estimations). In the train:test stage for incidence the model identified PD with 0.79 sensitivity, 0.96 specificity, 0.85 precision, and with an F1-Score of 0.86. In the train:test stage for prevalence the model identified PD with 0.95 sensitivity, 0.92 specificity, 0.94 precision, and with an F1-Score of 0.94. Other parkinsonism diagnoses could not be reliably defined so imputation was not performed for diagnoses other than PD. Imputed diagnoses were assumed to be missing at random; features of coded and imputed cases were compared (Supplementary Table 3).

Diagnosis of dementia with Lewy bodies

A diagnosis of dementia with Lewy bodies was accepted when recorded less than one year after a PD diagnosis, but later coding of DLB was considered PD dementia as per diagnostic criteria and counted as PD[10].

Accessibility of protocol, data and the programming code

The approved protocol (23_003515) is available at https://www.cprd.com/approved-studies/incidence-and-prevalence-parkinsons-secular-and-demographic-patterns and data programming codes in Python and R will be made available at publication on Github. Access to the raw data requires a licence application to CPRD.

References

1. Chisholm J. The Read clinical classification. BMJ 1990;300(6732):1092.
2. NHS Digital. SNOMED CT. Available from: https://digital.nhs.uk/services/terminology-and-classifications/snomed-ct (accessed 28th July 2025). 2O23.
3. Noble S, McLennan D, Noble M, et al. The English Indices of Deprivation 2019. Available from: https://assets.publishing.service.gov.uk/media/5d8b364ced915d03709e3cf2/IoD2019_Research_Report.pdf (accessed 29 April 2025).
4. Silcocks PB, Jenner DA, Reza R. Life expectancy as a summary of mortality in a population: statistical considerations and suitability for use by health authorities. J Epidemiol Community Health 2001;55(1):38-43.
5. Public Health England. Fingertips Technical Guidance: Life Expectancy Calculator. Available from: https://fingertips.phe.org.uk/documents/phe%20life%20expectancy%20calculator.xlsm (accessed 20 February 2025).
6. Greenland S. Basic methods for sensitivity analysis of biases. Int J Epidemiol 1996;25(6):1107-1116.
7. Orsini N, Bellocco R, Bottai M, Wolk A, Greenland S. A Tool for Deterministic and Probabilistic Sensitivity Analysis of Epidemiologic Studies. The Stata Journal 2008;8(1):29-48.
8. Tomlinson CL, Stowe R, Patel S, Rick C, Gray R, Clarke CE. Systematic review of levodopa dose equivalency reporting in Parkinson's disease. Movement disorders : official journal of the Movement Disorder Society 2010;25(15):2649-2653.
9. Schade S, Mollenhauer B, Trenkwalder C. Levodopa equivalent dose conversion factors: an updated proposal including opicapone and safinamide. Mov Disord Clin Pract 2020;7(3):343-345.
10. McKeith IG, Boeve BF, Dickson DW, et al. Diagnosis and management of dementia with Lewy bodies: Fourth consensus report of the DLB Consortium. Neurology 2017;89(1):88-100.
