# Supplementary material for: Comparative Analysis of the Incidence, Prevalence, and Survival of 8 Types of Parkinsonism in a Population‐Based Study with 367 Million Person Years of Observation over 21 Years
Source: Mov Disord Clin Pract. 2025 Oct 22;13(4):933–48. doi: 10.1002/mdc3.70368 (PMC13071333; doi:10.1002/mdc3.70368)
Supplement: Supplementary file 2 — TABLE S1. Summary of incidence and prevalence studies of parkinsonism. Original research articles and reviews on the incidence, prevalence and secular trends are summarized for the eight parkinsonian disorders. Most studies investigated individual parkinsonian disorders; several smaller studies combined PD with other parkinsonisms or pooled findings across rarer subtypes. [file MDC3-13-933-s001.pdf]

Supplementary Table 1. Summary of incidence and prevalence studies of parkinsonism

Author	Year(s) of study	Year published	Area; WHO region	Parkinsonism	Incidence / Prevalence
MacDonald et al[1]	01/07/1996	2000	London, UK; European region	PD	Incidence and lifetime prevalence
Foltynie et al[2]	01/09/2000 - 30/06/2003	2004	Cambridge, UK; European region	PD	Incidence
Taylor et al[3]	11/2002 - 04/2004	2006	Aberdeen, UK; European region	PD	Incidence
Caslake et al[4]	2009	2013	Aberdeen, UK; European region	PD, PSP, MSA, DLB, VP, DIP, other	Incidence
Horsfall et al[5]	01/01/1999 - 31/12/2009	2013	UK; European region	PD	Incidence, secular trends
Evans et al[6]	2010	2016	Cambridge, UK; European region	PD	Incidence
Duncan et al[7]	01/06.2009-31/05/2011	2014	Newcastle and Gateshead, UK; European region	PD	Incidence
Okunoye et al[8]	01/01/2006 - 31/12/2016	2022	UK; European region	PD	Incidence, secular trends
Harada et al[9]	1975-1981; Incidence day: 01/04/1980	1983	Yonago, Japan; Western Pacific region	PD	Incidence
Morens et al[10]	1965 - 30/11/1994	1996	O'ahu, Hawaii; Western Pacific region	PD	Incidence
Bower et al[11]	01/01/1976 - 31/12/1990	1999	Olmsted County, Minnesota, USA; region of the Americas	PD, DIP, other, unspecified	Incidence
Baldereschi et al[12]	Baseline screening: 1992 - 1993, Second cross-sectional screening: 1995 - 1996	2000	Italy; European region	PD	Incidence
Morioka et al[13]	1998	2002	Wakayama, Japan; Western Pacific region	PD	Incidence
Van den Eeden et al[14]	1994 - 1995	2003	Northern California, USA; region of the Americas	PD	Incidence
Twelves et al[15]		2003	European, Western Pacific and Eastern Mediterranean regions, and region of the Americas	PD	Incidence
de Lau et al[16]	1993 - 1999	2004	Rotterdam, Netherlands; European Region	PD	Incidence
Tan et al[17]	2001-2002	2007	Singapore; Western Pacific region	PD	Incidence
Alves et al[18]	01/11/2004 - 31/08/2006	2009	Norway; European region	PD	Incidence
Perez et al[19]	1988-2004	2010	Gironde and Dordogne, France; European region	PD, DLB, VP, DIP	Incidence
Linder et al[20]	01/01/2004 - 31/12/2007	2010	Umea, northern Sweden; European region	PD, PSP, MSA-P, unclassifiable	Incidence
Winter et al[21]	01/07/2006 - 31/12/2008	2010	Moscow, Russia; European region	PD, PSP, MSA, CBD, VP, Secondary	Incidence
Savica et al[22]	01/01/1991 - 31/12/2005	2013	Olmsted County, Minnesota, USA; region of the Americas	PD, PSP, MSA, CBS, VP, DIP, unspecified	Incidence

Author	Year(s) of study	Year published	Area; WHO region	Parkinsonism	Incidence / Prevalence
Hirsch et al[23]		2016	European, South-East Asian and Western Pacific regions; region of the Americas	PD	Incidence
Savica et al[24]	1976 - 2005	2016	Olmsted County, Minnesota, USA; region of the Americas	PD	Incidence, secular trends
Darweesh et al[25]	First subcohort: 1990-2000; Second subcohort: 2000 - 2010	2016	Rotterdam, Netherlands; European Region	PD, MSA, DIP, VP	Incidence, secular trends
Canonico et al[26]	1992 - 2018	2022	France; European region	PD	Incidence, secular trends
Brakedal et al[27]	2004 - 2017	2022	Norway; European region	PD	Incidence, secular trends
Dammertz et al[28]	2013 - 2019	2023	Germany; European region	PD	Incidence
Fink et al[29]	2006-2008, 2016-2018	2024	Germany; European region	PD	Incidence
Brewis et al[30]	1961	1966	Carlisle, UK; European region	PD	Prevalence
Sutcliffe et al[31]	Prevalence day: 01/10/1982	1985	Northampton, UK; European region	PD	Prevalence
Mutch et al[32]	Prevalence day: 31/05/1984	1986	Aberdeen, UK; European region	PD	Prevalence
Sutcliffe and Meara[33]	Prevalence day: 01/01/1992; incidence: 1992	1995	Northampton, UK; European region	PD	Incidence and prevalence
Schrag et al[34]	Prevalence day: 01/07/1997	2000	London, UK; European region	PD	Prevalence
Hobson et al[35]	Prevalence day: 06/01/1998	2005	North Wales, UK; European region	PD	Prevalence
Porter et al[36]	Prevalence day: 31/12/2002	2006	North Tyneside, UK; European region	PD	Prevalence
Wickremaratchi et al[37]	2006	2009	Cardiff, UK; European region	PD	Prevalence
Newman et al[38]	01/12/2006-31/08/2007	2009	West Scotland, UK; European region	PD	Prevalence
Walker et al[39]	Prevalence day: 01/11/2003	2010	Northumbria, UK; European region	PD	Prevalence
Parkinson's UK[40]	Incidence: 2011 - 2015; Prevalence: 2015	2017	UK; European region	PD	Incidence and prevalence
Li et al[41]	Prevalence day: 01/01/1983	1985	China; Western Pacific region	PD	Prevalence
Bharucha et al[42]	01/03/1985	1988	Parsi community, India; South-East Asia region	PD	Prevalence
Schoenberg et al[43]	Not stated	1988	Copiah County, USA, region of the Americas, and Igbo-Ora, Nigeria, African region	PD	Prevalence
Granieri et al[44]	Incidence: 1967 - 1987; prevalence day: 31/12/1988	1991	Ferrara, Italy; European region	PD	Incidence and prevalence
Morgante et al[45]	01/11/1987	1992	Sicily; European region	PD, DIP, VP, unspecified	Prevalence
Caradoc-Davies et al[46]	31/07/1990	1992	Dunedin, New Zealand; Western Pacific region	PD	Prevalence

Author	Year(s) of study	Year published	Area; WHO region	Parkinsonism	Incidence / Prevalence
Tison et al[47]	01/1988 - 03/1989	1994	Gironde, France; European region	PD	Prevalence
de Rijk et al[48]	1990 - 1993	1995	The Netherlands; European region	PD, MSA, PSP, VP, DIP	Prevalence
Mayeux et al[49]	1991	1995	Manhattan, New York, USA; region of the Americas	PD	Incidence and prevalence
Tandberg et al[50]	Prevalence day: 01/01/1993	1995	Norway; European region	PD	Prevalence
Kusumi et al[51]	Incidence: 1989 - 1992; Prevalence day: 01/04/1992	1996	Yonago, Japan; Western Pacific region	PD	Incidence and prevalence
Moriwaka et al[52]	Incidence: 1993; Prevalence day: 01/04/1994	1996	Hokkaido Island, Japan; Western Pacific region	PD	Incidence and prevalence
Wang et al[53]	Prevalence day: 01/08/1993	1996	Kinmen, China; Western Pacific region	PD	Prevalence
Fall et al[54]	Prevalence day: 31/08/1989	1996	Sweden; European region	PD	Incidence and prevalence
de Rijk et al[55]	1988-1991	1997	France, Spain, Italy and the Netherlands; European region	PD	Prevalence
Melcon et al[56]	01/01/1991	1997	Argentina; region of the Americas	PD, DIP, VP	Prevalence
Wermuth et al[57]	Prevalence day: 01/07/1995	1997	Faroe Islands, Denmark; European region	PD, PSP, MSA, VP	Prevalence
Chiò et al[58]	20/10/1991	1998	Cossato, Italy; European region	PD	Prevalence
Errea et al[59]	Prevalence day: 31/12/1992	1999	Spain; European region	PD, MSA, VP, DIP, unspecified	Prevalence
Kuopio et al[60]	Prevalence day: 01/01/1992	1999	Finland; European region	PD	Incidence, prevalence, secular trends
Wermuth et al[61]	Prevalence day: 01/01/1998	2000	Island of Als, Denmark; European region	PD	Prevalence
Chen et al[62]	Prevalence day: 01/01/1993	2001	Ilan, Taiwan; Western Pacific region	PD	Incidence and prevalence
Milanov et al[63]	Prevalence day: 31/12/1999	2001	Bulgaria; European region	PD	Prevalence
Wermuth et al[64]	Prevalence day: 01/01/2000	2002	Greenland; European region	PD	Prevalence
Kis et al[65]	1998	2002	South Tyrol, Italy; European region	PD	Prevalence
Claveria et al[66]	Prevalence day: 31/12/1994	2002	Cantalejo, Spain; European region	PD	Prevalence
Anca et al[67]	1998	2002	Kibbutzim, Israel; European region	PD	Prevalence
Taba and Asser[68]	Prevalence day: 01/01/1996	2002	Tartu, Estonia; European region	PD	Prevalence
Kimura et al[69]	2000	2002	Yamagata prefecture, Japan; Western Pacific region	PD	Prevalence
Nicoletti et al[70]	Prevalence day: 01/11/1994	2003	Cordillera province, Bolivia; region of the Americas	PD	Prevalence
Benito-Leon et al[71]	Prevalence day: 01/05/1994	2003	Spain; European region	PD, DIP, VP	Prevalence

Author	Year(s) of study	Year published	Area; WHO region	Parkinsonism	Incidence / Prevalence
Zhang et al[72]	Prevalence day: 31/12/1997	2003	Beijing, China; Western Pacific region	PD	Prevalence
Bergareche et al[73]	1996	2004	Bidasoa, Spain; European region	PD	Prevalence
Tan et al[74]	Prevalence day: 02/04/2001	2004	Singapore; Western Pacific region	PD	Prevalence
Sanchez et al[75]	1997	2004	Colombia; region of the Americas	PD	Prevalence
Totaro et al[76]	Prevalence day: 31/12/2001	2005	Italy; European region	PD	Prevalence
Chan et al[77]	08/2002 - 07/2003	2005	Sydney, Australia; Western Pacific region	PD	Prevalence
Zhang et al[78]	1997 - 1998	2005	Beijing, Xian and Shanghai, China; Western Pacific region	PD	Prevalence
Zhang et al[79]	08/11/1999 - 30/04/2000	2005	Linxian, China; Western Pacific region	PD	Prevalence
Von Campenhausen et al[80]		2005	UK, France, Italy, Portugal, Spain, Germany, the Netherlands, Sweden, Austria, Czech Republic; European region	PD	Incidence and prevalence
Okubadejo et al[81]		2006	Algeria, Ethiopia, Ghana, Kenya, Libya, Nigeria, Senegal, South Africa, Tanzania, Togo, Tunisia, Uganda, Zimbabwe; African region	PD	Incidence and prevalence
Barbosa et al[82]	2001	2006	Bambui, Brazil; region of the Americas	PD, DIP, VP	Prevalence
Mehta et al[83]	Prevalence: 1997 - 1999; incidence: 1992-1994, 1997-1999, 2002-2004	2007	Sydney, Australia; Western Pacific region	PD	Incidence and prevalence
Wermuth et al[84]	01/07/2005	2008	Faroe Islands, Denmark; European region	PD, PSP, MSA	Incidence and prevalence
Dotchin et al[85]	Prevalence day: 01/03/2006	2008	Hai, Tanzania; African region	PD	Prevalence
Morgante et al[86]	Prevalence day: 01/01/2001	2008	Aeolian Archipelago, Sicily; European region	PD	Prevalence
Alrefai et al[87]	03/2007 - 04/2008	2009	Jordan; Eastern Mediterranean region	PD	Prevalence
Yamawaki et al[88]	Yonago: 01/04/2004; Daisen: 01/04/2003	2009	Yonago city and Daisen town, Japan; Western Pacific region	PD	Incidence, prevalence, secular trends
Chen et al[89]	Prevalence day: 01/01/2001	2009	Keelung city, Taiwan; Western Pacific region	PD	Prevalence
Muangpaisan et al[90]		2009	China, Taiwan, Japan, Singapore, Israel, Saudi Arabia; Western Pacific, European and Eastern Mediterranean regions	PD	Incidence and prevalence
Lix et al[91]	Incidence: 1987/1988 and 2006/2007 fiscal years	2010	Manitoba, Canada; region of the Americas	PD	Incidence, prevalence, secular trends

Author	Year(s) of study	Year published	Area; WHO region	Parkinsonism	Incidence / Prevalence
Masalha et al[92]	2005	2010	Israel; European region	PD	Prevalence
Das et al[93]	Incidence: 2003 - 2008; prevalence day: 31/12/2007	2010	India; South-East Asia region	PD	Incidence and prevalence
Wright Willis et al[94]	1995; 2000-2005	2010	USA; region of the Americas	PD	Incidence, prevalence, secular trends
Osaki et al[95]	Prevalence day: 01/11/2007	2011	Koban district, Japan; Western Pacific region	PD, PSP, MSA, CBD	Prevalence
Seijo-Martinez et al[96]	Prevalence day: 31/12/2003	2011	Arosa Island, Spain; European region	PD	Prevalence
Khedr et al[97]	Prevalence day: 31/12/2010	2012	Assiut governorate, Egypt; Eastern Mediterranean region	PD, DIP, VP	Incidence and prevalence
Gordon et al[98]	2002 - 2009	2012	Alaska, USA; region of the Americas	PD	Incidence, prevalence, secular trends
Gordon et al[99]	Period prevalence: 1995-1999, 2000-2004, and 2005-2009	2013	Alaska, USA; region of the Americas	PD	Prevalence, secular trends
El-Tallawy et al[100]	01/06/2005-31/05/2009	2013	Al Kharga district, Egypt; Eastern Mediterranean region	PD, VP, unspecified	Prevalence
Blanckenberg et al[101]	1982-2006 (original studies published)	2013	Ethiopia, Nigeria, Togo, Tanzania; African region	PD	Prevalence
Pringsheim et al[102]	1985-2010	2014	European, Western Pacific, South-East Asian, Eastern Mediterranean and African regions, and region of the Americas	PD	Prevalence
Blin et al[103]	2005 - 2010	2015	France; European region	PD	Incidence and prevalence
Gordon et al[104]	Incidence: 2001 - 2011; Prevalence day: 01/07/2006	2015	Alaska, USA; region of the Americas	PD	Incidence and prevalence
Khedr et al[105]	Prevalence day: 31/08/2013	2015	Qena governorate, Egypt; Eastern Mediterranean region	PD, DIP, VP, CBD	Incidence and prevalence
Yang et al[106]	11/2010 - 12/2010	2015	China; Western Pacific region	PD	Prevalence
Zou et al[107]		2015	China, Taiwan and Hong Kong; Western Pacific region	PD	Incidence and prevalence
Liu et al[108]	2002 - 2009	2016	Taiwan; Western Pacific region	PD	Incidence, prevalence, secular trends
GBD 2016[109]	1990 - 2016	2016	All WHO regions	PD	Prevalence and years lived with disability
Muangpaisan et al[110]	Not stated	2017	Bangkok, Thailand; South-East Asia region	PD	Prevalence
Abbas et al[111]		2018	European, Western Pacific, South-East Asia regions and the region of the Americas	PD	Incidence and prevalence
Williams et al[112]		2018	Nigeria, Ethiopia, Togo, Tanzania; African region	PD	Prevalence

Author	Year(s) of study	Year published	Area; WHO region	Parkinsonism	Incidence / Prevalence
Fleury et al[113]	Incidence: 01/01/2003 - 31/12/2012; Prevalence day: 01/01/2013	2018	Canton of Geneva, Switzerland; European region	PD, PSP, MSA, CBS, DLB, DIP	Incidence and prevalence
Kadastik-Eerme et al[114]	Prevalence day: 01/10/2013	2018	Tartu, Estonia; European region	PD	Prevalence, secular trends
Valent et al[115]	2016	2018	Friuli Venezia Giulia, Italy; European region	PD	Incidence and prevalence
Szatmári et al[116]	2010 - 2012	2019	Hungary; European region	PD	Incidence and prevalence
Park et al[117]	01/2010 - 12/2015	2019	South Korea; South-East Asia region	PD	Incidence, prevalence, secular trends
Han et al[118]	2012 - 2015	2019	Korea; South-East Asia region	PD, DIP	Incidence, prevalence and secular trends
GBD 2021[119]	1990 - 2021	Online (https://vizhub.healthdata.org/gbd-results/)	All WHO regions	PD	Incidence, prevalence, secular trends
Qi et al[120]	06/2015 - 12/2015	2021	China; Western Pacific region	PD	Prevalence
Song et al[121]	03 - 10/2019	2022	China; Western Pacific region	PD	Prevalence
Cicero et al[122]	2017	2022	Sicily, Italy; European region	PD	Prevalence
Llibre-Guerra et al[123]		2022	Peru, Cuba, Dominican Republic, Puerto Rico, Venezuela and Mexico; region of the Americas	PD	Prevalence
Varden et al[124]		2024	UK; European region	PD	Prevalence
Zhu et al[125]	Until 01/11/2023	2024	All WHO regions	PD	Prevalence, secular trends
Kim et al[126]	2022	2024	13 south American countries; region of the Americas	PD	Incidence and prevalence
Bower et al[127]	01/01/1976 - 31/12/1990	1997	Olmsted County, Minnesota, USA; region of the Americas	PSP and MSA	Incidence
Schrag et al[128]	Prevalence day: 01/07/1997	1999	London, UK; European region	PSP and MSA	Prevalence
Kawashima et al[129]	Prevalence day: 01/04/1999	2004	Yonago city, Japan; Western Pacific region	PSP	Prevalence
Wada-Isoe et al[130]	Prevalence day: 01/03/2008	2009	Ama-Cho, Japan; Western Pacific region	PSP, DLB	Prevalence
Vann Jones et al[131]		2014	European, Western Pacific, and South-East Asia regions and the region of the Americas	DLB	Incidence and prevalence
Hogan et al[132]		2016	UK, France, Italy, Spain, Finland, Turkey, US, Australia, Brazil, Cuba, Japan, Korea, Sri Lanka. European, Western Pacific and South-East Asia regions, and region of the Americas	DLB	Incidence and prevalence

Author	Year(s) of study	Year published	Area; WHO region	Parkinsonism	Incidence / Prevalence
Takigawa et al[133]	Prevalence day: 01/10/2010	2016	Yonago city, Japan; Western Pacific region	PSP	Prevalence, secular trends
Savica et al[134]	1976-2005	2017	Olmsted County, Minnesota, USA; region of the Americas	DIP	Incidence, secular trends
Stang et al[135]	01/01/1991 - 31/12/2005	2020	Olmsted County, Minnesota, USA; region of the Americas	PSP, CBS	Incidence, secular trends
Swallow et al[136]		2022	UK, Switzerland, Faroe Islands, the Netherlands, Australia, Japan, China, Singapore, Egypt, USA, Brazil. European, Western Pacific and Eastern Mediterranean regions, and region of the Americas	PSP, CBS	Prevalence, secular trends
Swallow et al[137]	Prevalence day: 31/12/2018	2022	Scotland; European region	PSP, CBS	Prevalence
Lyons et al[138]	Inception until 13/07/21	2023	European, Western Pacific, and Eastern Mediterranean regions, and region of the Americas	PSP, CBS	Incidence and prevalence
Logroscino et al[139]	01/06/2018 - 31/05/2019	2023	UK, Germany, Italy, Netherlands, Finland, Sweden, Bulgaria, Serbia; European region	PSP, CBS	Incidence
Kaplan et al[140]	1995 - 2022	2024	UK, France, Italy, Spain, Germany, Netherlands, Switzerland, Norway, Sweden, Iceland, USA, Japan, Brazil. European and Western Pacific regions, and region of the Americas	MSA	Prevalence

Author	Study design	Population size	Age	Crude incidence rate per 100,000	Adjusted / standardised incidence rate per 100,000
MacDonald et al[1]	Prospective community-based study using healthcare records.	100,230	All ages	.	19.0 (12.0, 27.0)
Foltynie et al[2]	Population-based study using primary and secondary care healthcare records, followed by clinical assessment for case verification.	708,715	All ages	***PD:*** 13.6 (11.8, 15.6)	***PD:*** 10.8 (9.4, 12.4)
Taylor et al[3]	Prospective community-based study using primary and secondary care hospital records, followed by clinical assessment.	148,600	All ages	22.4 (16.6, 29.6)	22.1
Caslake et al[4]	Population-based studying involving primary and secondary healthcare records, followed by clinical assessment.	317,357 people; 1,176,552 person years	All ages	***PD:*** 17.9 (15.5, 20.4); ***PSP:*** 1.7; ***MSA:*** 1.4 ***DLB:*** 3.4; ***VP:*** 3.3; ***DIP:*** 0.3; ***Other:*** 0.9	Adjusted PD incidence (to 1990 Scottish population) from a meta-analysis of 12 studies (including Caslake study): 14.6 (12.2, 17.3) per 100,000
Horsfall et al[5]	Population-based study involving THIN electronic health records.	Denominator not specified, but 1.6 million active patients ≥ 50 years in THIN	≥ 50 years	84 (82, 85) using PD Read codes only; 118 (116, 120) with broadest case definition	
Evans et al[6]	Prospective community-based study involving primary and secondary healthcare records, followed by clinical assessment.	600,600	All ages	13.0 (10.9, 15.1)	15.8 (13.7, 18.0)
Duncan et al[7]	Prospective longitudinal study involving healthcare records, followed by clinical examination.	488,576	All ages	15.9 (13.4, 18.4)	12.0 (10.1, 14.0)
Okunoye et al[8]	Population-based study involving THIN electronic health records.	3.7 million active patients (out of 12 million in total) in THIN	≥ 50y	***PD Read codes + at least 2 anti-park medications:*** 57 (56, 58); ***PD Read codes:*** 70 (68, 71); ***Broadest case definition:*** 140 (138, 141)	***Broadest case definition:*** 143.70
Harada et al[9]	Record-based survey of primary and secondary care data and national health insurance records.	125,291	All ages	***Overall:*** 80.6; ***≥ 50 years:*** 283	

Author	Study design	Population size	Age	Crude incidence rate per 100,000	Adjusted / standardised incidence rate per 100,000
Morens et al[10]	Cohort study following 8006 American men of Japanese or Okinawan ancestry, born between 1900 and 1919 and residing in O'ahu, Hawaii in 1965. Incident cases identified through review of healthcare records and rescreening assessment.	8,006	Cases born between 1900 - 1919		***All ages:*** 11.1; ***50+:*** 45.2
Bower et al[11]	Population-based study using medical records linkage system of the Rochester Epidemiology Project, followed by clinical assessment.	1,424,474 person years	All ages	***PD:*** 10.8 (154) ***DIP:*** 5.1 (72) ***Other:*** 1.8 (26) ***Unspecified:*** 4.3 (61)	
Baldereschi et al[12]	Population-based cohort study involving door-to-door screening.	5,463 eligible cases; 3,084 in final cohort	65 - 84 years	346 (241, 450)	326 (224, 427)
Morioka et al[13]	Cross-sectional record-based study involving a mailed questionnaire on incident cases to 873 medical facilities (hospitals and clinics).	1,372,781	≥ 40 years	***Annual incidence rate:*** 16.9 (14.5, 19.3)	***Total adjusted incidence:*** 10.5 (9.3, 11.7)
Van den Eeden et al[14]	Study involving review of Kaiser Permanente Medical Care Program of Northern California healthcare records.	4,776,038 person years	All ages	12.3	13.4 (11.4, 15.5)
Twelves et al[15]	Systematic review of PD incidence studies using MEDLINE (1966-12/2001) and EMBASE (1980-12/2001). 25 studies included but only 5 sufficiently similar for comparison.	.	All ages	***Overall rate from similar incidence studies (4/5):*** 16-19	.
de Lau et al[16]	Prospective population-based study involving door-to-door screening.	Cohort at risk: 6,566; 39,878 person years	≥ 55 years	***PD:*** 170 (140, 220)	.

Author	Study design	Population size	Age	Crude incidence rate per 100,000	Adjusted / standardised incidence rate per 100,000
Tan et al[17]	Population-based cohort study involving case identification in a parkinsonism-free cohort by phone interview, review of primary and / or secondary medical records, and a database merge for additional secondary and tertiary care records. Diagnostic verification performed by a Movement Disorders specialist through full case record review.	14,833 individuals; 31,426 total person years	≥ 50 years	33 (18, 68)	32 (17, 68)
Alves et al[18]	Prospective population-based longitudinal incident cohort study involving review of primary and secondary care records, followed by regular clinical assessment.	1,052,075	All ages	13.7 (12.1, 15.5)	12.6 (11.1, 14.2)
Perez et al[19]	Population-based prospective elderly cohort study conducted over a 15-year period, involving a screening questionnaire, review of primary and secondary care case records, followed by clinical assessment for a proportion of cases. All cases consensually classified by diagnostic consensus board.	25,820 person years	> 65 years	***PD:*** 263; ***DLB:*** 112; ***VP:*** 89.1; ***DIP:*** 58.1	
Linder et al[20]	Prospective population-based incidence cohort study, involving review of records followed by clinical assessment.	1,419,50 people; 567,800 person years of observation	All ages	***PD:*** 19.7 (16.1, 23.3); ***MSA-P:*** 2.1 (1.1, 3.7); ***PSP:*** 1.1 (0.4, 2.4); ***Idiopathic parkinsonism:*** 24.3 (20.2, 28.4)	***PD:*** 22.5 (18.3, 26.7); ***MSA-P:*** 2.4 (1.4, 4.2); ***PSP:*** 1.2 (0.4, 2.6); ***Idiopathic parkinsonism:*** 27.5 (22.9, 32.1)
Winter et al[21]	Prospective population-based cohort study. Cases confirmed by examination.	1,237,900 people; 3,094,750 person years	All ages	***PD:*** 10.0 (8.9, 11.1); ***CBD:*** 0.0 (0.0, 0.2); ***VP:*** 2.6; ***Secondary:*** 0.3	***PD:*** 9.0 (8.0, 10.2); ***PSP:*** 0.1 (0.1, 0.2); ***CBD:*** 0.0 (0.0, 0.1); ***MSA:*** 0.1 (0.0, 0.2)

Author	Study design	Population size	Age	Crude incidence rate per 100,000	Adjusted / standardised incidence rate per 100,000
Savica et al[22]	Population-based study of the incidence of synucleinopathies and tauopathies using medical records from the Rochester Epidemiology Project, followed by complete record review by a Movement Disorders specialist.	1,852,762 person years	All ages	***PD:*** 14.2; ***PSP:*** 0.9; ***MSA:*** 0.8; ***CBS:*** 0.2 ***Tauopathy (PSP + CBS):*** 1.1. ***Synucleinopathy:*** 21.0; ***DIP:*** 1.9; ***VP:*** 0.6; ***Unspecified:*** 4.5	***PD:*** 16.5
Hirsch et al[23]	Systematic review and meta-analysis of PD incidence studies from 2001 - 2014.	.	≥ 40y	***Overall PD incidence in females ≥ 40y:*** 37.6 (26.2, 53.8); ***Overall PD incidence in males ≥ 40y:*** 61.2 (43.6, 86.0)	.
Savica et al[24]	Population-based cohort study using medical records linkage system of the Rochester Epidemiology Project, followed by clinical assessment.	***1976 - 1985:*** 941,262; ***1986 - 1995:*** 1,093,682; ***1996 - 2005:*** 1,283,875; ***1976 - 2005:*** 3318819	All ages	***1976 - 1985:*** 10.1 (95); ***1986 - 1995:*** 12.1 (132); ***1996 - 2005*** : 18.5 (237); ***1976 - 2005 (all years):*** 14.0 (464)	***1976 - 1985:*** 13.8; ***1986 - 1995:*** 15.4; ***1996 - 2005:*** 20.7; ***1976 - 2005 (all years):*** 17.2.
Darweesh et al[25]	Population-based cohort study, with comparison of data from the first 2 subcohorts (1990-2000 vs. 2000-2010).	***PD 1990 - 2000:*** 55,920 person years at risk; ***PD 2000-2010:*** 22,224 person years at risk<br><br>***MSA, DIP, VP 1990 - 2000:*** 57,052 person years at risk; ***MSA, DIP, VP 2000-2010:*** 57,052 person years at risk	≥ 55 years	***1990-2000 PD:*** 150.0 (120.0, 186.0); ***2000-2010 PD:*** 45.0 (22.0, 83.0) ***1990-2000 MSA:*** 5 (1, 15) ***2000-2010 MSA:*** 4 (0, 25) ***1990-2000 DIP:*** 33 (20, 52) ***2000-2010 DIP:*** 4 (0, 25) ***1990-2000 VP:*** 12 (5, 25); ***2000-2010 VP:*** 0 (0, 17) ***1990-2000 unspecified:*** 100 (76, 129) ***2000-2010 unspecified:*** 27 (10, 59)	
Canonico et al[26]	Prospective cohort study of French women, born between 1925 - 1950, affiliated with a French national health insurance plan.	98,069	40 - 65 years at baseline	***Overall 1992 - 2018:*** 49.4 (46.6, 52.2); ***1992 - 1997:*** 11.7 (9.2, 14.8); ***1998 - 2018:*** 61.2 (57.7, 64.9)	.

Author	Study design	Population size	Age	Crude incidence rate per 100,000	Adjusted / standardised incidence rate per 100,000
Brakedal et al[27]	Population-based study of the Norwegian Prescription Database, involving drug prescription records.	4.6–5.3 million for the study period	≥ 30y	***Males*** **:** 29.6; ***Females*** **:** 23.1	
Dammertz et al[28]	Population-based cohort study involving national outpatient claims and drug prescription records from German Statutory Health Insurance.	30,575,726	≥ 50 years		***2013:*** 137; ***2019:*** 106
Fink et al[29]	Population-based study of 2 randomly drawn samples of individuals ≥ 50y from 2004-2009 and 2014-2019.	446796; ***Males:*** 261,926 person years; ***Females:*** 358,884 person years	≥ 50y	***Males 2006 - 2008:*** 224 (207, 243); ***Females 2006 - 2008:*** 197 (183, 212) ***Males 2016 - 2018:*** 193 (178, 210); ***Females 2016 - 2018:*** 165 (153, 179)	.
Brewis et al[30]	Community-based study involving primary and secondary care healthcare records and a survey of 2388 households.	71,101	All ages	.	
Sutcliffe et al[31]	Cross-sectional study involving primary and secondary care healthcare records, followed by examination in uncertain cases.	208,499	All ages	.	.
Mutch et al[32]	Cross-sectional study involving primary, secondary care and medication records, followed by clinical assessment.	151,616	All ages	.	.
Sutcliffe and Meara[33]	Cross-sectional study involving healthcare records.	302,500	All ages	12	.
Schrag et al[34]	Cross-sectional study involving screening of primary care healthcare records, followed by clinical assessment.	121,608	All ages	.	.
Hobson et al[35]	Cross-sectional study involving primary care prescription data and secondary care records.	77,338	All ages	.	.
Porter et al[36]	Cross-sectional study involving primary and secondary care records and prescription data, followed by clinical assessment.	108,597	All ages	.	.

Author	Study design	Population size	Age	Crude incidence rate per 100,000	Adjusted / standardised incidence rate per 100,000
Wickremaratchi et al[37]	Community-based cross-sectional study involving primary and secondary care records and prescription data, followed by clinical assessment for around one third of cases. Meta-analysis of previous and current UK prevalence studies performed.	292,637	All ages	.	.
Newman et al[38]	Cross-sectional prevalence study involving primary care prescription database searches and full case record review.	511,927	All ages	.	.
Walker et al[39]	Cross-sectional study involving primary and secondary care records and prescription data, followed by clinical assessment.	59,613	All ages	.	.
Parkinson's UK[40]	Population-based study involving CPRD electronic health records.	CPRD prevalence population: 2,551,470	≥ 20 years	***All ages:*** 26.6 ***≥18:*** 33.7 ***≥20:*** 34.8	.
Li et al[41]	Population-based study involving door-to-door screening.	63,195	All ages	.	.
Bharucha et al[42]	Cross-sectional 2-phase study involving door-to-door screening with a questionnaire, followed by clinical examination by a Neurologist for cases that screened positive.	14,010	All ages		
Schoenberg et al[43]	Population-based study involving door-to-door screening with a questionnaire, followed by clinical examination for cases that screened positive, in Copiah County and Igbo-Ora.	***Copiah County population:*** 24,000 (8,925 eligible > 39); ***Igbo-Ora, Nigeria population*** : 20,000 (3,412 eligible)	≥ 40 years	.	.
Granieri et al[44]	Cross-sectional study involving administrative records.	Incidence: 187,381; Prevalence: 176,621	All ages	10.0 (9.1, 11.1)	8.6
Morgante et al[45]	Cross-sectional study involving door-to-door screening, followed by neurological examination of cases that screened positive.	24,496	All ages	.	.

Author	Study design	Population size	Age	Crude incidence rate per 100,000	Adjusted / standardised incidence rate per 100,000
Caradoc-Davies et al[46]	Cross-sectional study involving primary and secondary care records and medication data, followed by clinical assessment.	105,075	All ages	.	.
Tison et al[47]	Population-based study involving door-to-door screening.	3149 residents ≥ 65 years (2792 in community + 357 institutionalised)	≥ 65 years	.	.
de Rijk et al[48]	Population-based cohort study involving 2-phase door-to-door screening, followed by examination by a Neurologist if positive on screening.	6,969	≥ 55 years		.
Mayeux et al[49]	Cross-sectional study involving administrative records.	Prevalence: 213302; Incidence: 639,294 person years	All ages	13.0 (10.2, 15.8)	.
Tandberg et al[50]	Cross-sectional study involving review of healthcare records, followed by interview and examination by a Neurologist.	220,858	All ages		
Kusumi et al[51]	Cross-sectional study involving review of medical records and questionnaires sent to clinics and hospitals.	132,315	All ages	***Incidence from 1989 - 1992:*** 15.0	.
Moriwaka et al[52]	Cross-sectional study involving secondary care records.	**Hokkaido Island:** 5,643,647; **Iwamizawa City:** 80,417	All ages	***Hokkaido:*** 8.7	
Wang et al[53]	Community-based study involving single-phase door-to-door screening.	3,915	≥ 50 years		
Fall et al[54]	Cross-sectional study involving healthcare records.	147,777	All ages	11	7.9
de Rijk et al[55]	Cross-sectional study involving door-to-door screening.	14,636 ≥ 65 years	≥ 65 years	.	.
Melcon et al[56]	Cross-sectional 2-phase study involving door-to-door screening questionnaire, followed by examination by a Neurologist for cases that screened positive.	7,765	≥ 40 years		

Author	Study design	Population size	Age	Crude incidence rate per 100,000	Adjusted / standardised incidence rate per 100,000
Wermuth et al[57]	Cross-sectional study involving medication, nursing home, primary and secondary care records. Identified cases were interviewed and neurologically examined.	***Overall:*** 43,709; ***≥ 50 years:*** 12,380	All ages		
Chiò et al[58]	Cross-sectional study involving primary and secondary care records and levodopa prescription data, followed by clinical assessment.	61,830	All ages	.	.
Errea et al[59]	Cross-sectional study involving healthcare records.	60,724	All ages	.	.
Kuopio et al[60]	Cross-sectional study involving review of data from the Social Insurance Institute of Finland and hospital medical records, followed by examination of identified cases.	196,864	All ages	***1971:*** 14.8 ***1992:*** 17.2	***1971:*** 15.7 (11.6, 19.8); ***1992:*** 14.9 (9.9, 20.0)
Wermuth et al[61]	Cross-sectional study involving medication, nursing home, primary and secondary care records. Identified cases completed a questionnaire, were interviewed and neurologically examined.	56,839	All ages	.	.
Chen et al[62]	Cross-sectional study involving two-phase door-to-door screening with a questionnaire, followed by examination by Neurologists.	***Total population:*** 75,579; ***Population ≥ 40 years:*** 13,466; ***Total participants ≥40 years:*** 10,058	≥ 40 years	30.1	***All ages:*** 10.4; ***40+:*** 28.7
Milanov et al[63]	Cross-sectional study involving review of secondary care records, followed by clinical examination in 2 small regions in Bulgaria.	119,910	All ages	.	.
Wermuth et al[64]	Collation of medication, nursing home, primary and secondary care records.	49,369	All ages	.	.

Author	Study design	Population size	Age	Crude incidence rate per 100,000	Adjusted / standardised incidence rate per 100,000
Kis et al[65]	Population-based prospective cross-sectional study involving door-to-door screening. Questionnaire mailed to a random sample of 750 cases aged 60-85 years, followed by clinical examination. Diagnoses verified by a Movement Disorder specialist.	750	60-85 years	.	.
Claveria et al[66]	Population-based longitudinal study involving door-to-door screening with minimum follow-up of 3 years.	1,579	≥ 40 years	.	
Anca et al[67]	Population-based cross-sectional study involving door-to-door screening.	***All ages:*** 76,767; ***Age > 40y:*** 53,104; ***Age > 60y:*** 14,646	All ages		
Taba and Asser[68]	Community-based cross-sectional study involving review of healthcare records, followed by clinical examination.	153,240	All ages	.	.
Kimura et al[69]	Secondary care record-based survey in clinics and hospitals involving a 2-step questionnaire.	1,256,958	All ages	.	.
Nicoletti et al[70]	Cross-sectional study involving door-to-door screening, followed by neurological examination of cases that screened positive.	1,780	≥ 40 years	.	.
Benito-Leon et al[71]	Population-based cross-sectional study with door-to-door screening.	5,278	≥ 65 years		
Zhang et al[72]	Cross-sectional study involving door-to-door screening with a questionnaire, followed by neurological examination by a Neurologist.	5,743	≥ 55 years		
Bergareche et al[73]	Population-based three-phase study, with door-to-door screening, followed by assessment by a Neurologist for cases that screened positive, and then 3-monthly examinations for 3 years to verify the diagnosis.	1,540	≥ 65 years	.	.

Author	Study design	Population size	Age	Crude incidence rate per 100,000	Adjusted / standardised incidence rate per 100,000
Tan et al[74]	Cross-sectional study involving 3-phase door-to-door screening of Chinese, Maly and Indian individuals in Singapore. Cases identified by questionnaire and examination, with diagnostic confirmation by Movement Disorder specialists.	14,906	≥ 50 years	.	.
Sanchez et al[75]	Cross-sectional study using a capture-recapture method to identify PD cases attending two secondary care neurological institutions.	1,442,997	All ages		
Totaro et al[76]	Cros-sectional study involving healthcare records.	297,424	All ages		
Chan et al[77]	Cross-sectional study involving door-to-door screening.	1028 (pooled results of 2 studies in Bankstown and Randwick)	≥ 55 years	.	.
Zhang et al[78]	Cross-sectional study involving stratified, multistage door-to-door screening in 79 rural and 58 urban communities in China with a questionnaire, followed by neurological examination by a Neurologist.	29,454	≥ 55 years		
Zhang et al[79]	Cohort study in rural Linxian, China, involving collection of demographic and general medical information, followed by a screening questionnaire and examination. Further examination for cases that screened positive by a Neurologist to verify diagnosis.	16,488	≥ 50 years	.	.
Von Campenhausen et al[80]	Systematic review of PD incidence and prevalence studies in European countries using 8 different databases. Significant methodological heterogeneity in included studies limited comparison.	.	All ages	***Annual PD incidence:*** 5 - 346; ***High quality studies only:*** 11-19	

Author	Study design	Population size	Age	Crude incidence rate per 100,000	Adjusted / standardised incidence rate per 100,000
Okubadejo et al[81]	Systematic review of African incidence (n = 1) and prevalence studies (n = 7) published between 1944 and December 2004.	***Nigeria:*** 20,000; ***Libya:*** 518,745; ***Tunisia:*** 34,874; ***Ethiopia:*** 60,820; ***Togo 1989:*** 19,241; ***Togo 1995:*** 4,182	All ages	***Benghazi, northeast Libya (1982 - 1984):*** 4.5	.
Barbosa et al[82]	Population-based cohort study, involving a door-to-door screening questionnaire and examination of cases that screened positive by a Neurologist.	1,186	≥ 64 years	.	.
Mehta et al[83]	Population-based cohort study assessing cross-sectional prevalence and 10 year incidence with door-to-door screening and 5-yearly examinations.	Prevalence: 3509; incidence: 2545	≥ 49 years	81 (44, 117)	
Wermuth et al[84]	Cross-sectional study involving healthcare records.	48,371	All ages	***PD:*** 21.1 ***PD + atypical parkinsonism:*** 22.9	
Dotchin et al[85]	Cross-sectional study involving door-to-door screening.	160,456	All ages		
Morgante et al[86]	Cross-sectional study involving review of medication, primary and secondary care records, and a screening survey mailed only to residents ≥ 40 years. Cases that screened positive were examined.	13,431	All ages	.	.
Alrefai et al[87]	Cross-sectional study involving review of Neurology clinic hospital records, followed by clinical examination.	173,450	≥ 30 years		
Yamawaki et al[88]	Service-based study in Yonago city and a door-to-door study in Daisen, involving screening questionnaire followed by neurological examination for cases that screened positive.	***Japan:*** 127,687 ***Yonago:*** 140,911; ***Daisen:*** 6,849	All ages	***Yonago 2004:*** 18.4 (11.3, 25.5) ***Yonago 1980:*** 10.2 (4.6, 15.8)	***Yonago 2004:*** 10.3 (4.7, 15.9); ***Yonago 1992:*** 9.8 (4.3, 15.3);

Author	Study design	Population size	Age	Crude incidence rate per 100,000	Adjusted / standardised incidence rate per 100,000
Chen et al[89]	Cross-sectional study involving door-to-door screening.	149,949 eligible residents; 11332 screened	≥ 40 years	.	.
Muangpaisan et al[90]	Systematic review and meta-analysis of studies from 1965 - 10/2008 evaluating PD incidence and prevalence in Asia using MEDLINE and EMBASE. 21 studies included.	.	All ages		***Door-to-door studies:*** 8.7; ***Record-based studies:*** 6.7-8.3
Lix et al[91]	Population-based study involving administrative healthcare data.	1.2 million	≥ 25 years	.	***2006/2007 males:*** 102 (88.5, 118); ***2006/2007 females:*** 63.4 (54.9, 73.1)
Masalha et al[92]	Cross-sectional study involving review of medication records using drug-tracer methodology, followed by examination by a Neurologist for diagnostic verification.	114,998	All ages	.	.
Das et al[93]	Cohort study involving door-to-door screening.	100,802	All ages	4.56 (2.87, 7.51)	5.71 (3.59, 9.40)
Wright Willis et al[94]	Serial cross-sectional study of US Medicare beneficiaries for 1995, and 2000-2005, including an analysis by ethnicity.	29,350,834	≥ 65 years	***Mean annual incidence from 2002-2005:*** 446 (± 1.9)	.
Osaki et al[95]	Cross-sectional study involving review of healthcare records, followed by clinical assessment.	66,465	All ages	.	.
Seijo-Martinez et al[96]	Cross-sectional study involving 1-stage door-to-door screening.	753	≥ 65 years	.	.
Khedr et al[97]	Community-based cross-sectional study involving door-to-door screening within 7 randomly sampled districts in Egypt	5,920	All ages	PD: 84 (10, 158)	.
Gordon et al[98]	Cross-sectional study involving primary and secondary care data from the Indian Health Service.	1,816,640	All ages	16.1	29
Gordon et al[99]	Cross-sectional study involving healthcare records from the Shiprock Service Unit Indian Health Service database.	***2005-2009:*** 61,853; ***2000-2004:*** 59,229; ***1995-1999:*** 54,584;	All ages	.	.

Author	Study design	Population size	Age	Crude incidence rate per 100,000	Adjusted / standardised incidence rate per 100,000
El-Tallawy et al[100]	Cross-sectional study involving door-to-door screening.	15,482	≥ 40 years	.	.
Blanckenberg et al[101]	Review of published prevalence and genetic studies on Black sub-Saharan African populations up until July 2013.	Range: 4182 (two districts in northern Tanzania) - 161,071 (Tanzania)	All ages	.	.
Pringsheim et al[102]	Systematic review and meta-analysis of 47 PD epidemiological studies from 1985 - 2010 using MEDLINE and EMBASE.	.	All ages	.	.
Blin et al[103]	Population-based cross-sectional study involving insurance and national hospital-discharge records.	2005 - 2010: 384,762,297 person years	≥ 18 years	.	***Specific definition 2005 - 2010:*** 36.0 (36.0, 36.0) ***Sensitive definition 2005 - 2010:*** 49.0 (48.0, 49.0)
Gordon et al[104]	Cross-sectional study involving inpatient and outpatient data from the Indian Health Service for American Indian individuals on the Navajo Reservation. Chart review of diagnosis and coding by Movement Disorder specialist to verify accuracy.	Prevalence: 217,158 Incidence: 2,333,313 (over the 10 years)	All ages	***Annual crude incidence:*** 22.5 (20.5, 24.4); ***≥ 65y:*** 232 (210, 254); ***≥ 80y:*** 302	***All ages:*** 35.9 (32.8, 39.0); ***≥ 65y:*** 238 (215, 261)
Khedr et al[105]	Community-based cross-sectional study involving door-to-door screening within 10 randomly sampled areas in the Qena governate, Egypt.	8,027	All ages	***PD:*** 62 (8, 117)	.
Yang et al[106]	Cohort study involving door-to-door screening using a hierarchical random cluster sampling method.	5,932	≥ 45 years	.	.
Zou et al[107]	Systematic review and meta-analysis of PD incidence and prevalence studies in China, Taiwan and Hong Kong. 15 included studies, but not directly comparable due to significant methodological heterogeneity.	.	All ages	.	**Annual PD incidence (2 studies):** 1.5 - 8.7
Liu et al[108]	Population-based study using healthcare records from the National Health Insurance (NHI) database.	≈ 23 million	≥ 40 years	***2002-2003:*** 34.3; ***2004-2005:*** 35.2; ***2006-2007:*** 36.9; ***2008-2009***: 36.6	***2002-2003:*** 33.5; ***2004-2005:*** 34.5; ***2006-2007:*** 36.2; ***2008-2009:*** 36.6

Author	Study design	Population size	Age	Crude incidence rate per 100,000	Adjusted / standardised incidence rate per 100,000
GBD 2016[109]	Population-based systematic analysis of PD epidemiological studies, involving an update to the GBD 2013 PubMed search to identify additional studies published between 01/01/2011 and 31/12/2015.	.	All ages	.	.
Muangpaisan et al[110]	Cohort study involving door-to-door screening using an 11-item PD-screening tool in 6 suburban areas within Bangkok, Thailand.	1846	≥ 50 years	.	.
Abbas et al[111]	Review of English original research studies, reviews and meta-analyses, published before May 2017, with WHO 2000 standardized PD incidence and / or prevalence rates in the East and West.	.	All ages	.	***Door-to-door surveys (East):*** 1.5 - 8.7; ***Door-to-door surveys (West):*** 15.4 - 27.6; ***Record-based studies (East):*** 6.7 - 36.6; ***Record-based studies (West):*** 6.1 - 17.4
Williams et al[112]	Systematic review of population, outpatient, clinic and inpatient PD epidemiological, genetic and care-related studies published up to May 2016. Included 7 community door-to-door prevalence studies.	.	All ages	.	.
Fleury et al[113]	Cross-sectional population-based study involving analysis of healthcare records, followed by case verification by a Neurologist.	Prevalence: 470512; incidence 2009-2012: 1858977	All ages	***PD:*** 16.7 (14.9, 18.7); ***PSP:*** 1.9 (1.3, 2.6); ***MSA:*** 0.8 (0.4, 1.3); ***CBS:*** 0.8 (0.4, 1.3); ***DLB:*** 4.5 (3.6, 5.6); ***VP:*** 2.6 (1.9, 3.4); ***DIP:*** 2.5 (1.8, 3.3)	***PD:*** 12.0 (10.6, 13.4); ***PSP:*** 1.3 (0.9, 1.8); ***MSA:*** 0.6 (0.3, 0.9); ***CBS:*** 0.6 (0.3, 0.9); ***DLB:*** 2.7 (2.1, 3.3); ***VP:*** 1.5 (1.1, 1.9); ***DIP:*** 1.8 (1.3, 2.4)
Kadastik-Eerme et al[114]	Community-based cross-sectional study involving primary and secondary care, and insurance records.	152,188	All ages	.	.
Valent et al[115]	Cross-sectional study involving administrative records from 5 regional databases.	1,217,936	All ages	28	.
Szatmári et al[116]	Cross-sectional study involving primary and secondary care records, and medication data.	10 million	All ages	49 (45, 53)	56 (51, 60)

Author	Study design	Population size	Age	Crude incidence rate per 100,000	Adjusted / standardised incidence rate per 100,000
Park et al[117]	Population-based study using health insurance data from the South Korean national registry database.	***2010:*** 50,087,000 ***2011:*** 50,362,000 ***2012:*** 50,675,000 ***2013:*** 50,922,000 ***2014:*** 51,187,000 ***2015:*** 51,473,000.	All ages	.	***2010:*** 23.2; ***2011:*** 22.9; ***2012:*** 22.4; ***2013:*** 22.6; ***2014:*** 24.2; ***2015:*** 27.8
Han et al[118]	Population-based involving claims and prescription data from the Health Insurance Review and Assessment Service database. Retrospective cross-sectional design was used to assess prevalence and a retrospective cohort study for incidence.	All ages	≈ 50 million	***2012 PD incidence:*** 35.4; ***2015 PD incidence:*** 33.3; ***2012 DIP incidence:*** 7.1; ***2015 DIP incidence:*** 13.9	***Average standardized PD incidence:*** 23.5 (WHO 2000) and 35.8 (US 2000); ***Average age-standardized DIP incidence:*** 7.1 (WHO 2000) and 8.0 (US 2000)
GBD 2021[119]	Population-based systematic analysis of all global PD epidemiological studies to update to GBD 2016.	.	All ages	.	***Global incidence rate 2003:*** 10.4 (9.5, 11.5); ***UK incidence rate 2003:*** 27.5 (23.8, 31.1); ***Global incidence rate 2019:*** 16.1 (14.5, 17.9); ***UK incidence rate 2019:*** 32.7 (28.3, 37.2); ***Global incidence rate 2021:*** 16.9 (15.2, 18.8); ***UK incidence rate 2021:*** 33.5 (28.8, 38.0)
Qi et al[120]	Population-based study involving multistage clustered sampling with a 9-item questionnaire followed by clinical examination.	24,117	≥ 60 years	.	.
Song et al[121]	Population-based cross-sectional study involving multistage cluster sampling, with door-to-door screening, in 11 urban and 10 rural regions in China.	8,124	≥ 65 years	.	.
Cicero et al[122]	Cross-sectional study involving data from three administrative databases (hospital discharges, medical exemptions and drug prescriptions).	5,026,989	All ages	.	.

Author	Study design	Population size	Age	Crude incidence rate per 100,000	Adjusted / standardised incidence rate per 100,000
Llibre-Guerra et al[123]	Multinational population-based cohort study in rural and urban areas within 6 Latin American countries. Door-to-door survey performed, followed by a comprehensive 3-hour interview, examination and informant interview.	11,613	≥ 65 years	.	.
Varden et al[124]	Review of previously published UK prevalence studies from 1955 to April 2023. 10 studied included but significant methodological variation in case ascertainment and diagnosis precluded direct comparison.	.	All ages	.	.
Zhu et al[125]	Systematic review and meta-analysis of 83 observational studies from 37 countries evaluating PD prevalence from database inception to 01/11/2023.	37,553,417 participants	All ages	.	.
Kim et al[126]	Systematic review and meta-analysis of epidemiological studies evaluating the incidence and / or prevalence of PD and parkinsonism using Medline, Embase, Scopus, Web of Science, Latin American and Caribbean Health Science literature databases, and Scientific Electronic Library Online from inception until 2022.	Nearly 4 million	All ages	***PD:*** 31 (23, 40)	.
Bower et al[127]	Population-based cohort study involving medical records from the Rochester Epidemiology project. Diagnoses verified by Neurologist review of case records where applicable.	***Total:*** 1,424,474; ***50 - 99 years:*** 301,639	All ages	***PSP incidence (all ages):*** 1.1; ***PSP incidence rate ≥50 years:*** 5.3; ***MSA incidence (all ages):*** 0.6; ***MSA incidence rate ≥ 50 years:*** 3.0	.
Schrag et al[128]	Cross-sectional study involving computerised primary care records, followed by clinical assessment.	121,608; 14,272 ≥ 65 years	All ages	.	.

Author	Study design	Population size	Age	Crude incidence rate per 100,000	Adjusted / standardised incidence rate per 100,000
Kawashima et al[129]	Community-based prevalence study involving two surveys (1999, 2002) over a 3-year period, with data derived from hospital medical records and nursing homes. Cases verified by clinical examination.	137,420	All ages	.	.
Wada-Isoe et al[130]	Population-based study involving door-to-door screening with clinical examination of cases that screened positive.	943	≥ 65 years	.	.
Vann Jones et al[131]	Systematic review and meta-analysis of population-based and clinical DLB epidemiological studies.	***Incidence studies:*** 9756 subjects; ***Prevalence studies:*** 26,137 subjects	> 65	***Total DLB incidence:*** 87 (57, 140) per 100,000 person years	.
Hogan et al[132]	Systematic review and meta-analysis of studies evaluating DLB incidence and / or prevalence, identified via MEDLINE and EMBASE database searches. Significant between-study heterogeneity and small sample sizes precluded a pooled meta-analysis.	.	All ages	***DLB incidence:*** 50 - 160	.
Takigawa et al[133]	Community-based prevalence study involving six surveys from 2009 - 2014, with data derived from secondary care medical records. Cases clinically assessed for diagnostic verification.	148,271	All ages	.	.
Savica et al[134]	Population-based study involving medical records from the Rochester Epidemiology Project. Diagnostic verification by review of medical records by a Movement Disorder specialist.	.	All ages	***Average annual DIP incidence:*** 3.3	***DIP incidence (all years):*** 3.8; ***1976-1985:*** 5.7; ***1986-1995:*** 3.7; ***1996-2005:*** 2.6
Stang et al[135]	Population-based cohort study involving medical records from the Rochester Epidemiology project. Diagnoses verified by Neurologist review of case records.	1,852,762 person-years	All ages	**PSP:** 2.6; **CBS:** 0.4; **CBS+PSP combined:** 3.1	.

Author	Study design	Population size	Age	Crude incidence rate per 100,000	Adjusted / standardised incidence rate per 100,000
Swallow et al[136]	Systematic review of 16 PSP and 9 CBS prevalence studies using MEDLINE, EMBASE, Web of Science, LILACS and CINAHL databases.	.	All ages	.	.
Swallow et al[137]	Population-based study involving analysis of national and regional (Grampian) electronic health data. Case verification by review of notes and / or examination (in a proportion of cases).	**Scotland:** 5,438,100; **Grampian:** 584,550	All ages	.	.
Lyons et al[138]	Systematic review of 32 PSP and CBS incidence and prevalence studies, using the PubMed and EMBASE databases from inception to 13/07/2021.	.	All ages	***PSP incidence:*** 0.16 (0.07, 0.39) - 2.6 ***CBS incidence:*** 0.03 (0, 0.18) - 0.8 (0.4, 1.3)	.
Logroscino et al[139]	Retrospective cohort study using population-based registry from 13 tertiary research clinics in 9 European countries.	11,023,643 person years	All ages	.	***Combined PSP and CBS European incidence:*** 0.5 (0.2, 1.2)
Kaplan et al[140]	Literature review of PubMed and EMBASE , including 24 studies conducted in 14 countries from 1995 - 2022.	.	All ages	.	.

Author	Crude prevalence rate per 100,000	Adjusted / standardised prevalence rate per 100,000	Notes
MacDonald et al[1]	*Lifetime prevalence:* 200 (100, 300)	.	Rates were age and sex-adjusted to the 1991 UK population census.
Foltynie et al[2]	.	.	Rates were standardized to the 1991 ESP.
Taylor et al[3]	.	.	Rates were standardized to the 1990 Scottish population.
Caslake et al[4]	.	.	No association between SES and PD but SES based on postcode rather than patient-specific. Crude rates for other non-PD parkinsonisms derived.
Horsfall et al[5]	.	.	Downward trend in adjusted incidence between 1-6% per year depending on case definition.12% (CI 4, 20) higher adjusted incidence in urban than rural areas. Lower incidence in more deprived areas after adjustment for age and sex with narrow case definition.
Evans et al[6]	.	.	Rates were directly age-standardized to the UK population using 2008 ONS estimates.
Duncan et al[7]	.	.	Rates were age-standardized to the ESP.
Okunoye et al[8]	.	.	Stable adjusted incidence between 2006 - 2016 using the broadest case definition, but slightly declining trend with more stringent definitions. Highest adjusted incidence in Northern Ireland and in the least deprived.
Harada et al[9]	.	.	Higher crude incidence rate in women than men (no adjustment / standardization).
Morens et al[10]	.	.	Rates were adjusted to the 1970 US population.
Bower et al[11]	.	.	
Baldereschi et al[12]	.	.	Rates were adjusted to the 1992 Italian population.
Morioka et al[13]	.	.	Rates were age adjusted using the 1985 Japanese census. Male to female ratio: 1: 1.4.
Van den Eeden et al[14]	.	.	Rates were age- and sex-adjusted to the 1990 US population. Noted variation by ethnicity: highest adjusted incidence rate for Hispanics, followed by non-Hispanic Whites, then Asians and Blacks.
Twelves et al[15]	.	.	Slightly earlier onset in men than women. Significantly greater incidence in men than women in 5/9 studies with age-standardized sex ratios.
de Lau et al[16]	.	.	
Tan et al[17]	.	.	Rates were age- and sex-adjusted to the 1990 US population. Similar incidence rates between men and women Incidence rates varied by ethnicity between Chinese, Malays and Indians (P = 0.03), but numbers were small.
Alves et al[18]	.	.	Rates were age-standardized to the 1991 ESP.
Perez et al[19]	.	.	
Linder et al[20]	.	.	Rates were age-standardized to the average Swedish population 2004 - 2007.
Winter et al[21]	.	.	Rates were directly age-standardized to the 2007 Russian population.
Savica et al[22]	.	.	PD rate adjusted to 1990 US census. Increasing incidence with age and most proteinopathies are more frequent in men than women.

Author	Crude prevalence rate per 100,000	Adjusted / standardised prevalence rate per 100,000	Notes
Hirsch et al[23]	.	.	Higher incidence in men than women but only statistically significant for ages 60-69y and 70-79y.
Savica et al[24]	.	.	Standardized to the total 1990 US population. Significant increase in PD incidence rates over 30 year period, RR 1.24 per decade (95% CI 1.08, 1.43).
Darweesh et al[25]	.	.	Sharp decline in adjusted PD incidence between 1990 and 2000 subcohorts, RR 0.39 (0.19, 0.72).
Canonico et al[26]	.	.	Only included women aged 40- 65 years at baseline. No significant changes in incidence rates from 1992 - 2018.
Brakedal et al[27]	.	.	No significant time trends in incidence. PD prevalence significantly increased for all ages, except cases aged 30 - 59 years.
Dammertz et al[28]	.	.	Decline in age- and sex-standardized PD incidence from 2013-2019 in Germany.
Fink et al[29]	.	.	PD incidence declined over time in Germany, even after adjustment for age, sex, changes in mortality and PD risk factors.
Brewis et al[30]	112	.	
Sutcliffe et al[31]	108 (95, 124)	.	
Mutch et al[32]	.	164	
Sutcliffe and Meara[33]	121	.	Increase in prevalence from 108 to 121 per 100,000 from 1982 to 1992.
Schrag et al[34]	128 (109, 150)	168 (142, 195)	Rates were age-adjusted by direct standardization to the 1997 UK population.
Hobson et al[35]	144 (120, 173)	105 (85, 124)	Prevalence rates were age-adjusted by direct standardization to the mid-1998 UK population.
Porter et al[36]	148 (124, 174)	139 (116, 162)	
Wickremaratchi et al[37]	130 (117, 144)	142 (128, 156)	Prevalence rates were standardized to the 1997 England and Wales population and were relatively stable across 40 years in the UK. Global average prevalence from meta-analysis of current and previous UK prevalence studies: 140 per 100,000, but studies were statistically heterogeneous (P = 0.0006).
Newman et al[38]	119 (110, 129)	130 (120, 139)	Age-adjusted rates were standardized to the 2000 Scottish population. Significantly higher adjusted prevalence in South Lanarkshire than South Glasgow (smoking rates, educational deprivation and urbanicity levels are greater in South Glasgow than South Lanarkshire).
Walker et al[39]	178 (144, 212)	142 (118, 165)	Rates were age-adjusted by direct standardization to the 2001 UK population.
Parkinson's UK[40]	***All ages:*** 210<br>***≥18:*** 267<br>***≥ 20y:*** 275	.	
Li et al[41]	***All ages:*** 44 (29, 64);<br>***> 50y:*** 198 (132, 287)	57 (35, 79)	Rates were directly standardized to the 1960 US population.
Bharucha et al[42]	328.3	192	Rates were age-adjusted to the 1960 US population.

Author	Crude prevalence rate per 100,000	Adjusted / standardised prevalence rate per 100,000	Notes
Schoenberg et al[43]	***Copiah County, Black ethnicity:*** 341; ***Copiah County, White ethnicity:*** 352; ***Igbo-Ora, Black ethnicity:*** 59	***Copiah County, Black ethnicity:*** 341; ***Copiah County, White ethnicity:*** 353; ***Igbo-Ora, Black ethnicity:*** 67	Data were age-adjusted using an indirect method to account for differences in the age distributions of the two populations.
Granieri et al[44]	165 (147, 185)	130	Rates were age- and sex-adjusted to the Italian population. Incidence was higher among agricultural workers and early onset PD significantly more likely in rural than urban areas.
Morgante et al[45]	**PD all ages:** 257; ***PD 40+:*** 651.9; ***DIP:*** 32.7; ***VP:*** 28.6 ***Unspecified:*** 28.6	.	
Caradoc-Davies et al[46]	110	76	Rates were adjusted to 1960 US census.
Tison et al[47]	***Overall:*** 1400 (1000, 1800)	.	
de Rijk et al[48]	***PD:*** 1392 ***DIP:*** 43.0 ***VP:*** 14.3 ***MSA:*** 28.6 ***PSP:*** 14.3	.	
Mayeux et al[49]	106.9 (93.0, 120.8)	.	Incident and prevalent rates were discordant for African-Americans (lower PD prevalence, but higher incidence in African-American men than White men).
Tandberg et al[50]	111	102	Prevalence rates were directly age standardized to the ESP.<br>Higher age-adjusted prevalence rates in rural than urban settings.
Kusumi et al[51]	118	***1980:*** 104; ***1992:*** 99.5	Rates were age-adjusted to the 1990 Japanese population.
Moriwaka et al[52]	***Hokkaido:*** 94.7; ***Iwamizawa City:*** 95.8	***Iwamizawa City:*** 71.2 (52.8, 89.6)	Prevalence rate for Iwamizawa city was age-adjusted to the 1970 US population.
Wang et al[53]	587 (373, 884)	119 (80, 169)	Rates were standardized to the 1970 US population.
Fall et al[54]	115	76	Rates were standardized to the ESP.
de Rijk et al[55]	.	1600	Rates were standardized to the 1991 ESP.
Melcon et al[56]	***PD:*** 657; ***DIP:*** 103; ***VP:*** 90.1;	.	
Wermuth et al[57]	***Overall PD:*** 187.6; ***≥ PD 50 years:*** 654; ***PSP:*** 4.6; ***MSA:*** 2.3; ***VP:*** 11.4	***PD:*** 183.3	Rates were adjusted to the Rogaland county in Norway on January 1, 1995.
Chiò et al[58]	168 (138, 204)	135 (111, 164)	Rates were adjusted for sex and age to the 1991 Italian population using direct standardization. Based on levodopa prescriptions alone, crude prevalence was 196 (163, 235), with prevalence overestimated in older patients and women, and underestimated in younger milder cases.

Author	Crude prevalence rate per 100,000	Adjusted / standardised prevalence rate per 100,000	Notes
Errea et al[59]	***PD:*** 221 (185, 256);<br>***DIP:*** 42.8;<br>***VP:*** 9.9;<br>***MSA:*** 1.6;<br>***Unspecified:*** 4.9	121.9	Rates were age-adjusted to the ESP.
Kuopio et al[60]	***1971:*** 116;<br>***1992:*** 196	***1971:*** 139 (120, 157);<br>***1992:*** 166 (149, 182);	Rates were age-adjusted to the Finnish general population in 1991.<br>Age-adjusted prevalence moderately increased from 1971 to 1992. Age-adjusted incidence increased in men but decreased in women from 1971 to 1992. Signal rural predominance in 1992, not present in 1971.
Wermuth et al[61]	102	98.3	
Chen et al[62]	367.9 (242.5, 460.5)	***All ages:*** 130.1;<br>***40+:*** 357.9	Rates were age-adjusted to the 1970 US census.
Milanov et al[63]	***Trojan:*** 164;<br>***Veliko Turnovo:*** 170	552	
Wermuth et al[64]	81.0	187.5	Rates were age-adjusted to the population in Denmark.
Kis et al[65]	**PD:** 1600	***PD:*** 1500 (600 - 2300)	Rates were age adjusted to the 1991 ESP.
Claveria et al[66]	1267 (774, 1951)	901	Rates were age-adjusted to the ESP.
Anca et al[67]	***All ages:*** 240;<br>***> 40y:*** 233;<br>***> 60y:*** 179	***> 40y:*** 337;<br>***> 60y:*** 942	
Taba and Asser[68]	176 (150, 202)	.	Higher adjusted prevalence rates in urban than rural areas (urban: rural ratio 1.15).
Kimura et al[69]	119	***Overall:*** 76.6;<br>***Males:*** 61.3;<br>***Females:*** 91.0	Rates were adjusted to the 1995 population census in Japan. Significantly higher PD prevalence in women than men.
Nicoletti et al[70]	***All ages:*** 50.2 (18.5, 124.5)<br>***40+:*** 286 (28, 543)	***All ages:*** 97.3;<br>***40+:*** 304	Rates were age-adjusted to the world standard population.
Benito-Leon et al[71]	***PD:*** 1500 (1200, 1800);<br>***DIP:*** 500 (300, 700)<br>***VP:*** 100 (0, 200);	.	
Zhang et al[72]	***PD:*** 1100;<br>***PSP:*** 17.4;<br>***MSA:*** 17.4;<br>***DLB:*** 69.7;<br>***DIP:*** 104;<br>***VP:*** 331	.	
Bergareche et al[73]	1500 (900, 2300)	.	
Tan et al[74]	290 (220, 390)	***Standardized to UICC world population:***<br>250 (180, 340)<br>S***tandardized to the 1970 US population:***<br>300 (220, 410)	No significant variation in rates by ethnicity between Chinese, Maly and Indians Singaporean residents.
Sanchez et al[75]	***Overall:*** 30.7 (29.2, 32.2);<br>***> 50 years*** : 176 (167, 186)	.	

Author	Crude prevalence rate per 100,000	Adjusted / standardised prevalence rate per 100,000	Notes
Totaro et al[76]	229 (213, 247)	163.6	Prevalence rates were directly standardized to the 1996 European population.
Chan et al[77]	780 (546, 1077)	.	
Zhang et al[78]	1070 (960, 1190)	***Standardized to China:*** 960 (850, 1080); ***Standardized to USA:*** 1340 (1210, 1480)	Rates were directly standardized to the 2000 US census and 1999 Chinese census.
Zhang et al[79]	.	522 (477, 567)	Prevalence rates were standardized using the reference population in Linxian county.
Von Campenhausen et al[80]	***PD crude prevalence:*** 65.6 - 12,500; ***High quality studies only:*** 108 - 257		
Okubadejo et al[81]	***Nigeria (1982):*** 10; ***Libya (1982):*** 31.4; ***Tunisia (1985):*** 43; ***Ethiopia (1986):*** 7; ***Togo (1989; 1995);*** 20		
Barbosa et al[82]	***PD:*** 3300 (2200, 4400); ***DIP:*** 2700 (1700, 3700); ***VP:*** 1100 (400, 1800)	.	
Mehta et al[83]	460 (230, 680)	***Standardized to the 2001 Australian population (50+):*** 362 (183, 541); S***tandardized to the 2001 Australian population (all ages):*** 104	Rates were age-standardized to the 2001 Australian population
Wermuth et al[84]	***PD:*** 206.7; ***MSA:*** 6.2; ***PSP:*** 4.1	***PD:*** 218	Rates were age-adjusted to the 1995 population of Rogaland, Norway
Dotchin et al[85]	20	40	Rates were age-standardized to the 2001 UK population
Morgante et al[86]	104 (59.4, 171)	152	Rates were age-adjusted using the 2001 census for the Italian population
Alrefai et al[87]	58.8 (47.4, 70.2)		
Yamawaki et al[88]	***Yonago 2004:*** 180 (158, 202); ***Daisen 2004:*** 307 (176, 438)	***Yonago 1980:*** 146 (145, 147); ***Yonago 1992:*** 147 (146, 148); ***Yonago 2004:*** 167 (166, 168) ***Daisen 2004:*** 193 (192, 194)	Prevalence rates were age- and sex-adjusted to the 2004 Japanese population. 1980 population in Yonago used for age- and sex-adjusted incidence. Adjusted prevalence increased, but incidence remained stable over time.
Chen et al[89]	706 (551, 864)		Rates were age-adjusted to the 1970 and 2000 US census.
Muangpaisan et al[90]		***Door-to-door studies:*** 51.3-177; ***Record-based studies:*** 35.8-68.3	Rates were standardized to the 2000 WHO population.

Author	Crude prevalence rate per 100,000	Adjusted / standardised prevalence rate per 100,000	Notes
Lix et al[91]	.	***2006/2007 males*** *:* 504 (471, 539); ***2006/2007 females:*** 393 (366, 422)	PD prevalence increased over 20 years but incidence remained stable. In rural regions, average prevalence estimates were significantly higher for the lowest income quintile, but incidence estimates were similar between income quintiles
Masalha et al[92]	***Overall:*** 43.2; ***PD ≥ 65 years:*** 477		
Das et al[93]	40.67 (29.04, 55.40)	52.85 (37.74, 71.98)	Adjusted to the WHO 2000 World Standard Population
Wright Willis et al[94]	***Mean PD prevalence ≥ 65 years 1995, and 2000-2005:*** 1588 (± 97.4)	.	Stable incidence and prevalence over 10 years from 1995 - 2005. Mean standardized prevalence was highest in White men and lowest in Asian women. Mean standardized prevalence was 50% lower in cases of Black and Asian than White ethnicity. Higher incidence and prevalence in urban than rural counties. Non-random increased PD incidence and prevalence in Midwest and Northeast on Bayesian mapping
Osaki et al[95]	***PD:*** 175 (143, 206); ***PSP:*** 18 (8, 28); ***MSA:*** 17 (7, 26); ***CBD:*** 9 (2, 16)	***PD:*** 109 (88, 134); ***PSP:*** 10 (2, 17); ***MSA:*** 13 (4, 21); ***CBD:*** 6 (0, 12)	Rates were directly standardized to the 2005 Japanese consensus
Seijo-Martinez et al[96]	1990 (930, 3060)	1700	Rates were age adjusted to the ESP
Khedr et al[97]	***PD:*** 557 (367, 748); ***PD cases ≥ 50y:*** 2748 (1810, 3685) ***DIP:*** 51 (0, 108); ***VP / post stroke:*** 34 (0, 81)	***Age-adjusted PD prevalence rate:*** 562	
Gordon et al[98]	144	356	No significant changes in age-adjusted prevalence comparing 2002-2005, and 2006-2009. PD prevalent amongst American Indian and Alaskan Native individuals.
Gordon et al[99]	***2005-2009:*** 204	***2005-2009:*** 336 (278, 394); ***2000-2004:*** 285 (227, 342); ***1995-1999:*** 247 (187, 306)	Rates were age-standardized to the 2000 US population. Increase in age-standardized prevalence over time from 1995 to 2009.
El-Tallawy et al[100]	***PD:*** 213 (151, 286); ***VP:*** 90.4 (49.6, 138); ***Unspecified parkinsonism:*** 6.5 (-4.5, 11.1)		
Blanckenberg et al[101]	**Range:** 7 - 20 per 100,000		
Pringsheim et al[102]	***Overall PD prevalence:*** 315 (113, 873); ***Overall PD prevalence for studies with quality score ≥ 7:*** 571 (243, 1339)		Significantly higher prevalence in North America, Europe and Australia than in Asia only for cases 70 - 79 years old. Significantly higher prevalence in males than females only for cases 50 - 59 years.

Author	Crude prevalence rate per 100,000	Adjusted / standardised prevalence rate per 100,000	Notes
Blin et al[103]	.	***Specific 2010 prevalence:*** 308 (307, 309); ***Sensitive 2010 prevalence:*** 410 (408, 411)	Rates were extrapolated to the whole of France and standardized by 5-year age bands and sex to the French population. Specific: highly probable PD; sensitive: highly probable and probable.
Gordon et al[104]	146 (130, 162)	***Age-adjusted point prevalence rate:*** 261 (232, 290)	
Khedr et al[105]	**PD:** 436 (292, 580); **Age-specific PD prevalence rate ≥ 50y:** 2534 (1695, 3374) **DIP:** 37 (0, 80); ***VP:*** 25 (0, 59); ***CBD:*** 25 (0, 59)	.	
Yang et al[106]	1480	.	
Zou et al[107]	.	***PD prevalence (15 studies):*** 16 - 440	Rates were standardized to the WHO 2000 population
Liu et al[108]	***2002-2003:*** 164.1; ***2004-2005:*** 214.5; ***2006-2007:*** 260.6; ***2008-2009:*** 306.8	***2002-2003:*** 159.8; ***2004-2005:*** 209.7; ***2006-2007:*** 256.5; ***2008-2009:*** 299.3	Rates were age and sex standardized to the WHO 2000 population. 9.2% increase in standardized PD incidence from 2002 - 2009. 1.88 fold / 87.3% increase in standardized prevalence from 2002 - 2009. Greater adjusted incidence and prevalence rate ratios in rural than urban areas.
GBD 2016[109]	.	***Prevalence 2016 UK counts:*** 115,846 (91,722, 144.139)	Age standardized prevalence rates increased by 22.3% (20.0, 24.7) in the UK and by 21.7% (18.1, 25.3) globally from 1990 - 2016.
Muangpaisan et al[110]		706	Rates were directly standardized to the 2000 WHO population.
Abbas et al[111]		***Door-to-door studies (East):*** 16.7 - 440.3; ***Door-to-door surveys (West):*** 101 - 439.4; ***Record-based studies (East):*** 35.8 - 299.3; ***Record-based studies (West):*** 70.2-141.1	PD incidence was lower in the East than the West, except in a record-based Taiwanese study (Liu et al 2016). Overall lower PD prevalence in the East than the West.
Williams et al[112]		**Range:** 7 per 100,000 in Ethiopia - 67/100,000 in Nigeria (latter age-adjusted)	No incidence studies identified.
Fleury et al[113]	***PD:*** 173 (161, 185); ***PSP:*** 8.3 (5.9, 11.3); ***MSA:*** 4.0 (2.4, 6.3); ***CBS:*** 3.0 (1.6, 5.0); ***DLB:*** 19.1 (15.4, 23.5); ***DIP:*** 21.7 (17.7, 26.3); ***VP:*** 18.5 (14.8, 22.8)	***PD:*** 114 (106, 122); ***PSP:*** 5.7 (3.8, 7.6); ***MSA:*** 3.1 (1.7 , 4.6); ***CBS:*** 2.1 (1.0, 3.3); ***DLB:*** 10.9 (8.5, 13.2); ***DIP:*** 14.9 (11.8, 17.9); ***VP:*** 10.2 (8.0, 12.5)	Rates were standardized to the ESP.

Author	Crude prevalence rate per 100,000	Adjusted / standardised prevalence rate per 100,000	Notes
Kadastik-Eerme et al[114]	283 (257, 310)	***Standardized to the 2014 Estonian population:*** 314 (285, 344); ***Standardized to 2011 ESP:*** 324 ***Standardized to the 1989 Estonian population:*** 197 (178, 216)	Moderate increase in age-adjusted prevalence from 152 to 197 per 100,000 over 20 years in Estonia. No significant difference in adjusted prevalence between rural and urban areas.
Valent et al[115]	389		
Szatmári et al[116]	404 (392, 416)	471 (456, 485)	Rates were standardized to the 2013 ESP.
Park et al[117]		***2010:*** 116; ***2011:*** 122; ***2012:*** 126; ***2013:*** 129; ***2014:*** 134 ***2015:*** 140.	Rates were standardized by age and sex to the 2010 Korean population. Increase in standardized PD incidence and prevalence from 2010 - 2015. Standardized incidence increased in men, but remained static in women until 2013, after which it increased. Higher incidence and prevalence in women than men in Korea.
Han et al[118]	***2012 PD prevalence:*** 157; ***2015 PD prevalence:*** 181; ***2012 DIP prevalence:*** 7.3; ***2015 DIP prevalence:*** 15.4	***Average age-standardized PD prevalence between 2012-2015:*** 114 (WHO 2000 population) and 176 (US 2000 population); ***Average age-standardized DIP prevalence between 2012 - 2015:*** 8.0 (WHO 2000) and 9.0 (US 2000)	PD incidence decreased, while DIP incidence increased, from 2012 - 2015. Higher PD incidence and prevalence in females than males (opposite to Western studies). Higher DIP incidence and prevalence in females.
GBD 2021[119]	.	***2003 global prevalence rate:*** 85.6 (76.5, 96.7); ***2003 UK prevalence rate:*** 258 (220, 299) ***2019 global prevalence rate:*** 141 (125, 161); ***2019 UK prevalence rate:*** 286 (243, 331) ***2021 global prevalence rate:*** 149.1 (132.3, 170.1); ***2021 UK prevalence rate:*** 292 (249, 340)	***2003 UK prevalence number:*** 154,245.3 (131,584.9, 178,527.1); ***2019 UK prevalence number:*** 192,223.7 (163,563.9, 222,782.9); ***2021 UK prevalence number:*** 198,388.4 (168,849.3, 230,358.8); ***2003 UK incidence number:*** 16,414.2 (14,237.7, 18,601.5); ***2019 UK incidence number:*** 22,006.3 (19,037.0, 25,016.7); ***2021 UK incidence number:*** 22,693.3 (19,560.2, 25,807.8)
Qi et al[120]	1370 (1020, 1730)	.	Significantly higher PD prevalence in rural than urban settings.
Song et al[121]	1860 (1600, 2200)	1600	Higher standardized prevalence with increasing age, male sex, and in urban than rural areas.
Cicero et al[122]	***All ages:*** 488 (482, 494); ***60+:*** 1528 (1510, 1550)		
Llibre-Guerra et al[123]		2000 (1700, 2300)	No significant differences in prevalence between rural and urban areas, but limited by small size of rural populations.
Varden et al[124]		***Age-adjusted PD prevalence estimates in the UK:*** 105 - 168	Stable prevalence between 1961 and 2007.

Author	Crude prevalence rate per 100,000	Adjusted / standardised prevalence rate per 100,000	Notes
Zhu et al[125]	***Global pooled PD prevalence:*** 151 (119, 188); ***1980-1989:*** 90 (48, 144); ***1990-1999:*** 138 (117, 161); ***2000-2009:*** 118 (77, 167); ***2010-2023:*** 381 (267, 514)		Higher PD prevalence in males than females and in higher SDI and / or HDI countries. Increasing prevalence over time. Significantly higher estimated annual percentage change in PD prevalence from 2004-2023 vs. 1980-2003.
Kim et al[126]	***PD:*** 472 (271, 820)	.	Increasing PD prevalence with increasing age, and in cohort studies than administrative databases, but no variation by sex.
Bower et al[127]			PSP incidence consistently increased with age and was higher in men than women. No cases < 50 years.
Schrag et al[128]	***PSP:*** 4.9 (1.8, 10.7); ***MSA:*** 3.3 (0.9, 8.4)	***Age-adjusted PSP prevalence:*** 6.4 (2.3, 10.6); ***Age-adjusted MSA prevalence:*** 4.4 (1.2, 7.6)	Rates were age standardized to the 1996 ESP.
Kawashima et al[129]	***PSP:*** 5.8 (1.8, 9.9)	5.0 (males: 7.9, females 2.3)	Rates were directly standardized by age and sex to 2000 Japanese population. Higher standardized prevalence in men than women.
Wada-Isoe et al[130]	***DLB:*** 530; ***PSP:*** 210	.	
Vann Jones et al[131]	**Mean DLB prevalence in whole population:** 360 (290, 440)		Significantly higher rates of DLB diagnosis in secondary care than in community-settings (7.5 vs. 4.2% of all diagnosed dementias, respectively). DLB diagnosis increased significantly with the 2005 revised DLB International Consensus Criteria compared to the original 1996 criteria.
Hogan et al[132]	***Prevalence:*** 2 - 6350	.	
Takigawa et al[133]	***PSP:*** 17.9 (12.1, 26.4)	***PSP:*** 17.3 (17.0, 17.5)	Rates were directly standardized to the Japanese population on 01/10/2010. Increase in PSP prevalence in Yonago city from 1999 - 2010. Similar rates in males and females.
Savica et al[134]			Rates were directly age-standardized to the total 1990 US population. Higher DIP incidence in women and with increasing age. DIP incidence decreased by 32.0% per decade and by 68.6% over the 30 years (statistically significant only in females).
Stang et al[135]			PSP and CBS incidence increased significantly from 1991 - 2005. Higher incidence in men than women.
Swallow et al[136]	***Pooled PSP prevalence (based on 3 studies):*** 7.1; ***Pooled CBS prevalence (2 studies):*** 2.3	.	Heterogenous case definitions, ascertainment and verification across studies and limited quality precluded a full meta-analysis. Some evidence of increasing prevalence with age. No clear evidence of sex differences or increasing secular trends for PSP based on crude rates.

Author	Crude prevalence rate per 100,000	Adjusted / standardised prevalence rate per 100,000	Notes
Swallow et al[137]	**National, PSP:** 2.5 (2.1, 2.9); **National, CBS:** 0.9 (0.7, 1.2); **Grampian, PSP:** 4.3 (2.9, 6.3); **Grampian, CBS:** 2.1 (1.2, 3.6)		No clear sex differences in PSP or CBS prevalence. Increasing PSP and CBS prevalence with age, peaking between 70 - 79 years nationally.
Lyons et al[138]	***Pooled PSP prevalence:*** 6.9 (4.3, 11.1); ***Pooled CBS prevalence:*** 3.9 (2.0, 7.5)		Increase in PSP prevalence with age, with a general male predominance.
Logroscino et al[139]			Rates were standardized to the 2013 ESP and were higher in men than women.
Kaplan et al[140]	***Crude MSA prevalence:*** 0.5 (Spain) - 17 per 100,000 (Japan)	.	

CBS: Corticobasal Syndrome; DIP: Drug-Induced Parkinsonism; DLB: Dementia with Lewy bodies; ESP: European Standard Population; HDI: human development index; MSA: Multiple System Atrophy; ONS: Office of National Statistics; PD: Parkinson's disease; PSP: Progressive Supranuclear Palsy; SDI: sociodemographic index; SES: socioeconomic status; UK: United Kingdom; US: United States; VP: Vascular Parkinsonism; WHO: World Health Organisation.

References:

1. MacDonald BK, Cockerell OC, Sander JW, Shorvon SD. The incidence and lifetime prevalence of neurological disorders in a prospective community-based study in the UK. Brain 2000;123 (Pt 4):665-676.
2. Foltynie T, Brayne CE, Robbins TW, Barker RA. The cognitive ability of an incident cohort of Parkinson's patients in the UK. The CamPaIGN study. Brain 2004;127(Pt 3):550-560.
3. Taylor KS, Counsell CE, Harris CE, Gordon JC, Smith WC. Pilot study of the incidence and prognosis of degenerative Parkinsonian disorders in Aberdeen, United Kingdom: methods and preliminary results. Mov Disord 2006;21(7):976-982.
4. Caslake R, Taylor K, Scott N, et al. Age-, gender-, and socioeconomic status-specific incidence of Parkinson's disease and parkinsonism in northeast Scotland: the PINE study. Parkinsonism Relat Disord 2013;19(5):515-521.
5. Horsfall L, Petersen I, Walters K, Schrag A. Time trends in incidence of Parkinson's disease diagnosis in UK primary care. J Neurol 2013;260(5):1351-1357.
6. Evans JR, Cummins G, Breen DP, et al. Comparative epidemiology of incident Parkinson's disease in Cambridgeshire, UK. J Neurol Neurosurg Psychiatry 2016;87(9):1034-1036.
7. Duncan GW, Khoo TK, Coleman SY, et al. The incidence of Parkinson's disease in the North-East of England. Age Ageing 2014;43(2):257-263.
8. Okunoye O, Marston L, Walters K, Schrag A. Change in the incidence of Parkinson's disease in a large UK primary care database. NPJ Parkinsons Dis 2022;8(1):23.
9. Harada H, Nishikawa S, Takahashi K. Epidemiology of Parkinson's disease in a Japanese city. Arch Neurol 1983;40(3):151-154.
10. Morens DM, Davis JW, Grandinetti A, Ross GW, Popper JS, White LR. Epidemiologic observations on Parkinson's disease: incidence and mortality in a prospective study of middle-aged men. Neurology 1996;46(4):1044-1050.
11. Bower JH, Maraganore DM, McDonnell SK, Rocca WA. Incidence and distribution of parkinsonism in Olmsted County, Minnesota, 1976-1990. Neurology 1999;52(6):1214-1220.
12. Baldereschi M, Di Carlo A, Rocca WA, et al. Parkinson's disease and parkinsonism in a longitudinal study: two-fold higher incidence in men. ILSA Working Group. Italian Longitudinal Study on Aging. Neurology 2000;55(9):1358-1363.
13. Morioka S, Sakata K, Yoshida S, et al. Incidence of Parkinson disease in Wakayama, Japan. J Epidemiol 2002;12(6):403-407.
14. Van Den Eeden SK, Tanner CM, Bernstein AL, et al. Incidence of Parkinson's disease: variation by age, gender, and race/ethnicity. Am J Epidemiol 2003;157(11):1015-1022.
15. Twelves D, Perkins KS, Counsell C. Systematic review of incidence studies of Parkinson's disease. Movement disorders : official journal of the Movement Disorder Society 2003;18(1):19-31.
16. de Lau LM, Giesbergen PC, de Rijk MC, Hofman A, Koudstaal PJ, Breteler MM. Incidence of parkinsonism and Parkinson disease in a general population: the Rotterdam Study. Neurology 2004;63(7):1240-1244.
17. Tan LC, Venketasubramanian N, Jamora RD, Heng D. Incidence of Parkinson's disease in Singapore. Parkinsonism Relat Disord 2007;13(1):40-43.
18. Alves G, Müller B, Herlofson K, et al. Incidence of Parkinson's disease in Norway: the Norwegian ParkWest study. J Neurol Neurosurg Psychiatry 2009;80(8):851-857.
19. Perez F, Helmer C, Dartigues JF, Auriacombe S, Tison F. A 15-year population-based cohort study of the incidence of Parkinson's disease and Dementia with Lewy bodies in an elderly French cohort. J Neurol Neurosurg Psychiatry 2010;81(7):742-746.
20. Linder J, Stenlund H, Forsgren L. Incidence of Parkinson's disease and parkinsonism in northern Sweden: a population-based study. Mov Disord 2010;25(3):341-348.
21. Winter Y, Bezdolnyy Y, Katunina E, et al. Incidence of Parkinson's disease and atypical parkinsonism: Russian population-based study. Mov Disord 2010;25(3):349-356.
22. Savica R, Grossardt BR, Bower JH, Ahlskog JE, Rocca WA. Incidence and pathology of synucleinopathies and tauopathies related to parkinsonism. JAMA Neurol 2013;70(7):859-866.
23. Hirsch L, Jette N, Frolkis A, Steeves T, Pringsheim T. The incidence of Parkinson's Disease: a systematic review and meta-analysis. Neuroepidemiology 2016;46(4):292-300.
24. Savica R, Grossardt BR, Bower JH, Ahlskog JE, Rocca WA. Time trends in the incidence of Parkinson disease. JAMA Neurol 2016;73(8):981-989.
25. Darweesh SK, Koudstaal PJ, Stricker BH, Hofman A, Ikram MA. Trends in the incidence of Parkinson disease in the general population: the Rotterdam study. Am J Epidemiol 2016;183(11):1018-1026.

26. Canonico M, Artaud F, Degaey I, et al. Incidence of Parkinson's disease in French women from the E3N cohort study over 27 years of follow-up. Eur J Epidemiol 2022;37(5):513-523.
27. Brakedal B, Toker L, Haugarvoll K, Tzoulis C. A nationwide study of the incidence, prevalence and mortality of Parkinson's disease in the Norwegian population. NPJ Parkinsons Dis 2022;8(1):19.
28. Dammertz L, Schrag A, Bohlken J, et al. Falling incidence of Parkinson's disease in Germany. Eur J Neurol 2023;30(10):3124-3131.
29. Fink A, Pavlou MAS, Roomp K, Schneider JG. Declining trends in the incidence of Parkinson's disease: A cohort study in Germany. J Parkinsons Dis 2024 Feb;15(1):182-188.
30. Brewis M, Poskanzer DC, Rolland C, Miller H. Neurological disease in an English city. Acta Neurol Scand 1966;42:Suppl 24:21-89.
31. Sutcliffe RL, Prior R, Mawby B, McQuillan WJ. Parkinson's disease in the district of the Northampton Health Authority, United Kingdom. A study of prevalence and disability. Acta Neurol Scand 1985;72(4):363-379.
32. Mutch WJ, Dingwall-Fordyce I, Downie AW, Paterson JG, Roy SK. Parkinson's disease in a Scottish city. Br Med J (Clin Res Ed) 1986;292(6519):534-536.
33. Sutcliffe RL, Meara JR. Parkinson's disease epidemiology in the Northampton District, England, 1992. Acta Neurol Scand 1995;92(6):443-450.
34. Schrag A, Ben-Shlomo Y, Quinn NP. Cross sectional prevalence survey of idiopathic Parkinson's disease and parkinsonism in London. Bmj 2000;321(7252):21-22.
35. Hobson P, Gallacher J, Meara J. Cross-sectional survey of Parkinson's disease and parkinsonism in a rural area of the United Kingdom. Mov Disord 2005;20(8):995-998.
36. Porter B, Macfarlane R, Unwin N, Walker R. The prevalence of Parkinson's disease in an area of North Tyneside in the North-East of England. Neuroepidemiology 2006;26(3):156-161.
37. Wickremaratchi MM, Perera D, O'Loghlen C, et al. Prevalence and age of onset of Parkinson's disease in Cardiff: a community based cross sectional study and meta-analysis. J Neurol Neurosurg Psychiatry 2009;80(7):805-807.
38. Newman EJ, Grosset KA, Grosset DG. Geographical difference in Parkinson's disease prevalence within West Scotland. Mov Disord 2009;24(3):401-406.
39. Walker RW, Hand A, Jones C, Wood BH, Gray WK. The prevalence of Parkinson's disease in a rural area of North-East England. Parkinsonism Relat Disord 2010;16(9):572-575.
40. Parkinson's UK. The prevalence and incidence of Parkinson’s in the UK. Results from the Clinical Practice Research Datalink Reference Report. 2017. Available from: https://www.parkinsons.org.uk/sites/default/files/2018-01/CS2960%20Incidence%20and%20prevalence%20report%20branding%20summary%20report%20Published%202017.pdf (accessed 6 June 2024).
41. Li SC, Schoenberg BS, Wang CC, et al. A prevalence survey of Parkinson's disease and other movement disorders in the People's Republic of China. Arch Neurol 1985;42(7):655-657.
42. Bharucha NE, Bharucha EP, Bharucha AE, Bhise AV, Schoenberg BS. Prevalence of Parkinson's disease in the Parsi community of Bombay, India. Arch Neurol 1988;45(12):1321-1323.
43. Schoenberg BS, Osuntokun BO, Adeuja AO, et al. Comparison of the prevalence of Parkinson's disease in black populations in the rural United States and in rural Nigeria: door-to-door community studies. Neurology 1988;38(4):645-646.
44. Granieri E, Carreras M, Casetta I, et al. Parkinson's disease in Ferrara, Italy, 1967 through 1987. Arch Neurol 1991;48(8):854-857.
45. Morgante L, Rocca WA, Di Rosa AE, et al. Prevalence of Parkinson's disease and other types of parkinsonism: a door-to-door survey in three Sicilian municipalities. The Sicilian Neuro-Epidemiologic Study (SNES) Group. Neurology 1992;42(10):1901-1907.
46. Caradoc-Davies TH, Weatherall M, Dixon GS, Caradoc-Davies G, Hantz P. Is the prevalence of Parkinson's disease in New Zealand really changing? Acta Neurol Scand 1992;86(1):40-44.
47. Tison F, Dartigues JF, Dubes L, Zuber M, Alperovitch A, Henry P. Prevalence of Parkinson's disease in the elderly: a population study in Gironde, France. Acta Neurol Scand 1994;90(2):111-115.
48. de Rijk MC, Breteler MM, Graveland GA, et al. Prevalence of Parkinson's disease in the elderly: the Rotterdam Study. Neurology 1995;45(12):2143-2146.
49. Mayeux R, Marder K, Cote LJ, et al. The frequency of idiopathic Parkinson's disease by age, ethnic group, and sex in northern Manhattan, 1988-1993. Am J Epidemiol 1995;142(8):820-827.
50. Tandberg E, Larsen JP, Nessler EG, Riise T, Aarli JA. The epidemiology of Parkinson's disease in the county of Rogaland, Norway. Mov Disord 1995;10(5):541-549.
51. Kusumi M, Nakashima K, Harada H, Nakayama H, Takahashi K. Epidemiology of Parkinson's disease in Yonago City, Japan: comparison with a study carried out 12 years ago. Neuroepidemiology 1996;15(4):201-207.

52. Moriwaka F, Tashiro K, Itoh K, et al. Prevalence of Parkinson's disease in Hokkaido, the northernmost island of Japan. Intern Med 1996;35(4):276-279.
53. Wang SJ, Fuh JL, Teng EL, et al. A door-to-door survey of Parkinson's disease in a Chinese population in Kinmen. Arch Neurol 1996;53(1):66-71.
54. Fall PA, Axelson O, Fredriksson M, et al. Age-standardized incidence and prevalence of Parkinson's disease in a Swedish community. J Clin Epidemiol 1996;49(6):637-641.
55. de Rijk MC, Tzourio C, Breteler MM, et al. Prevalence of parkinsonism and Parkinson's disease in Europe: the EUROPARKINSON Collaborative Study. European Community Concerted Action on the Epidemiology of Parkinson's disease. J Neurol Neurosurg Psychiatry 1997;62(1):10-15.
56. Melcon MO, Anderson DW, Vergara RH, Rocca WA. Prevalence of Parkinson's disease in Junín, Buenos Aires Province, Argentina. Mov Disord 1997;12(2):197-205.
57. Wermuth L, Joensen P, Bünger N, Jeune B. High prevalence of Parkinson's disease in the Faroe Islands. Neurology 1997;49(2):426-432.
58. Chiò A, Magnani C, Schiffer D. Prevalence of Parkinson's disease in Northwestern Italy: comparison of tracer methodology and clinical ascertainment of cases. Mov Disord 1998;13(3):400-405.
59. Errea JM, Ara JR, Aibar C, de Pedro-Cuesta J. Prevalence of Parkinson's disease in lower Aragon, Spain. Mov Disord 1999;14(4):596-604.
60. Kuopio AM, Marttila RJ, Helenius H, Rinne UK. Changing epidemiology of Parkinson's disease in southwestern Finland. Neurology 1999;52(2):302-308.
61. Wermuth L, von Weitzel-Mudersbach P, Jeune B. A two-fold difference in the age-adjusted prevalences of Parkinson's disease between the island of Als and the Faroe Islands. Eur J Neurol 2000;7(6):655-660.
62. Chen RC, Chang SF, Su CL, et al. Prevalence, incidence, and mortality of PD: a door-to-door survey in Ilan county, Taiwan. Neurology 2001;57(9):1679-1686.
63. Milanov I, Kmetska K, Karakolev B, Nedialkov E. Prevalence of Parkinson's disease in Bulgaria. Neuroepidemiology 2001;20(3):212-214.
64. Wermuth L, Pakkenberg H, Jeune B. High age-adjusted prevalence of Parkinson's disease among Inuits in Greenland. Neurology 2002;58(9):1422-1425.
65. Kis B, Schrag A, Ben-Shlomo Y, et al. Novel three-stage ascertainment method: prevalence of PD and parkinsonism in South Tyrol, Italy. Neurology 2002;58(12):1820-1825.
66. Clavería LE, Duarte J, Sevillano MD, et al. Prevalence of Parkinson's disease in Cantalejo, Spain: a door-to-door survey. Mov Disord 2002;17(2):242-249.
67. Anca M, Paleacu D, Shabtai H, Giladi N. Cross-sectional study of the prevalence of Parkinson's disease in the Kibbutz movement in Israel. Neuroepidemiology 2002;21(1):50-55.
68. Taba P, Asser T. Prevalence of Parkinson's disease in Estonia. Acta Neurol Scand 2002;106(5):276-281.
69. Kimura H, Kurimura M, Wada M, et al. Female preponderance of Parkinson's disease in Japan. Neuroepidemiology 2002;21(6):292-296.
70. Nicoletti A, Sofia V, Bartoloni A, et al. Prevalence of Parkinson's disease: a door-to-door survey in rural Bolivia. Parkinsonism Relat Disord 2003;10(1):19-21.
71. Benito-León J, Bermejo-Pareja F, Rodríguez J, Molina JA, Gabriel R, Morales JM. Prevalence of PD and other types of parkinsonism in three elderly populations of central Spain. Mov Disord 2003;18(3):267-274.
72. Zhang ZX, Anderson DW, Huang JB, et al. Prevalence of Parkinson's disease and related disorders in the elderly population of greater Beijing, China. Mov Disord 2003;18(7):764-772.
73. Bergareche A, De La Puente E, López de Munain A, et al. Prevalence of Parkinson's disease and other types of Parkinsonism. A door-to-door survey in Bidasoa, Spain. J Neurol 2004;251(3):340-345.
74. Tan LC, Venketasubramanian N, Hong CY, et al. Prevalence of Parkinson disease in Singapore: Chinese vs Malays vs Indians. Neurology 2004;62(11):1999-2004.
75. Sánchez JL, Buriticá O, Pineda D, Uribe CS, Palacio LG. Prevalence of Parkinson's disease and parkinsonism in a Colombian population using the capture-recapture method. Int J Neurosci 2004;114(2):175-182.
76. Totaro R, Marini C, Pistoia F, Sacco S, Russo T, Carolei A. Prevalence of Parkinson's disease in the L'Aquila district, central Italy. Acta Neurol Scand 2005;112(1):24-28.
77. Chan DK, Cordato D, Karr M, et al. Prevalence of Parkinson's disease in Sydney. Acta Neurol Scand 2005;111(1):7-11.
78. Zhang ZX, Roman GC, Hong Z, et al. Parkinson's disease in China: prevalence in Beijing, Xian, and Shanghai. Lancet 2005;365(9459):595-597.
79. Zhang L, Nie ZY, Liu Y, et al. The prevalence of PD in a nutritionally deficient rural population in China. Acta Neurol Scand 2005;112(1):29-35.

80. von Campenhausen S, Bornschein B, Wick R, et al. Prevalence and incidence of Parkinson's disease in Europe. Eur Neuropsychopharmacol 2005;15(4):473-490.
81. Okubadejo NU, Bower JH, Rocca WA, Maraganore DM. Parkinson's disease in Africa: A systematic review of epidemiologic and genetic studies. Mov Disord 2006;21(12):2150-2156.
82. Barbosa MT, Caramelli P, Maia DP, et al. Parkinsonism and Parkinson's disease in the elderly: a community-based survey in Brazil (the Bambuí study). Mov Disord 2006;21(6):800-808.
83. Mehta P, Kifley A, Wang JJ, Rochtchina E, Mitchell P, Sue CM. Population prevalence and incidence of Parkinson's disease in an Australian community. Intern Med J 2007;37(12):812-814.
84. Wermuth L, Bech S, Petersen MS, Joensen P, Weihe P, Grandjean P. Prevalence and incidence of Parkinson's disease in The Faroe Islands. Acta Neurol Scand 2008;118(2):126-131.
85. Dotchin C, Msuya O, Kissima J, et al. The prevalence of Parkinson's disease in rural Tanzania. Mov Disord 2008;23(11):1567-1672.
86. Morgante L, Nicoletti A, Epifanio A, et al. Prevalence of Parkinson's disease and other types of parkinsonism in the Aeolian Archipelago, Sicily. Parkinsonism Relat Disord 2008;14(7):572-575.
87. Alrefai A, Habahbih M, Alkhawajah M, et al. Prevalence of Parkinson's disease in Northern Jordan. Clin Neurol Neurosurg 2009;111(10):812-815.
88. Yamawaki M, Kusumi M, Kowa H, Nakashima K. Changes in prevalence and incidence of Parkinson's disease in Japan during a quarter of a century. Neuroepidemiology 2009;32(4):263-269.
89. Chen CC, Chen TF, Hwang YC, et al. Different prevalence rates of Parkinson's disease in urban and rural areas: a population-based study in Taiwan. Neuroepidemiology 2009;33(4):350-357.
90. Muangpaisan W, Hori H, Brayne C. Systematic review of the prevalence and incidence of Parkinson's disease in Asia. J Epidemiol 2009;19(6):281-293.
91. Lix LM, Hobson DE, Azimaee M, Leslie WD, Burchill C, Hobson S. Socioeconomic variations in the prevalence and incidence of Parkinson's disease: a population-based analysis. J Epidemiol Community Health 2010;64(4):335-340.
92. Masalha R, Kordysh E, Alpert G, et al. The prevalence of Parkinson's disease in an Arab population, Wadi Ara, Israel. Isr Med Assoc J 2010;12(1):32-35.
93. Das SK, Misra AK, Ray BK, et al. Epidemiology of Parkinson disease in the city of Kolkata, India: a community-based study. Neurology 2010;75(15):1362-1369.
94. Wright Willis A, Evanoff BA, Lian M, Criswell SR, Racette BA. Geographic and ethnic variation in Parkinson disease: a population-based study of US Medicare beneficiaries. Neuroepidemiology 2010;34(3):143-151.
95. Osaki Y, Morita Y, Kuwahara T, Miyano I, Doi Y. Prevalence of Parkinson's disease and atypical parkinsonian syndromes in a rural Japanese district. Acta Neurol Scand 2011;124(3):182-187.
96. Seijo-Martinez M, Castro del Rio M, Rodríguez Alvarez J, et al. Prevalence of parkinsonism and Parkinson's disease in the Arosa Island (Spain): a community-based door-to-door survey. J Neurol Sci 2011;304(1-2):49-54.
97. Khedr EM, Al Attar GS, Kandil MR, Kamel NF, Abo Elfetoh N, Ahmed MA. Epidemiological study and clinical profile of Parkinson's disease in the Assiut Governorate, Egypt: a community-based study. Neuroepidemiology 2012;38(3):154-163.
98. Gordon PH, Mehal JM, Holman RC, Rowland AS, Cheek JE. Parkinson's disease among American Indians and Alaska natives: a nationwide prevalence study. Mov Disord 2012;27(11):1456-1459.
99. Gordon PH, Zhao H, Bartley D, et al. Prevalence of Parkinson disease among the Navajo: a preliminary examination. J Parkinsons Dis 2013;3(2):193-198.
100. El-Tallawy HN, Farghaly WM, Shehata GA, et al. Prevalence of Parkinson's disease and other types of Parkinsonism in Al Kharga district, Egypt. Neuropsychiatr Dis Treat 2013;9:1821-1826.
101. Blanckenberg J, Bardien S, Glanzmann B, Okubadejo NU, Carr JA. The prevalence and genetics of Parkinson's disease in sub-Saharan Africans. J Neurol Sci 2013;335(1-2):22-25.
102. Pringsheim T, Jette N, Frolkis A, Steeves TD. The prevalence of Parkinson's disease: a systematic review and meta-analysis. Mov Disord 2014;29(13):1583-1590.
103. Blin P, Dureau-Pournin C, Foubert-Samier A, et al. Parkinson's disease incidence and prevalence assessment in France using the national healthcare insurance database. Eur J Neurol 2015;22(3):464-471.
104. Gordon PH, Mehal JM, Holman RC, Bartholomew ML, Cheek JE, Rowland AS. Incidence and prevalence of Parkinson's disease among Navajo people living in the Navajo nation. Mov Disord 2015;30(5):714-720.
105. Khedr EM, Fawi G, Abbas MA, et al. Prevalence of Parkinsonism and Parkinson's disease in Qena governorate/Egypt: a cross-sectional community-based survey. Neurol Res 2015;37(7):607-618.
106. Yang XL, Luo Q, Song HX, Wang YL, Yao YN, Xia H. Related factors and prevalence of Parkinson's disease among Uygur residents in Hetian, Xinjiang Uygur Autonomous Region. Genet Mol Res 2015;14(3):8539-8546.

107. Zou YM, Liu J, Tian ZY, Lu D, Zhou YY. Systematic review of the prevalence and incidence of Parkinson's disease in the People's Republic of China. Neuropsychiatr Dis Treat 2015;11:1467-1472.
108. Liu CC, Li CY, Lee PC, Sun Y. Variations in incidence and prevalence of Parkinson's disease in Taiwan: a population-based nationwide study. Parkinsons Dis 2016;2016:8756359.
109. GBD 2016 Parkinson's Disease Collaborators. Global, regional, and national burden of Parkinson's disease, 1990-2016: a systematic analysis for the Global Burden of Disease Study 2016. Lancet Neurol 2018;17(11):939-953.
110. Muangpaisan W, Siritipakorn P, Assantachai P. Development of a Thai Parkinson's disease screening tool and the prevalence of parkinsonism and Parkinson's disease, based on a community survey in Bangkok. Neuroepidemiology 2017;49(1-2):74-81.
111. Abbas MM, Xu Z, Tan LCS. Epidemiology of Parkinson's disease-East versus West. Mov Disord Clin Pract 2018;5(1):14-28.
112. Williams U, Bandmann O, Walker R. Parkinson's Disease in Sub-Saharan Africa: A Review of Epidemiology, Genetics and Access to Care. J Mov Disord 2018;11(2):53-64.
113. Fleury V, Brindel P, Nicastro N, Burkhard PR. Descriptive epidemiology of parkinsonism in the Canton of Geneva, Switzerland. Parkinsonism Relat Disord 2018;54:30-39.
114. Kadastik-Eerme L, Taba N, Asser T, Taba P. The increasing prevalence of Parkinson's disease in Estonia. Acta Neurol Scand 2018;138(3):251-258.
115. Valent F, Devigili G, Rinaldo S, Del Zotto S, Tullio A, Eleopra R. The epidemiology of Parkinson's disease in the Italian region Friuli Venezia Giulia: a population-based study with administrative data. Neurol Sci 2018;39(4):699-704.
116. Szatmári Jr S, Ajtay A, Bálint M, Takáts A, Oberfrank F, Bereczki D. Linking individual patient data to estimate incidence and prevalence of Parkinson's disease by comparing reports of neurological services and pharmacy prescription Refills at a nationwide level. Front Neurol 2019;10:640.
117. Park JH, Kim DH, Kwon DY, et al. Trends in the incidence and prevalence of Parkinson's disease in Korea: a nationwide, population-based study. BMC Geriatr 2019;19(1):320.
118. Han S, Kim S, Kim H, Shin HW, Na KS, Suh HS. Prevalence and incidence of Parkinson's disease and drug-induced parkinsonism in Korea. BMC Public Health 2019;19(1):1328.
119. Institute for Health Metrics and Evaluation (IfHMaE). Global Burden of Disease Study 2021 Results. Available from: http://ghdx.healthdata.org/gbd-results-tool (accessed 3 January 2025).
120. Qi S, Yin P, Wang L, et al. Prevalence of Parkinson's disease: a community-based study in China. Mov Disord 2021;36(12):2940-2944.
121. Song Z, Liu S, Li X, et al. Prevalence of Parkinson's disease in adults aged 65 years and older in China: a multicenter population-based survey. Neuroepidemiology 2022;56(1):50-58.
122. Cicero CE, Scondotto S, Allotta AV, et al. Burden of Parkinson's disease in Sicily: a health administrative database study. Neurol Sci 2022;43(2):1043-1046.
123. Llibre-Guerra JJ, Prina M, Sosa AL, et al. Prevalence of parkinsonism and Parkinson disease in urban and rural populations from Latin America: a community based study. Lancet Reg Health Am 2022;7:None.
124. Varden R, Walker R, O'Callaghan A. No trend to rising rates: A review of Parkinson's prevalence studies in the United Kingdom. Parkinsonism Relat Disord. 2024 Nov;128:107015. doi: 10.1016/j.parkreldis.2024.107015. Epub 2024 May 20. PMID: 38876845.
125. Zhu J, Cui Y, Zhang J, et al. Temporal trends in the prevalence of Parkinson's disease from 1980 to 2023: a systematic review and meta-analysis. Lancet Healthy Longev 2024;5(7):e464-e479.
126. Kim DJ, Isidro-Pérez AL, Doering M, et al. Prevalence and incidence of Parkinson's disease in Latin America: a meta-analysis. Mov Disord 2024;39(1):105-118.
127. Bower JH, Maraganore DM, McDonnell SK, Rocca WA. Incidence of progressive supranuclear palsy and multiple system atrophy in Olmsted County, Minnesota, 1976 to 1990. Neurology 1997;49(5):1284-1288.
128. Schrag A, Ben-Shlomo Y, Quinn NP. Prevalence of progressive supranuclear palsy and multiple system atrophy: a cross-sectional study. Lancet 1999;354(9192):1771-1775.
129. Kawashima M, Miyake M, Kusumi M, Adachi Y, Nakashima K. Prevalence of progressive supranuclear palsy in Yonago, Japan. Mov Disord 2004;19(10):1239-1240.
130. Wada-Isoe K, Uemura Y, Suto Y, et al. Prevalence of dementia in the rural island town of Ama-cho, Japan. Neuroepidemiology 2009;32(2):101-106.
131. Vann Jones SA, O'Brien JT. The prevalence and incidence of dementia with Lewy bodies: a systematic review of population and clinical studies. Psychol Med 2014;44(4):673-683.
132. Hogan DB, Fiest KM, Roberts JI, et al. The prevalence and incidence of Dementia with Lewy Bodies: a systematic review. Can J Neurol Sci 2016;43 Suppl 1:S83-95.
133. Takigawa H, Kitayama M, Wada-Isoe K, Kowa H, Nakashima K. Prevalence of progressive supranuclear palsy in Yonago: change throughout a decade. Brain Behav 2016;6(12):e00557.

134. Savica R, Grossardt BR, Bower JH, Ahlskog JE, Mielke MM, Rocca WA. Incidence and time trends of drug-induced parkinsonism: a 30-year population-based study. Mov Disord 2017;32(2):227-234.
135. Stang CD, Turcano P, Mielke MM, et al. Incidence and trends of Progressive Supranuclear Palsy and Corticobasal Syndrome: a population-based study. J Parkinsons Dis 2020;10(1):179-184.
136. Swallow DMA, Zheng CS, Counsell CE. Systematic review of prevalence studies of Progressive Supranuclear Palsy and Corticobasal Syndrome. Mov Disord Clin Pract 2022;9(5):604-613.
137. Swallow DMA, Counsell CE. Prevalence of Progressive Supranuclear Palsy and Corticobasal Syndrome in Scotland. Neuroepidemiology 2022;56(4):291-297.
138. Lyons S, Trépel D, Lynch T, Walsh R, O'Dowd S. The prevalence and incidence of progressive supranuclear palsy and corticobasal syndrome: a systematic review and meta-analysis. J Neurol 2023;270(9):4451-4465.
139. Logroscino G, Piccininni M, Graff C, et al. Incidence of syndromes associated with frontotemporal lobar degeneration in 9 european countries. JAMA Neurol 2023;80(3):279-286.
140. Kaplan S. Prevalence of multiple system atrophy: A literature review. Rev Neurol (Paris) 2024;180(5):438-450.
