# Supplementary material for: Comparative Analysis of the Incidence, Prevalence, and Survival of 8 Types of Parkinsonism in a Population‐Based Study with 367 Million Person Years of Observation over 21 Years
Source: Mov Disord Clin Pract. 2025 Oct 22;13(4):933–48. doi: 10.1002/mdc3.70368 (PMC13071333; doi:10.1002/mdc3.70368)
Supplement: Supplementary file 3 — TABLE S2. Diagnostic codes used for case definitions of eight types of parkinsonism. Case definitions for the eight parkinsonian disorders were based on diagnostic Read and SNOMED codes in CPRD, and International Classification of Diseases tenth revision (ICD‐10) codes in HES and ONS. TABLE S3. Demographic and clinical features of coded and imputed PD cases. Coded and imputed cases of Parkinson's disease were compared by t‐test (numerical data) or Chi‐squared tests (categorical data). Imputed incident cases were older than coded cases, but imputed prevalent cases were younger and had a shorter treatment duration. TABLE S4. Summary of diagnostic sequence for incident cases of degenerative parkinsonism, 2003 to 2023. From 2003 to 2023, diagnoses were revised for 6.1% of incident cases with degenerative parkinsonism and for 4.6% of incident cases of Parkinson's disease. TABLE S5. Summary of case counts for Kaplan–Meier graphs of survival shown in Figure 4. The population at risk and number of deaths, corresponding to the Kaplan–Meier survival curves in Figure 4, are presented for cases and controls for each parkinsonian disorder over the 21‐years of follow‐up. TABLE S6. Survival after a diagnosis of parkinsonism compared to matched controls. For each type of parkinsonism, median, 5‐ and 10‐year percentage survival were reduced for cases compared to their matched controls. Among the degenerative parkinsonisms, median and 5‐year survival were longest for Parkinson's disease and shortest for Progressive Supranuclear Palsy. TABLE S7. Life expectancy estimates for eight types of parkinsonism by age and sex. Life expectancy values are at the start of the age interval. For all parkinsonian disorders, life expectancy was reduced for cases than controls. Greater differences in life expectancy between cases and controls were observed for younger than older onset cases. TABLE S8. Extended Cox model of survival in Parkinson's cases versus matched controls. Hazard ratios are adjust [file MDC3-13-933-s004.pdf]

Supplementary Table 2. Diagnostic codes used for case definitions of eight types of parkinsonism.

Parkinson's disease

Code Type	Diagnostic code	Description
Medcode (Gold)	101090	History of Parkinson's disease
Medcode (Gold)	96860	Cerebral degeneration in Parkinson's disease
Medcode (Gold)	4321	Parkinson's disease
Medcode (Gold)	9509	[X]Dementia in Parkinson's disease
Medcode (Gold)	14912	Parkinson's disease NOS
Medcode (Gold)	1691	Paralysis agitans
MedcodeID (Aurum)	81717011	Parkinson's disease
MedcodeID (Aurum)	295688010	Dementia in Parkinsons disease
MedcodeID (Aurum)	297037012	Parkinson's disease NOS
MedcodeID (Aurum)	297043014	Orthostatic hypotension co-occurrent and due to Parkinson's disease
MedcodeID (Aurum)	905821000006112	[RFC] Parkinson's disease
MedcodeID (Aurum)	909021000006117	[RFC] Parkinson's disease
MedcodeID (Aurum)	1148731000000112	History of Parkinson's disease
MedcodeID (Aurum)	1776901000006117	Reason for referral: Parkinsons Disease
MedcodeID (Aurum)	3293631000006118	Idiopathic Parkinson's disease
MedcodeID (Aurum)	3293641000006111	Parkinson disease
MedcodeID (Aurum)	3293651000006113	PD - Parkinson's disease
MedcodeID (Aurum)	3293661000006110	Parkinsons disease
MedcodeID (Aurum)	3293681000006117	Idiopathic Parkinsonism
MedcodeID (Aurum)	7043651000006119	Dementia associated with Parkinson's Disease
MedcodeID (Aurum)	7043661000006117	Dementia associated with Parkinson Disease
MedcodeID (Aurum)	7730791000006112	Young onset Parkinson disease
MedcodeID (Aurum)	7730801000006113	Young onset Parkinson's disease
MedcodeID (Aurum)	7730811000006111	Early onset Parkinson disease
MedcodeID (Aurum)	7730821000006115	Early onset Parkinson's disease
MedcodeID (Aurum)	7790241000006117	Psychosis co-occurrent and due to Parkinson's disease
MedcodeID (Aurum)	8031751000006119	Dementia due to Parkinson's disease
MedcodeID (Aurum)	8031761000006117	Dementia due to Parkinsons disease
MedcodeID (Aurum)	8031771000006112	Dementia due to Parkinson disease
MedcodeID (Aurum)	81719014	Paralysis agitans
ICD 10	G20	Parkinson's disease
ICD 10	F02.3	Parkinson's disease

Multiple system atrophy

Code Type	Diagnostic code	Description
Medcode (Gold)	35839	Shy-Drager syndrome
Medcode (Gold)	22454	Multiple system atrophy
MedcodeID (Aurum)	2841061014	Multiple system atrophy, Parkinson variant
MedcodeID (Aurum)	7289421000006111	Multiple system atrophy, Parkinson's variant
MedcodeID (Aurum)	5005991000006114	MSA - Multiple system atrophy

MedcodeID (Aurum)	28047015	Shy-Drager syndrome
MedcodeID (Aurum)	345122012	Multiple system atrophy
MedcodeID (Aurum)	2838078010	Multiple system atrophy, cerebellar variant
MedcodeID (Aurum)	1504174100006111	Dementia due to multiple system atrophy
MedcodeID (Aurum)	49542018	Striatonigral degeneration
ICD 10	G23.2	Multiple System Atrophy
ICD 10	G23.3	Multiple System Atrophy

Progressive supranuclear palsy

Code Type	Diagnostic code	Description
Medcode (Gold)	9385	Progressive supranuclear palsy
Medcode (Gold)	40553	Progressive supranuclear ophthalmoplegia
Medcode (Gold)	93910	Steele - Richardson Oszewski syndrome
Medcode (Gold)	49034	Steele Richardson Olszewsk syn
Medcode (Gold)	7037	Steele-Richardson-Olszewski syndrome
MedcodeID (Aurum)	1446399100006112	Progressive supranuclear palsy parkinsonism syndrome
MedcodeID (Aurum)	48515010	Steele-Richardson-Olszewski syndrome
MedcodeID (Aurum)	125891000006119	Steele - Richardson Oszewski syndrome
MedcodeID (Aurum)	125901000006115	Steele Richardson Olszewsk syn
MedcodeID (Aurum)	48512013	Progressive supranuclear ophthalmoplegia
MedcodeID (Aurum)	48513015	Progressive supranuclear palsy
MedcodeID (Aurum)	297258013	Progressive supranuclear palsy
MedcodeID (Aurum)	3511377010	PSP - progressive supranuclear palsy
ICD 10	G23.1	Progressive Supranuclear Palsy

Corticobasal syndrome

Code Type	Diagnostic code	Description
Medcode (Gold)	107650	Corticobasal degeneration
MedcodeID (Aurum)	31772014	Corticobasal degeneration
ICD 10	G23.9	Corticobasal syndrome

Dementia with Lewy bodies

Code Type	Diagnostic code	Description
Medcode (Gold)	26270	[X]Lewy body dementia
Medcode (Gold)	7572	Lewy body disease
MedcodeID (Aurum)	299641000000112	Diffuse Lewy body disease
MedcodeID (Aurum)	745381000006119	Lewy body disease
MedcodeID (Aurum)	914931000006119	[D] Dementia with Lewy bodies
MedcodeID (Aurum)	3189531000006118	Diffuse Lewy body disease with spongiform cortical change
MedcodeID (Aurum)	3802641000006114	LBD - Lewy body disease
MedcodeID (Aurum)	3802651000006111	Dementia of the Lewy body type
MedcodeID (Aurum)	3802671000006118	Cortical Lewy body disease
MedcodeID (Aurum)	6024061000006111	Senile dementia of the Lewy body type
MedcodeID (Aurum)	6024071000006116	Lewy body dementia
MedcodeID (Aurum)	8042791000006116	Lewy body dementia with behavioural disturbance

MedcodeID (Aurum)	8042801000006115	Lewy body dementia with behavioral disturbance
MedcodeID (Aurum)	13810191000006114	SDLT - senile dementia of Lewy body type

Vascular parkinsonism

Code Type	Diagnostic code	Description
Medcode (Gold)	100128	Vascular parkinsonism
MedcodeID (Aurum)	345121017	Vascular parkinsonism
ICD 10	G21.4	Vascular Parkinsonism

Drug-induced parkinsonism

Code Type	Diagnostic code	Description
Medcode (Gold)	19478	Drug induced parkinsonism
Medcode (Gold)	33544	Parkinsonism secondary to drugs
Medcode (Gold)	105947	[X]Other drug-induced secondary parkinsonism
MedcodeID (Aurum)	299312017	[X]Other drug-induced secondary parkinsonism
MedcodeID (Aurum)	244311000006117	Parkinsonism secondary to drugs
MedcodeID (Aurum)	630491000006110	Parkinsonism due to drug
MedcodeID (Aurum)	2566251000006117	Parkinsonism caused by drug
MedcodeID (Aurum)	3684041000006116	Neuroleptic-induced Parkinsonism
MedcodeID (Aurum)	3684051000006119	Neuroleptic-induced parkinsonism
ICD 10	G21.1	Drug-induced parkinsonism

Other secondary parkinsonism

Code Type	Diagnostic code	Description
Medcode (Gold)	86062	[X]Parkinsonism in diseases classified elsewhere
Medcode (Gold)	51105	Postencephalitic parkinsonism
Medcode (Gold)	72879	[X]Secondary parkinsonism, unspecified
Medcode (Gold)	97170	[X]Other secondary parkinsonism
Medcode (Gold)	26181	Secondary parkinsonism, unspecified
Medcode (Gold)	52589	Syphilitic parkinsonism
Medcode (Gold)	114440	[X]Secondary parkinsonism due to other external agents
Medcode (Gold)	24001	Secondary parkinsonism due to other external agents
MedcodeID (Aurum)	33622011	Postencephalitic parkinsonism
MedcodeID (Aurum)	64875013	Syphilitic parkinsonism
MedcodeID (Aurum)	299313010	[X]Other secondary parkinsonism
MedcodeID (Aurum)	299314016	[X]Parkinsonism in diseases classified elsewhere
MedcodeID (Aurum)	299321016	[X]Secondary parkinsonism, unspecified
MedcodeID (Aurum)	299323018	[X]Secondary parkinsonism due to other external agents
MedcodeID (Aurum)	151951000006115	Secondary parkinsonism due to other external agents
MedcodeID (Aurum)	151961000006118	Secondary parkinsonism
MedcodeID (Aurum)	882961000006112	Secondary Parkinsonism – drugs
MedcodeID (Aurum)	3511181000006115	Parkinson-dementia complex of Guam
MedcodeID (Aurum)	5005931000006110	Secondary Parkinson disease
MedcodeID (Aurum)	5005941000006117	Carbon monoxide-induced parkinsonism
MedcodeID (Aurum)	5005961000006118	Parkinsonism with calcification of basal ganglia

MedcodeID (Aurum)	7558011000006119	Rapid onset dystonia parkinsonism
ICD 10	G21.2	Secondary Parkinsonism
ICD 10	G22	Secondary Parkinsonism
ICD 10	G21.0	Secondary Parkinsonism
ICD 10	G21.9	Secondary Parkinsonism
ICD 10	G21.8	Secondary Parkinsonism
ICD 10	G21.3	Secondary Parkinsonism

Supplementary Table 3: Demographic and clinical features of coded and imputed PD cases

	Incident Parkinson's cases			Prevalent Parkinson's cases		
	**Diagnosis by diagnostic code**	**Diagnosis by therapy imputation**	**p value***	**Diagnosis by diagnostic code**	**Diagnosis by therapy imputation**	**p value***
Number of cases	4 177	738	NA	35 580	2 964	NA
Age, mean (SD)	74.4 (9.5)	76.3 (8.3)	<0.0001	75.4 (10.2)	73.4 (13.2)	<0.0001
BMI, mean (SD)	26.8 (5.0)	26.8 (5.3)	0.79	26.3 (5.6)	26.7 (5.8)	0.0015
Treatment duration, mean (SD)	NA	NA	NA	5.7 (5.0)	2.4 (4.0)	<0.0001
Sex, male (%)	2 713 (64.9%)	492 (66.7%)	0.37	20 911 (58.7%)	1 708 (57.6%)	0.23
Smoking status						
Current, n (%)	384 (9.2%)	79 (10.7%)	0.22	3 571 (10.0%)	352 (11.8%)	0.001
Former, n (%)	2 637 (63.1%)	475 (64.3%)	0.58	21 149 (59.4%)	1 741 (58.7%)	0.47
Never, n (%)	1 135 (27.2%)	179 (24.3%)	0.12	10 493 (29.4%)	821 (27.7%)	0.041
Missing, n (%)	21 (0.5%)	5 (0.6%)	0.74	367 (1.0%)	50 (1.7%)	0.001
BMI, kg / m2						
Mean (SD)	26.8 (5.0)	26.8 (5.3)	0.79	26.3 (5.6)	26.7 (5.8)	0.0015
Missing, n (%)	137 (3.2%)	22 (3.0%)	0.75	1 150 (3.2%)	157 (5.2%)	<0.0001

*Compared by t-test (numerical data) or Chi-squared (categorical data).

NA=not applicable

Supplementary Table 4: Summary of diagnostic sequence for incident cases of degenerative parkinsonism, 2003 to 2023

		Diagnostic revision, n					
Initial diagnosis, n		Parkinson's Disease	Multiple System Atrophy	Progressive Supranuclear Palsy	Corticobasal Syndrome	Dementia with Lewy Bodies	% with change in diagnosis
Parkinson's Disease	125 403	-	952	1409	139	3357	4.6
Multiple System Atrophy	1 503	271	-	30	7	21	21.9
Progressive Supranuclear Palsy	2 510	473	11	-	12	11	20.2
Corticobasal Syndrome	507	79	4	23	-	7	22.3
Dementia with Lewy Bodies	10 645	1 770	10	24	10	-	17.0
All cases	140 568	2 593	977	1 486	168	3 396	6.1

Supplementary Table 5. Summary of case counts for Kaplan Meier graphs of survival shown in Figure 4

			Time from index date (years)							
**Diagnosis**	**Type**		**0**	**3**	**6**	**9**	**12**	**15**	**18**	**21**
**Parkinson's Disease**	Control	At risk	441 788	333 086	230 573	150 583	90 741	45 695	9 839	447
		Events	0	61 706	100 388	123 913	137 018	143 980	146 998	147 780
	Case	At risk	220 930	142 201	88 268	51 546	27 548	12 406	2 065	75
		Events	0	50 267	78 652	95 273	104 298	108 426	109 905	110 147
**Multiple System Atrophy**	Control	At risk	7 960	5 772	3 555	2 057	1 101	482	114	5
		Events	0	603	957	1 158	1 273	1 329	1 350	1 357
	Case	At risk	3 980	1 859	869	470	216	82	6	0
		Events	0	1 311	1 733	1 851	1 898	1 914	1 919	1 920
**Progressive Supranuclear Palsy**	Control	At risk	14 163	10 137	6 225	3 529	1 889	798	170	12
		Events	0	1 433	2 355	2 869	3 145	3 304	3 366	3 379
	Case	At risk	7 082	2 374	997	586	325	134	17	1
		Events	0	3 379	4 289	4 456	4 502	4 515	4 521	4 521
**Corticobasal Syndrome**	Control	At risk	1833	1176	664	315	156	71	16	0
		Event	0	150	261	318	334	339	340	341
	Case	At risk	917	373	160	84	51	21	3	0
		Event	0	285	383	409	415	420	421	421
**Dementia with Lewy Bodies**	Control	At risk	43 768	29 614	18 774	10 742	5 283	2 013	314	8
		Events	0	6 970	10 990	13 275	14 440	14 919	15 078	15 103
	Case	At risk	21 887	10 001	4 009	1 778	797	303	32	0
		Events	0	7 882	11 991	13 266	13 547	13 599	13 605	13 606
**Vascular Parkinsonism**	Control	At risk	11 862	7 538	3 503	1 044	157	10	2	.
		Event	0	2 362	3 513	4 024	4 150	4 171	4 171	.
	Case	At risk	5 933	2 394	881	197	20	4	0	.
		Event	0	2 287	2 897	3 067	3 106	3 108	3 109	.
**Drug-induced parkinsonism**	Control	At risk	18 294	14 405	9 671	6 090	3 676	1 794	356	13

		Event	0	2 085	3 333	4 164	4 593	4 806	4 913	4 923
	Case	At risk	9 148	5 688	3 574	2 212	1 297	596	100	1
		Event	0	2 008	2 813	3 178	3 362	3 456	3 493	3 500
**Other secondary parkinsonism**	Control	At risk	8 582	7 057	4 605	2 701	1 526	768	184	12
		Event	0	919	1 566	1 904	2 101	2 180	2 221	2 231
	Case	At risk	4 292	2 687	1 536	868	482	230	55	2
		Event	0	1 128	1 581	1 762	1 841	1 860	1 866	1 870

Supplementary Table 6: Survival after a diagnosis of parkinsonism compared to matched controls

		Percentage survival	
	Median survival (95% CI)	5 years	10 years
Parkinson's disease	8.1 (8.0, 8.1)	64.2 (64.0, 64.4)	42.7 (42.5, 43.0)
matched controls	16.7 (16.6, 16.8)	77.7 (77.6, 77.8)	63.2 (63.0, 63.4)
Multiple System Atrophy	5.0 (4.5, 5.4)	50.0 (48.2, 51.8)	36.4 (34.4, 38.5)
matched controls	> 21.0 (20.1, >21.0)	87.1 (86.3, 88.0)	77.2 (76.0, 78.5)
Progressive Supranuclear Palsy	2.6 (2.6, 2.8)	30.5 (29.3, 31.8)	20.5 (19.3, 21.7)
matched controls	17.6 (17.1, 19.1)	82.5 (81.8, 83.2)	69.1 (68.1, 70.1)
Corticobasal Syndrome	4.7 (4.3, 5.6)	48.7 (44.9, 52.8)	33.4 (29.1, 38.2)
matched controls	>21.0 (18.0, >21.0)	84.1 (82.1, 86.1)	69.0 (65.8, 72.3)
Dementia with Lewy Bodies	3.8 (3.7, 3.9)	38.4 (37.6, 39.1)	18.6 (18.0, 19.3)
matched controls	12.7 (12.4, 13.0)	73.9 (73.4, 74.3)	56.9 (56.3, 57.5)
Vascular parkinsonism	3.9 (3.7, 4.1)	43.4 (41.9, 44.9)	25.2 (23.1, 27.4)
matched controls	9.0 (8.7, 9.4)	67.7 (66.8, 68.6)	46.4 (44.9, 48.0)
Drug-induced parkinsonism	12.5 (11.7, 13.2)	67.7 (66.7, 68.8)	54.2 (53.0, 55.5)
matched controls	>21.0 (19.4, >21.0)	82.0 (81.4, 82.6)	69.2 (68.4, 70.0)
Other secondary parkinsonism	9.1 (8.4, 9.9)	61.6 (60.1, 63.2)	48.1 (46.2, 49.9)
matched controls	20.3 (18.9, >21.0)	82.7 (81.9, 83.5)	70.4 (69.2, 71.5)

Supplementary Table 7: Life expectancy estimates for eight types of parkinsonism by age and sex

A. Parkinson's disease - All				Matched controls - All		
Age band	Life expectancy	Lower CI	Upper CI	Life expectancy	Lower CI	Upper CI
20 – 24	45.9	45.1	46.8	58.9	58.5	59.2
25 – 29	41.2	40.5	42.0	54.1	53.8	54.3
30 – 34	37.2	36.6	37.7	49.1	48.9	49.3
35 – 39	32.9	32.5	33.3	44.2	44.0	44.4
40 – 44	28.9	28.6	29.2	39.4	39.2	39.5
45 – 49	24.9	24.7	25.1	34.8	34.6	34.9
50 – 54	21.0	20.9	21.1	30.3	30.2	30.4
55 – 59	17.3	17.2	17.4	26.0	25.9	26.1
60 – 64	14.3	14.3	14.4	22.0	21.9	22.1
65 – 69	11.7	11.7	11.8	18.3	18.2	18.3
70 – 74	9.6	9.5	9.6	15.0	14.9	15.0
75 – 79	7.9	7.9	7.9	12.2	12.2	12.3
80 – 84	6.7	6.6	6.7	10.0	10.0	10.1
85 – 89	5.9	5.8	5.9	8.3	8.3	8.4
90 +	5.4	5.3	5.6	7.3	7.1	7.4

B. Parkinson's disease - Male				Matched controls - Male		
Age band	Life expectancy	Lower CI	Upper CI	Life expectancy	Lower CI	Upper CI
20 – 24	44.4	43.0	45.8	57.1	56.5	57.8
25 – 29	40.0	39.0	41.1	52.5	52.2	52.9
30 – 34	36.2	35.5	36.9	47.6	47.3	47.9
35 – 39	31.9	31.4	32.4	42.7	42.5	43.0
40 – 44	28.0	27.7	28.4	38.0	37.8	38.2
45 – 49	24.0	23.7	24.2	33.4	33.2	33.5
50 – 54	20.1	19.9	20.3	28.9	28.7	29.0
55 – 59	16.4	16.3	16.5	24.6	24.5	24.7
60 – 64	13.5	13.4	13.6	20.7	20.6	20.8
65 – 69	10.8	10.8	10.9	17.0	16.9	17.1
70 – 74	8.7	8.6	8.8	13.8	13.7	13.9
75 – 79	7.1	7.0	7.1	11.2	11.1	11.2
80 – 84	5.8	5.8	5.9	9.1	9.0	9.1
85 – 89	5.0	4.9	5.1	7.5	7.4	7.6
90 +	4.4	4.3	4.6	6.5	6.4	6.7

C. Parkinson's disease - Female				Matched controls - Female		
Age band	Life expectancy	Lower CI	Upper CI	Life expectancy	Lower CI	Upper CI
20 – 24	48.1	47.0	49.2	61.4	61.1	61.7
25 – 29	43.1	42.0	44.2	56.4	56.1	56.7
30 – 34	38.8	38.0	39.6	51.4	51.1	51.7
35 – 39	34.6	34.0	35.2	46.5	46.2	46.8
40 – 44	30.4	29.9	30.8	41.6	41.3	41.8
45 – 49	26.5	26.1	26.8	36.9	36.7	37.1
50 – 54	22.6	22.3	22.8	32.4	32.2	32.6
55 – 59	18.9	18.7	19.1	28.0	27.9	28.2
60 – 64	15.7	15.6	15.9	23.9	23.8	24.0
65 – 69	13.1	13.0	13.2	20.0	19.9	20.1
70 – 74	10.8	10.7	10.9	16.6	16.5	16.7
75 – 79	9.0	8.9	9.1	13.5	13.4	13.6
80 – 84	7.8	7.7	7.8	11.1	11.0	11.2
85 – 89	6.9	6.8	7.0	9.2	9.1	9.4
90 +	6.4	6.2	6.6	7.9	7.8	8.1

D. Multiple system atrophy - All / **Matched controls - All**

Age band	Life expectancy	Lower CI	Upper CI	Life expectancy	Lower CI	Upper CI
50 – 54	10.1	9.3	10.8	33.7	32.6	34.8
55 – 59	8.4	7.9	8.9	29.3	28.3	30.4
60 – 64	7.4	6.9	7.8	25.5	24.4	26.5
65 – 69	6.8	6.4	7.3	21.8	20.8	22.9
70 – 74	6.4	5.9	6.8	18.3	17.2	19.4
75 – 79	7.1	6.6	7.6	15.4	14.2	16.6
80 – 84	7.4	6.6	8.2	13.8	12.4	15.3
85 – 89	7.4	6.2	8.6	13.2	11.3	15.2
90 +	8.8	6.5	11.2	13.0	10.3	15.7

E. Multiple system atrophy - Male / **Matched controls - Male**

Age band	Life expectancy	Lower CI	Upper CI	Life expectancy	Lower CI	Upper CI
50 – 54	9.8	8.8	10.7	31.4	30.1	32.7
55 – 59	7.8	7.1	8.5	26.9	25.6	28.2
60 – 64	7.1	6.5	7.7	23.2	22.0	24.5
65 – 69	6.7	6.2	7.3	19.6	18.4	20.9
70 – 74	6.1	5.6	6.6	16.1	14.8	17.5
75 – 79	6.1	5.5	6.6	13.3	11.8	14.8
80 – 84	5.3	4.7	5.9	12.1	10.1	14.0
85 – 89	4.9	3.9	5.9	11.3	8.6	14.0
90 +	6.6	3.9	9.2	12.4	8.1	16.6

F. Multiple system atrophy - Female / **Matched controls – Female**

Age band	Life expectancy	Lower CI	Upper CI	Life expectancy	Lower CI	Upper CI
50 – 54	10.5	9.3	11.7	36.3	34.5	38.0
55 – 59	9.2	8.3	10.0	32.2	30.5	33.8
60 – 64	7.8	7.0	8.5	28.0	26.3	29.7
65 – 69	7.0	6.3	7.6	24.1	22.5	25.8
70 – 74	6.7	5.9	7.4	20.6	18.8	22.3
75 – 79	8.4	7.4	9.5	17.5	15.6	19.4
80 – 84	9.9	8.3	11.5	15.4	13.2	17.5
85 – 89	9.7	7.5	12.0	14.6	11.9	17.4
90 +	10.5	6.9	14.1	13.3	9.8	16.8

G. Progressive supranuclear palsy - All / **Matched controls - All**

Age band	Life expectancy	Lower CI	Upper CI	Life expectancy	Lower CI	Upper CI
50 – 54	8.3	7.5	9.1	30.2	29.5	31.0
55 – 59	6.5	6.0	7.0	26.1	25.5	26.7
60 – 64	5.6	5.3	6.0	22.3	21.8	22.8
65 – 69	5.2	5.0	5.4	18.6	18.2	19.1
70 – 74	4.7	4.5	4.9	15.2	14.7	15.6
75 – 79	4.1	4.0	4.3	12.3	11.8	12.8
80 – 84	3.6	3.5	3.8	9.8	9.2	10.4
85 – 89	3.3	3.1	3.5	7.8	7.0	8.6
90 +	3.4	2.7	4.2	7.0	5.7	8.3

H. Progressive supranuclear palsy - Male / **Matched controls - Male**

Age band	Life expectancy	Lower CI	Upper CI	Life expectancy	Lower CI	Upper CI
50 – 54	7.9	6.9	8.9	29.2	28.3	30.1
55 – 59	5.9	5.3	6.6	25.0	24.4	25.7
60 – 64	5.4	5.1	5.8	21.2	20.6	21.7
65 – 69	5.0	4.7	5.3	17.4	16.9	17.9
70 – 74	4.4	4.2	4.6	14.0	13.5	14.4
75 – 79	3.9	3.8	4.1	11.0	10.6	11.5
80 – 84	3.1	3.0	3.3	8.6	8.0	9.2
85 – 89	2.5	2.5	2.5	6.6	5.8	7.4

90 +	0.0	0.0	0.0	5.2	3.9	6.6

I. Progressive supranuclear palsy - Female / **Matched controls – Female**

Age band	Life expectancy	Lower CI	Upper CI	Life expectancy	Lower CI	Upper CI
50 – 54	9.0	7.7	10.3	31.7	30.4	33.0
55 – 59	7.4	6.6	8.3	27.5	26.4	28.6
60 – 64	6.0	5.4	6.5	23.9	22.9	24.8
65 – 69	5.5	5.1	5.8	20.3	19.5	21.2
70 – 74	5.1	4.8	5.4	16.8	15.9	17.7
75 – 79	4.4	4.2	4.7	13.9	12.9	14.9
80 – 84	4.4	4.0	4.7	11.3	10.1	12.4
85 – 89	4.4	3.9	5.0	9.2	7.8	10.7
90 +	4.1	2.8	5.4	8.8	6.5	11.2

J. Corticobasal syndrome - All / **Matched controls - All**

Age band	Life expectancy	Lower CI	Upper CI	Life expectancy	Lower CI	Upper CI
50 – 54	13.6	11.7	15.5	31.2	29.3	33.1
55 – 59	10.0	8.6	11.3	27.3	25.9	28.6
60 – 64	8.2	7.1	9.2	22.9	21.7	24.1
65 – 69	6.1	5.3	6.9	18.9	17.9	20.0
70 – 74	6.2	5.4	7.0	15.4	14.4	16.4
75 – 79	5.7	4.7	6.6	12.4	11.4	13.4
80 – 84	7.9	6.0	9.7	9.8	8.7	10.9
85 – 89	9.3	6.0	12.6	7.6	6.3	8.9
90 +	10.1	4.6	15.6	5.9	4.0	7.7

K. Corticobasal syndrome - Male / **Matched controls - Male**

Age band	Life expectancy	Lower CI	Upper CI	Life expectancy	Lower CI	Upper CI
50 – 54	13.7	11.6	15.9	29.2	27.4	31.0
55 – 59	9.4	7.6	11.2	24.2	22.4	26.0
60 – 64	7.4	5.8	8.9	20.0	18.4	21.6
65 – 69	5.8	4.7	6.8	16.4	15.1	17.6
70 – 74	5.4	4.4	6.4	13.0	11.8	14.2
75 – 79	4.2	3.1	5.3	10.0	8.9	11.1
80 – 84	8.0	4.4	11.5	7.6	6.4	8.7
85 – 89	11.8	4.7	19.0	5.7	4.4	7.0
90 +	11.1	1.4	20.8	4.1	2.2	5.9

L. Corticobasal syndrome - Female / **Matched controls - Female**

Age band	Life expectancy	Lower CI	Upper CI	Life expectancy	Lower CI	Upper CI
50 – 54	12.9	9.4	16.4	32.5	28.5	36.5
55 – 59	10.4	8.4	12.4	30.2	28.2	32.1
60 – 64	8.9	7.4	10.3	25.5	23.6	27.4
65 – 69	6.3	5.2	7.5	21.2	19.4	23.0
70 – 74	6.7	5.5	7.9	17.4	15.7	19.2
75 – 79	6.7	5.3	8.1	14.3	12.5	16.1
80 – 84	7.8	5.6	9.9	11.5	9.5	13.4
85 – 89	8.2	4.7	11.8	9.2	6.8	11.5
90 +	9.5	2.9	16.1	7.5	4.2	10.8

M. Dementia with Lewy bodies - All / **Matched controls - All**

Age band	Life expectancy	Lower CI	Upper CI	Life expectancy	Lower CI	Upper CI
50 – 54	11.4	10.2	12.7	29.9	29.1	30.7
55 – 59	9.5	8.9	10.2	25.8	25.4	26.2
60 – 64	8.0	7.7	8.3	21.6	21.3	21.9
65 – 69	6.9	6.7	7.1	17.8	17.6	18.0
70 – 74	6.1	6.0	6.3	14.6	14.5	14.8

75 – 79	5.7	5.6	5.8	11.9	11.7	12.0
80 – 84	5.1	5.0	5.2	9.5	9.3	9.6
85 – 89	4.6	4.5	4.8	7.6	7.4	7.8
90 +	4.0	3.8	4.3	6.2	5.8	6.5

N. Dementia with Lewy bodies - Male **Matched controls – Male**

Age band	Life expectancy	Lower CI	Upper CI	Life expectancy	Lower CI	Upper CI
50 – 54	10.3	8.7	11.9	28.2	27.1	29.4
55 – 59	9.0	8.2	9.8	24.6	24.1	25.1
60 – 64	7.6	7.2	8.0	20.4	20.0	20.7
65 – 69	6.5	6.3	6.8	16.6	16.4	16.9
70 – 74	5.6	5.4	5.7	13.6	13.4	13.7
75 – 79	4.9	4.8	5.0	10.9	10.7	11.1
80 – 84	4.3	4.1	4.4	8.5	8.3	8.8
85 – 89	3.9	3.7	4.0	6.8	6.5	7.1
90 +	3.0	2.6	3.3	5.4	5.0	5.9

O. Dementia with Lewy bodies - Female **Matched controls – Female**

Age band	Life expectancy	Lower CI	Upper CI	Life expectancy	Lower CI	Upper CI
50 – 54	13.6	11.6	15.6	32.4	31.6	33.3
55 – 59	10.8	9.5	12.0	27.4	26.6	28.3
60 – 64	8.8	8.1	9.4	23.4	23.0	23.9
65 – 69	7.5	7.2	7.9	19.5	19.1	19.8
70 – 74	7.0	6.8	7.3	15.9	15.6	16.2
75 – 79	6.6	6.5	6.8	12.9	12.7	13.2
80 – 84	5.9	5.7	6.1	10.3	10.1	10.5
85 – 89	5.2	5.0	5.4	8.1	7.8	8.4
90 +	4.7	4.3	5.1	6.6	6.1	7.0

P. Vascular parkinsonism - All **Matched controls - All**

Age band	Life expectancy	Lower CI	Upper CI	Life expectancy	Lower CI	Upper CI
50 – 54	16.3	13.7	18.8	28.8	26.3	31.4
55 – 59	12.2	10.1	14.2	25.5	24.1	26.8
60 – 64	9.7	8.4	10.9	21.5	20.7	22.3
65 – 69	8.4	7.8	9.0	17.6	17.2	18.1
70 – 74	7.0	6.6	7.4	14.0	13.6	14.3
75 – 79	6.0	5.7	6.2	11.0	10.7	11.2
80 – 84	5.0	4.8	5.1	8.5	8.3	8.7
85 – 89	4.2	4.0	4.4	6.6	6.4	6.9
90 +	3.5	3.1	3.8	5.1	4.8	5.5

Q. Vascular parkinsonism - Male **Matched controls - Male**

Age band	Life expectancy	Lower CI	Upper CI	Life expectancy	Lower CI	Upper CI
50 – 54	15.0	11.9	18.1	27.3	24.1	30.4
55 – 59	11.1	8.6	13.6	24.3	22.4	26.1
60 – 64	9.1	7.7	10.5	20.8	19.9	21.6
65 – 69	7.7	7.0	8.5	16.7	16.2	17.3
70 – 74	6.7	6.3	7.2	13.2	12.8	13.6
75 – 79	5.5	5.3	5.8	10.4	10.1	10.7
80 – 84	4.5	4.3	4.7	7.9	7.7	8.2
85 – 89	3.6	3.4	3.7	6.2	5.9	6.4
90 +	3.1	2.7	3.4	4.8	4.4	5.3

R. Vascular parkinsonism - Female **Matched controls – Female**

Age band	Life expectancy	Lower CI	Upper CI	Life expectancy	Lower CI	Upper CI
50 – 54	19.9	16.1	23.7	33.1	31.0	35.2
55 – 59	14.9	11.1	18.7	28.1	26.0	30.2
60 – 64	11.3	8.2	14.4	23.1	21.0	25.2

65 – 69	9.9	8.8	11.1	19.9	19.1	20.7
70 – 74	7.6	6.8	8.3	15.8	15.2	16.4
75 – 79	7.0	6.5	7.5	12.3	11.8	12.8
80 – 84	6.1	5.6	6.5	9.7	9.3	10.2
85 – 89	5.4	5.0	5.9	7.4	6.9	7.9
90 +	4.3	3.5	5.1	5.7	5.0	6.4

S. Drug-induced parkinsonism - All | **Matched controls - All**

Age band	Life expectancy	Lower CI	Upper CI	Life expectancy	Lower CI	Upper CI
20 – 24	44.9	43.3	46.5	59.0	58.0	59.9
25 – 29	39.9	38.3	41.5	54.2	53.4	55.0
30 – 34	35.6	34.2	36.9	49.4	48.7	50.1
35 – 39	31.6	30.5	32.8	44.8	44.2	45.4
40 – 44	27.2	26.2	28.3	39.9	39.3	40.5
45 – 49	23.6	22.8	24.5	35.2	34.7	35.7
50 – 54	20.1	19.4	20.9	30.7	30.2	31.2
55 – 59	17.4	16.8	17.9	26.5	26.1	26.9
60 – 64	14.7	14.3	15.2	22.5	22.1	22.9
65 – 69	12.5	12.1	12.9	18.8	18.5	19.2
70 – 74	10.5	10.2	10.8	15.4	15.1	15.7
75 – 79	9.2	8.9	9.5	12.7	12.4	13.0
80 – 84	7.8	7.5	8.1	10.3	10.0	10.7
85 – 89	6.6	6.2	7.1	8.7	8.2	9.2
90 +	5.6	4.9	6.3	7.3	6.6	8.0

T. Drug-induced parkinsonism - Male | **Matched controls - Male**

Age band	Life expectancy	Lower CI	Upper CI	Life expectancy	Lower CI	Upper CI
20 – 24	43.6	41.7	45.6	56.8	55.5	58.0
25 – 29	38.6	36.7	40.6	52.1	50.9	53.2
30 – 34	34.1	32.3	35.9	47.3	46.4	48.3
35 – 39	30.3	28.8	31.8	42.7	41.8	43.5
40 – 44	26.1	24.8	27.3	37.8	37.0	38.7
45 – 49	22.1	21.0	23.2	33.2	32.4	33.9
50 – 54	18.4	17.5	19.4	28.9	28.2	29.5
55 – 59	15.8	15.1	16.6	24.6	24.0	25.2
60 – 64	13.1	12.5	13.7	20.6	20.1	21.2
65 – 69	11.0	10.5	11.5	17.2	16.7	17.7
70 – 74	9.1	8.7	9.6	13.9	13.4	14.4
75 – 79	7.6	7.3	8.0	11.4	10.9	11.9
80 – 84	6.7	6.3	7.1	9.3	8.8	9.9
85 – 89	5.4	4.9	5.9	8.1	7.3	8.9
90 +	3.6	2.8	4.4	7.2	6.0	8.5

U. Drug-induced parkinsonism - Female | **Matched controls - Female**

Age band	Life expectancy	Lower CI	Upper CI	Life expectancy	Lower CI	Upper CI
20 – 24	46.1	43.1	49.1	61.4	60.3	62.5
25 – 29	41.1	38.1	44.1	56.4	55.3	57.5
30 – 34	37.3	35.3	39.4	51.4	50.3	52.5
35 – 39	33.1	31.3	34.9	46.9	46.1	47.7
40 – 44	28.4	26.8	30.1	41.9	41.1	42.7
45 – 49	25.3	23.9	26.7	37.1	36.3	37.9
50 – 54	22.0	20.9	23.1	32.4	31.7	33.1
55 – 59	18.8	18.0	19.7	28.2	27.6	28.8
60 – 64	16.2	15.5	16.9	24.1	23.6	24.6
65 – 69	13.7	13.1	14.2	20.1	19.7	20.6
70 – 74	11.6	11.1	12.1	16.5	16.1	17.0
75 – 79	10.3	9.9	10.8	13.5	13.1	14.0
80 – 84	8.5	8.0	9.0	10.9	10.5	11.4

85 – 89	7.3	6.7	8.0	9.0	8.4	9.6
90 +	6.7	5.6	7.7	7.3	6.5	8.1

V. Other secondary parkinsonism - All / **Matched controls - All**

Age band	Life expectancy	Lower CI	Upper CI	Life expectancy	Lower CI	Upper CI
20 – 24	40.0	38.1	41.9	58.1	57.2	59.0
25 – 29	36.0	34.3	37.8	53.2	52.3	54.1
30 – 34	32.2	30.7	33.8	48.5	47.6	49.3
35 – 39	28.2	26.8	29.6	43.6	42.7	44.4
40 – 44	25.2	24.1	26.3	38.7	37.9	39.5
45 – 49	21.0	20.0	22.0	34.1	33.3	34.8
50 – 54	17.1	16.2	18.1	29.6	28.9	30.3
55 – 59	14.0	13.2	14.8	25.5	24.9	26.1
60 – 64	11.6	10.9	12.2	21.4	20.9	22.0
65 – 69	9.2	8.6	9.7	17.9	17.4	18.4
70 – 74	7.8	7.4	8.2	14.5	14.1	14.9
75 – 79	6.6	6.2	6.9	11.6	11.2	12.0
80 – 84	5.6	5.2	6.0	9.2	8.8	9.6
85 – 89	5.5	4.9	6.0	7.2	6.7	7.7
90 +	5.4	4.4	6.5	5.9	5.1	6.7

W. Other secondary parkinsonism - Male / **Matched controls - Male**

Age band	Life expectancy	Lower CI	Upper CI	Life expectancy	Lower CI	Upper CI
20 – 24	39.4	37.2	41.7	56.6	55.4	57.7
25 – 29	35.5	33.5	37.6	51.7	50.6	52.8
30 – 34	31.4	29.5	33.2	46.9	45.9	48.0
35 – 39	27.2	25.5	28.9	41.9	40.9	43.0
40 – 44	24.3	23.1	25.6	37.1	36.1	38.1
45 – 49	19.7	18.5	21.0	32.3	31.4	33.3
50 – 54	16.1	15.0	17.2	27.9	27.0	28.8
55 – 59	12.7	11.8	13.6	24.0	23.2	24.7
60 – 64	10.4	9.7	11.2	19.9	19.2	20.6
65 – 69	8.2	7.6	8.9	16.5	15.9	17.1
70 – 74	7.0	6.5	7.5	13.2	12.7	13.7
75 – 79	6.0	5.6	6.4	10.5	10.0	11.0
80 – 84	5.0	4.6	5.4	8.5	8.0	9.0
85 – 89	5.2	4.5	5.9	6.6	5.9	7.2
90 +	5.2	3.7	6.7	5.3	4.2	6.4

X. Other secondary parkinsonism - Female / **Matched controls - Female**

Age band	Life expectancy	Lower CI	Upper CI	Life expectancy	Lower CI	Upper CI
20 – 24	41.2	37.7	44.6	60.4	58.8	62.0
25 – 29	37.0	33.9	40.1	55.4	53.8	57.0
30 – 34	33.8	31.0	36.5	50.7	49.2	52.2
35 – 39	30.0	27.6	32.5	46.0	44.6	47.4
40 – 44	26.8	24.8	28.7	41.3	40.0	42.5
45 – 49	23.3	21.6	25.0	36.7	35.5	37.9
50 – 54	19.0	17.3	20.6	32.2	31.1	33.3
55 – 59	16.2	14.9	17.5	27.7	26.7	28.8
60 – 64	13.5	12.4	14.6	23.7	22.8	24.6
65 – 69	10.7	9.8	11.7	20.0	19.2	20.8
70 – 74	9.0	8.3	9.6	16.2	15.6	16.9
75 – 79	7.3	6.8	7.9	13.0	12.4	13.6
80 – 84	6.3	5.7	6.9	10.0	9.3	10.6
85 – 89	5.8	5.0	6.5	7.8	7.1	8.6
90 +	5.6	4.2	7.0	6.3	5.2	7.4

All life expectancy values are at the start of the age interval.

Supplementary Table 8: Extended Cox model of survival in Parkinson's cases versus matched controls

Years after diagnosis	Covariate	Adjusted hazard ratio	95% confidence interval	P value
	Male sex	1.3	1.3, 1.3	<0.0001
0 - 7 years				
	Parkinson’s disease case	1.9	1.9, 1.9	<0.0001
	65 - 74 years old at diagnosis	3.3	3.2, 3.4	<0.0001
	75 - 84 years old at diagnosis	7.3	7.1, 7.6	<0.0001
	≥ 85 years old at diagnosis	12.1	11.7, 12.6	<0.0001
7 - 14 years				
	Parkinson’s disease case	2.2	2.2, 2.3	<0.0001
	65 - 74 years old at diagnosis	2.9	2.8, 3.0	<0.0001
	75 - 84 years old at diagnosis	3.4	3.3, 3.6	<0.0001
	≥ 85 years old at diagnosis	1.4	1.3, 1.5	<0.0001
14 - 21 years				
	Parkinson’s disease case	2.2	2.1, 2.3	<0.0001
	65 - 74 years old at diagnosis	1.8	1.6, 1.9	<0.0001
	75 - 84 years old at diagnosis	NA	NA	NA
	≥ 85 years old at diagnosis	NA	NA	NA

PD controls are the reference group for diagnosis, and cases under 65 years are the reference group for age at diagnosis.
Analysis by multivariable model; hazard ratios are adjusted for all covariates.
NA=not applicable; data for 14-21 years for people aged 75+ at diagnosis are not applicable as there are too few survivors.

Supplementary Table 9: Demographic and clinical features of incident non-degenerative parkinsonism cases in 2023

	Vascular parkinsonism	Drug-induced parkinsonism	Other secondary parkinsonism	All cases (including Table 1 cases)
Number of cases (% of all cases)	342 (4.5%)	139 (1.8%)	64 (0.8%)	7 632 (100.0%)
Crude incidence (95% CI)	2.0 (1.8, 2.2)	0.81 (0.68, 0.95)	0.37 (0.29, 0.47)	44.3 (43.3, 45.3)
Standardized incidence (95% CI)	2.0 (1.8, 2.2)	0.83 (0.69, 0.97)	0.38 (0.29, 0.47)	45.3 (44.3, 46.3)
Age at diagnosis, mean (SD)	80.4 (7.2)	70.7 (11.6)	74.9 (10.7)	75.3 (9.2)
**Sex**				
Male, n (%)	196 (71.3%)	59 (48.4%)	33 (57.9%)	4 181 (63.7%)
Female, n (%)	79 (28.7%)	63 (51.6%)	24 (42.1%)	2 380 (36.3%)
**Smoking status**				
Current smoker, n (%)	32 (11.6%)	28 (23.0%)	11 (19.3%)	650 (9.9%)
Former smoker, n (%)	188 (68.4%)	67 (54.9%)	34 (59.6%)	4 171 (63.5%)
Never smoked, n (%)	54 (19.6%)	24 (19.3%)	11 (19.3%)	1 703 (26.0%)
Missing, n (%)	1 (0.4%)	3 (2.5%)	1 (1.8%)	37 (0.6%)
**BMI, kg / m2**				
Mean (SD)	27.0 (7.1)	28.6 (6.3)	27.5 (6.6)	26.7 (5.2)
**Dopaminergic medication**				
Current, n (%)	116 (42.2%)	12 (9.8%)	26 (45.6%)	4 674 (71.2%)
Previous, n (%)	9 (3.3%)	4 (3.3%)	2 (3.5%)	68 (1.0%)
Never, n (%)	150 (54.5%)	106 (86.9%)	29 (50.9%)	1 819 (27.7%)
**Current drug class**				
L-dopa, n (%)	114 (41.5%)	12 (9.8%)	26 (45.6%)	4 534 (69.1%)
Dopamine agonist, n (%)	8 (2.9%)	0 (0.0%)	3 (5.3%)	313 (4.7%)
MAOB inhibitor, n (%)	0 (0.0%)	0 (0.0%)	0 (0.0%)	194 (2.9%)
COMT inhibitor, n (%)	0 (0.0%)	0 (0.0%)	1 (1.8%)	82 (1.2%)
Amantadine, n (%)	1 (0.4%)	0 (0.0%)	2 (3.5%)	46 (0.7%)

The percentage of cases is expressed out of total numbers of both degenerative and non-degenerative parkinsonisms. Incidence rates are expressed per 100 000 people for the whole population; standardized rates are to European standard 2013. Percentages may not sum because of rounding.
Numbers of cases and incidence rates include uplift based on hospital admissions; other variables do not include uplift. Results are expressed as mean (SD) unless otherwise stated.
BMI=body mass index; CI=confidence interval; COMT=catechol-O-methyltransferase; MAOB=monoamine oxidase type B; SD=standard deviation; NA=not applicable.

Supplementary Table 10: Demographic and clinical features of prevalent non-degenerative parkinsonism cases in 2023

	Vascular parkinsonism	Drug-induced parkinsonism	Other secondary parkinsonism	All cases (including Table 2 cases)
Number of cases	855 (1.8%)	1 299 (2.7%)	925 (1.9%)	47 925 (100.0%)
Crude prevalence (95% CI)	4.7 (4.4, 5.1)	7.2 (6.8, 7.6)	5.1 (4.8, 5.5)	265 (262, 267)
Standardized prevalence (95% CI)	4.9 (4.6, 5.2)	7.6 (7.2, 8.0)	5.3 (5.0, 5.7)	279 (277, 282)
Age at diagnosis, mean (SD)	78.0 (8.2)	59.8 (17.0)	55.5 (19.7)	69.2 (12.3)
**Sex**				
Male n (%)	560 (66.5%)	563 (46.8%)	421 (57.7%)	26 223 (58.3%)
Female n (%)	282 (33.5%)	639 (53.2%)	309 (42.3%)	20 092 (41.7%)
**Smoking status**				
Current smoker, n (%)	106 (12.6%)	242 (20.1%)	145 (19.9%)	4 772 (10.6%)
Former smoker, n (%)	540 (64.1%)	698 (58.1%)	406 (55.6%)	26 814 (59.6%)
Never smoked, n (%)	179 (21.3%)	243 (20.2%)	163 (22.3%)	12 872 (28.6%)
Missing, n (%)	17 (2.0%)	19 (1.6%)	16 (2.2%)	557 (1.2%)
**BMI, kg / m2**				
Mean (SD)	26.4 (5.5)	28.4 (6.7)	28.1 (6.7)	26.5 (5.8)
**Dopaminergic medication**				
Current, n (%)	294 (34.9%)	65 (5.4%)	107 (14.7%)	33 944 (75.4%)
Previous, n (%)	137 (16.3%)	67 (5.6%)	75 (10.3%)	2631 (5.8%)
Never, n (%)	411 (48.8%)	1070 (89.0%)	548 (75.1%)	8435 (18.7%)
**Current drug class**				
L-dopa, n (%)	284 (33.2%)	52 (4.0%)	97 (10.5%)	32 892 (68.6%)
Dopamine agonist, n (%)	27 (3.2%)	10 (0.8%)	16 (1.7%)	7 835 (16.3%)
MAOB inhibitor, n (%)	7 (0.8%)	2 (0.2%)	15 (1.6%)	5 554 (11.6%)
COMT inhibitor, n (%)	2 (0.2%)	1 (0.1%)	9 (1.0%)	4 755 (9.9%)
Amantadine n (%)	2 (0.2%)	2 (0.2%)	4 (0.4%)	1 936 (4.0%)
Duration of therapy in years, mean (SD)	2.0 (2.4)	2.7 (4.2)	3.2 (3.4)	5.2 (5.0)

Percentage of cases is expressed out of both degenerative and non-degenerative parkinsonisms combined. Prevalence rates are expressed per 100 000 people for the whole population; standardized rates are to European standard 2013.

Percentages may not sum because of rounding.
Numbers of cases and incidence rates include uplift based on hospital admissions; other variables do not include uplift.

Results are expressed as mean (SD) unless otherwise stated.
BMI: body mass index; CI: confidence interval; COMT: catechol-O-methyltransferase; MAOB: monoamine oxidase type B; SD: standard deviation; NA: not applicable.

Supplementary Table 11A: Incidence rates of parkinsonism in the United Kingdom in 2023

	All cases		Males		Females	
	**Crude incidence (95% CI)**	**European standardized incidence (95% CI)**	**Crude incidence (95% CI)**	**European standardized incidence (95% CI)**	**Crude incidence (95% CI)**	**European standardized incidence (95% CI)**
Parkinson's disease	33.8 (33.0, 34.7)	34.7 (33.8, 35.6)	43.4 (42.1, 44.8)	48.7 (47.2, 50.2)	23.9 (22.9, 25.0)	22.7 (21.7, 23.7)
Multiple System Atrophy	0.74 (0.61, 0.88)	0.76 (0.63, 0.90)	0.80 (0.62, 1.01)	0.89 (0.68, 1.10)	0.67 (0.51, 0.87)	0.67 (0.49, 0.84)
Progressive Supranuclear Palsy	1.6 (1.5, 1.8)	1.7 (1.5, 1.9)	1.9 (1.6, 2.2)	2.1 (1.8, 2.4)	1.4 (1.2, 1.7)	1.3 (1.1, 1.6)
Corticobasal Syndrome	0.34 (0.26, 0.44)	0.35 (0.26, 0.44)	0.23 (0.14, 0.35)	0.24 (0.14, 0.35)	0.46 (0.33, 0.63)	0.44 (0.30, 0.57)
Dementia with Lewy Bodies	4.6 (4.3, 4.9)	4.6 (4.3, 4.9)	5.4 (4.9, 5.9)	6.1 (5.6, 6.7)	3.7 (3.3, 4.2)	3.4 (3.0, 3.7)
Vascular parkinsonism	2.0 (1.8, 2.2)	2.0 (1.8, 2.2)	2.8 (2.5, 3.2)	3.2 (2.8, 3.7)	1.1 (0.9, 1.4)	1.0 (0.8, 1.2)
Drug-induced parkinsonism	0.81 (0.68, 0.95)	0.83 (0.69, 0.97)	0.77 (0.59, 0.97)	0.83 (0.63, 1.03)	0.85 (0.66, 1.07)	0.82 (0.63, 1.01)
Other secondary parkinsonism	0.37 (0.29, 0.47)	0.38 (0.29, 0.47)	0.43 (0.31, 0.60)	0.49 (0.33, 0.65)	0.31 (0.20, 0.45)	0.29 (0.18, 0.40)
All parkinsonism	44.3 (43.3, 45.3)	45.3 (44.3, 46.3)	55.7 (54.2, 57.3)	62.6 (60.8, 64.3)	32.5 (31.3, 33.7)	30.6 (29.5, 31.8)

Supplementary Table 11B: Prevalence rates of parkinsonism in the United Kingdom in 2023

	All cases		Males		Females	
	**Crude prevalence (95% CI)**	**European standardized prevalence (95% CI)**	**Crude prevalence (95% CI)**	**European standardized prevalence (95% CI)**	**Crude prevalence (95% CI)**	**European standardized prevalence (95% CI)**
Parkinson's disease	227 (225, 230)	240 (238, 242)	264 (261, 267)	307 (303, 311)	190 (187, 193)	184 (181, 186)
Multiple System Atrophy	2.5 (2.2, 2.7)	2.6 (2.4, 2.8)	2.5 (2.1, 2.8)	2.8 (2.4, 3.1)	2.5 (2.1, 2.8)	2.5 (2.1, 2.8)
Progressive Supranuclear Palsy	3.6 (3.3, 3.9)	3.8 (3.5, 4.1)	3.7 (3.3, 4.1)	4.3 (3.8, 4.7)	3.5 (3.1, 3.9)	3.4 (3.0, 3.8)
Corticobasal Syndrome	1.1 (1.0, 1.3)	1.2 (1.0, 1.4)	0.9 (0.8, 1.2)	1.1 (0.8, 1.3)	1.3 (1.1, 1.6)	1.3 (1.1, 1.6)
Dementia with Lewy Bodies	13.3 (12.8, 13.8)	13.9 (13.3, 14.4)	14.1 (13.4, 14.9)	16.8 (15.9, 17.7)	12.5 (11.7, 13.2)	11.4 (10.7, 12.1)
Vascular parkinsonism	4.7 (4.4, 5.1)	4.9 (4.6, 5.2)	6.2 (5.7, 6.7)	7.4 (6.8, 8.1)	3.2 (2.9, 3.6)	2.9 (2.6, 3.3)
Drug-induced parkinsonism	7.2 (6.8, 7.6)	7.6 (7.2, 8.0)	6.9 (6.3, 7.4)	7.7 (7.1, 8.3)	7.5 (7.0, 8.1)	7.4 (6.9, 8.0)
Other secondary parkinsonism	5.1 (4.8, 5.5)	5.3 (5.0, 5.7)	5.9 (5.4, 6.4)	6.4 (5.8, 6.9)	4.3 (3.9, 4.7)	4.3 (3.9, 4.7)
All parkinsonism	265 (262, 267)	279 (277, 282)	304 (300, 307)	353 (349, 358)	225 (222, 228)	217 (214, 220)
England	227 (225, 229)	245 (243, 248)	262 (258, 266)	313 (308, 317)	191 (188, 194)	188 (185, 192)
Northern Ireland	229 (212, 247)	239 (221, 257)	280 (254, 307)	317 (287, 347)	178 (158, 200)	170 (150, 190)
Scotland	212 (204, 220)	204 (197, 211)	252 (240, 263)	263 (251, 274)	172 (163, 182)	153 (145, 162)
Wales	256 (246, 266)	225 (217, 234)	309 (294, 325)	292 (277, 307)	203 (191, 216)	168 (158, 179)

Supplementary Table 12: Male to female incidence and prevalence rate ratios for the eight parkinsonian disorders

Type of parkinsonism	Incidence rate ratios (IRR)	P values	Prevalence rate ratios (PRR)	P values
Parkinson's disease	1.7 (1.4, 2.0)	<0.0001	1.4 (1.2, 1.7)	<0.0001
Multiple System Atrophy	1.3 (1.2, 1.4)	<0.0001	1.3 (1.1, 1.5)	0.011
Progressive Supranuclear Palsy	1.7 (1.4, 2.1)	<0.0001	1.4 (1.2, 1.7)	0.00016
Corticobasal Syndrome	0.87 (0.75, 1.01)	0.075	0.8 (0.8, 0.9)	0.00027
Dementia with Lewy Bodies	1.4 (1.2, 1.7)	0.0003	1.4 (1.1, 1.6)	0.0022
Vascular parkinsonism	2.7 (2.1, 3.3)	<0.0001	1.9 (1.5, 2.3)	<0.0001
Drug-induced parkinsonism	1.0 (0.9, 1.2)	0.75	0.92 (0.79, 1.1)	0.31
Other secondary parkinsonism	1.9 (1.6, 2.2)	<0.0001	1.6 (1.4, 1.9)	<0.0001

Incidence and prevalence rate ratios are based on person years in crude case counts from 2003 to 2023, adjusted for age and year.

Supplementary Table 13: Sociodemographic factors in cases of Parkinson's disease in 2023.

	Number of people, denominator (%)	Crude incidence (CI)	European standardized incidence (CI)	Crude prevalence (CI)	European standardized prevalence (CI)
Ethnicity					
African or Caribbean	611 237 (3.9%)	9 (6, 11)	19 (14, 24)	83 (76, 91)	184 (168, 200)
Asian	1 911 637 (12.1%)	12 (11,14)	29 (26, 33)	96 (92, 101)	247 (236, 258)
Mixed or Other	786 477 (5.0%)	5 (4, 7)	19 (13, 25)	47 (42, 52)	188 (169, 208)
White	12 457 770 (79.0%)	36 (35, 37)	31 (30, 32)	255 (252, 258)	232 (229, 234)
Missing	2 325 363 (12.9%)	44 (41, 47)	45 (42, 47)	173 (167, 178)	187 (181, 193)
Socioeconomic status quintile					
5 (least deprived)	2 005 758 (20.0%)	49 (46, 52)	37 (34, 39)	324 (316, 332)	254 (248, 260)
4	1 990 008 (19.8%)	42 (39, 45)	33 (31, 35)	294 (286, 302)	245 (239, 251)
3	1 938 142 (19.3%)	38 (35, 40)	34 (31, 36)	251 (244, 258)	238 (231, 244)
2	2 109 004 (21.0%)	28 (26, 30)	30 (28, 33)	205 (198, 211)	236 (229, 243)
1 (most deprived)	2 006 325 (20.0%)	20 (18, 22)	26 (23, 28)	168 (172, 173)	224 (217, 232)
Missing	8 043 303 (44.5%)	28 (27, 29)	32 (30, 33)	183 (181, 187)	211 (208, 215)
Urban versus rural living					
Urban	16 109 795 (89.0%)	30 (29, 31)	32 (31, 36)	198 (195, 200)	231 (228, 233)
Rural	1 982 745 (11.0%)	47 (45, 41)	34 (32, 36)	313 (305, 321)	237 (231, 243)
Missing n (%)	0 (0%)	0	0	0	0

Incidence is calculated by person-years; prevalence by persons.

Percentage values are out of the total with known values.

Data are not included for uplifted cases as they are not available.

All rates are per 100 000 of the total population.
