# Supplementary figures and images for: Comparative Analysis of the Incidence, Prevalence, and Survival of 8 Types of Parkinsonism in a Population‐Based Study with 367 Million Person Years of Observation over 21 Years
Source: Mov Disord Clin Pract. 2025 Oct 22;13(4):933–48. doi: 10.1002/mdc3.70368 (PMC13071333; doi:10.1002/mdc3.70368)

**Incident Parkinson's disease**

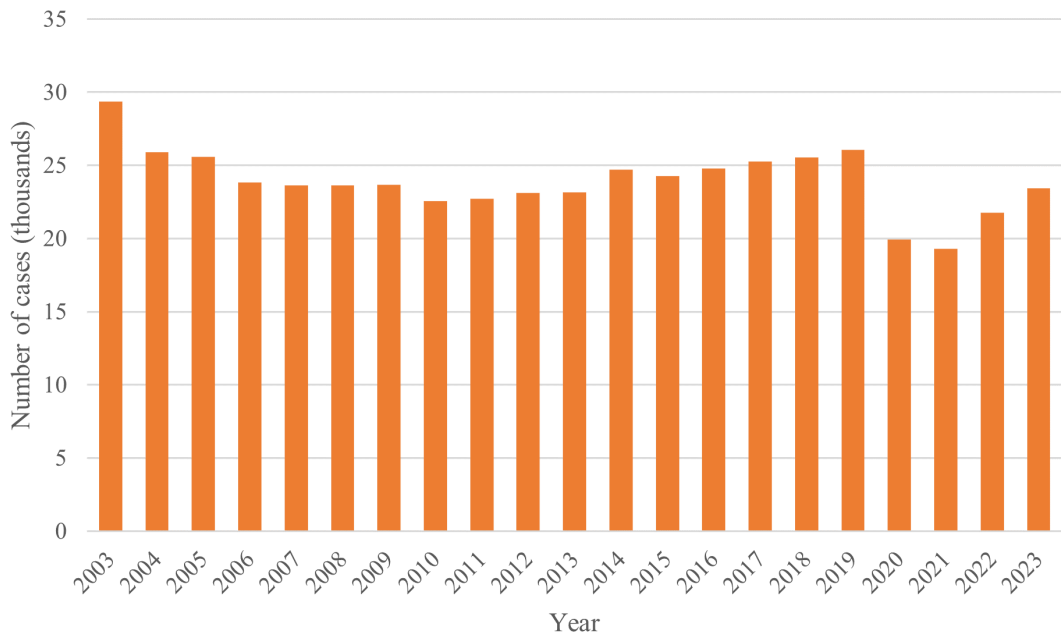

**Prevalent Parkinson's disease**

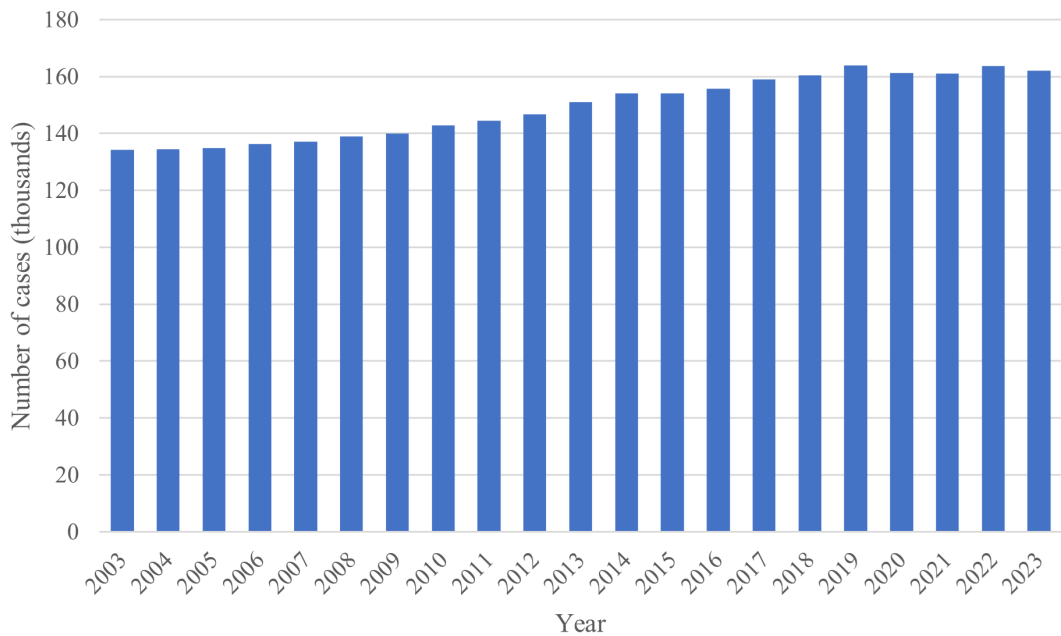

Supplement: Supplementary file 4 — Figure S1. Estimated numbers of incident and prevalent Parkinson's in the UK from 2003 to 2023 inclusive. Absolute case numbers with incident Parkinson's disease initially declined before increasing between 2003 and 2019. Case numbers declined in 2020, due to Covid‐19, but are recovering. Prevalent case numbers steadily increased until 2019, after which there was a slight fall. [file MDC3-13-933-s003.pdf]

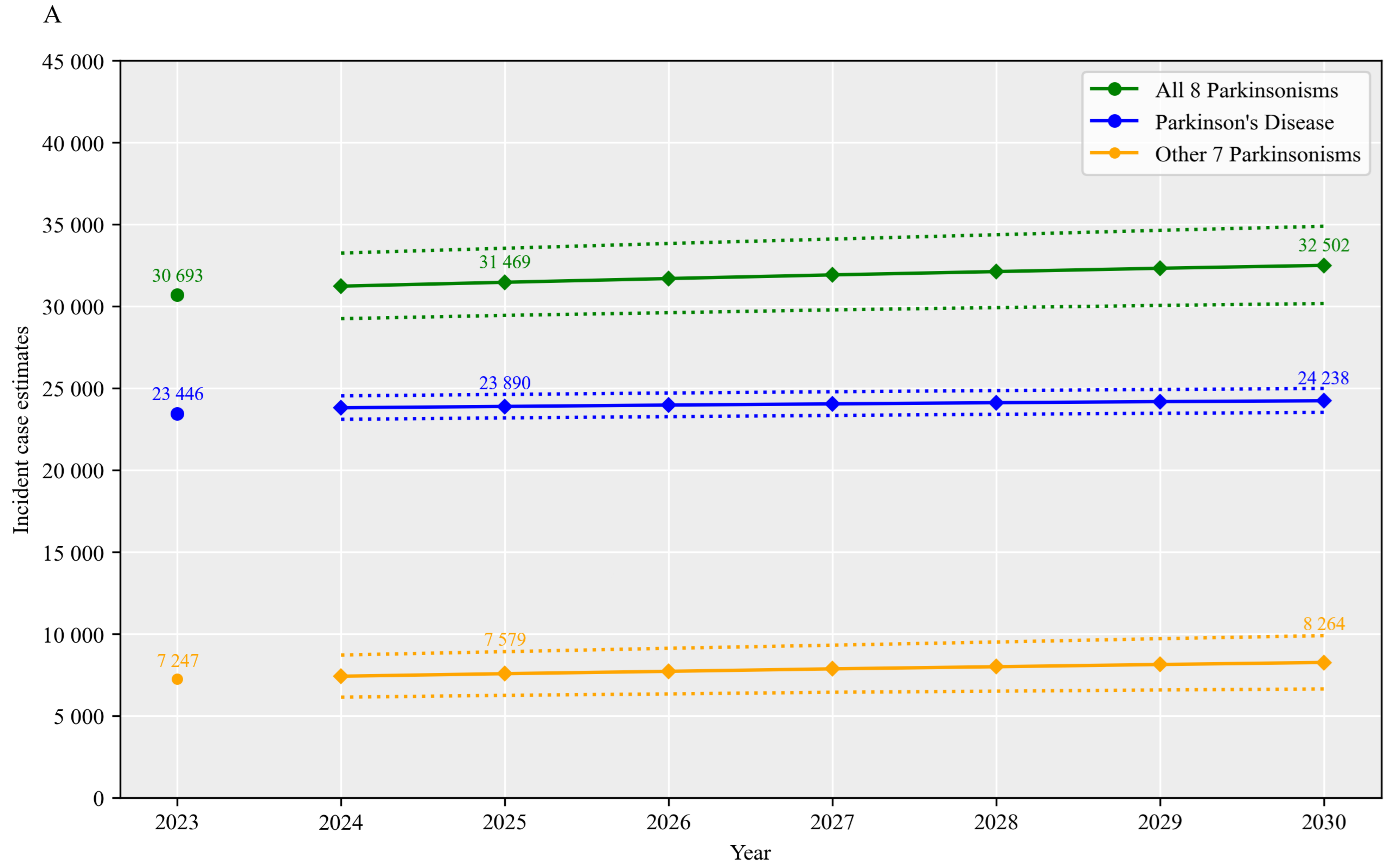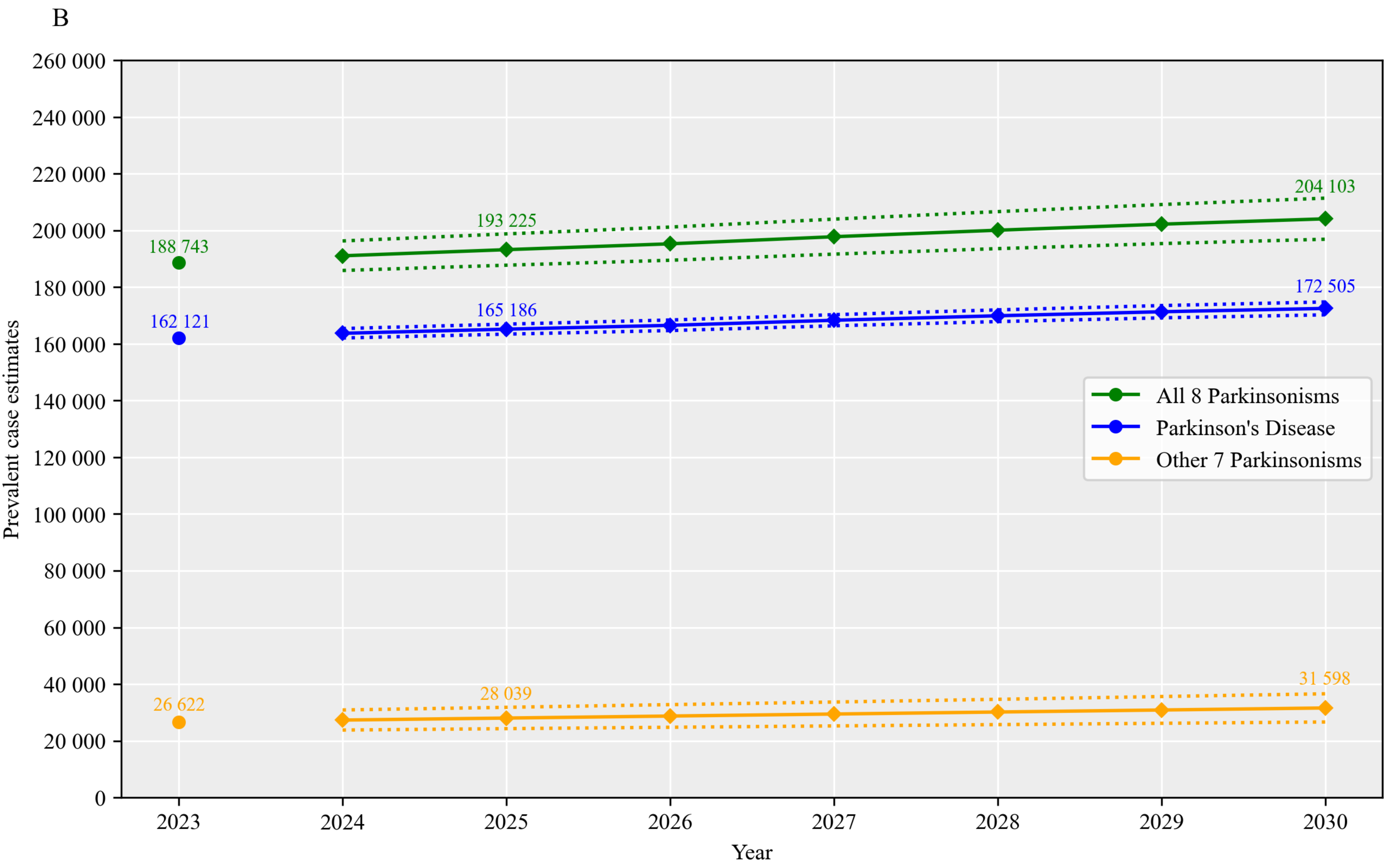

Supplement: Supplementary file 5 — Figure S2. Future projections of UK cases of incident and prevalent Parkinson's disease and other forms of parkinsonism. Future projections of total UK case numbers, based upon pre‐Covid temporal trends and predicted population numbers. A proportionate increase in older people is expected to increase case numbers. Data are mean and 95% confidence intervals. [file MDC3-13-933-s007.pdf]

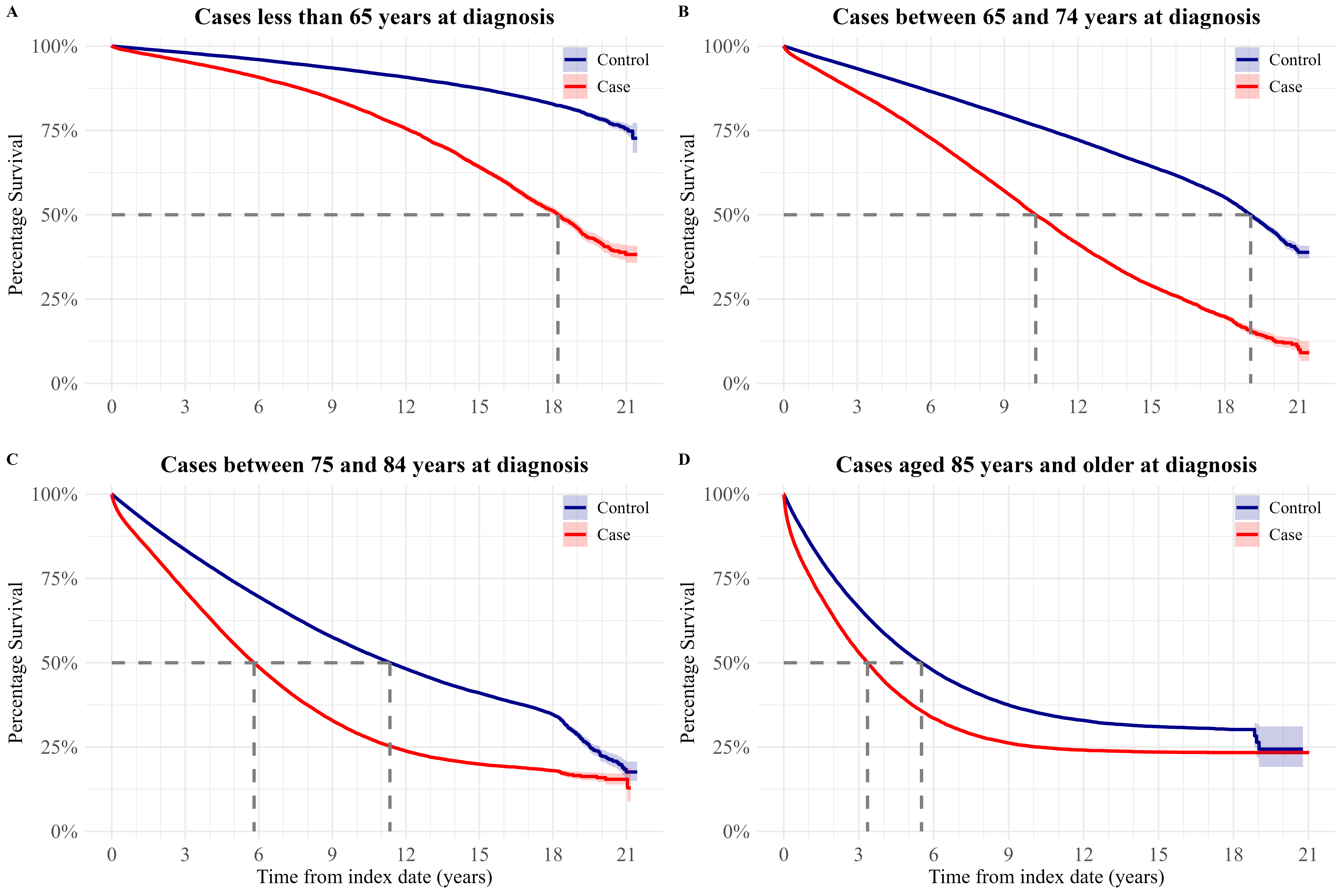

Supplement: Supplementary file 6 — Figure S3. Age‐stratified Kaplan–Meier plots for Parkinson's disease compared with controls. The difference in median survival between Parkinson's disease cases and matched controls decreased progressively with increasing age. Accordingly, the greatest impact of Parkinson's on survival was in the youngest cases. [file MDC3-13-933-s006.png]
