# Supplementary material for: Comparative Analysis of the Incidence, Prevalence, and Survival of 8 Types of Parkinsonism in a Population‐Based Study with 367 Million Person Years of Observation over 21 Years
Source: Mov Disord Clin Pract. 2025 Oct 22;13(4):933–48. doi: 10.1002/mdc3.70368 (PMC13071333; doi:10.1002/mdc3.70368)
Supplement: Supplementary file 7 — Figure S4. Crude incidence of Parkinson's disease and other types of parkinsonism by age and sex. For all parkinsonisms, crude incidence increased with age but then began to decline. Crude incidence was higher in men than women, except for corticobasal syndrome and drug‐induced parkinsonism. [file MDC3-13-933-s002.pdf]

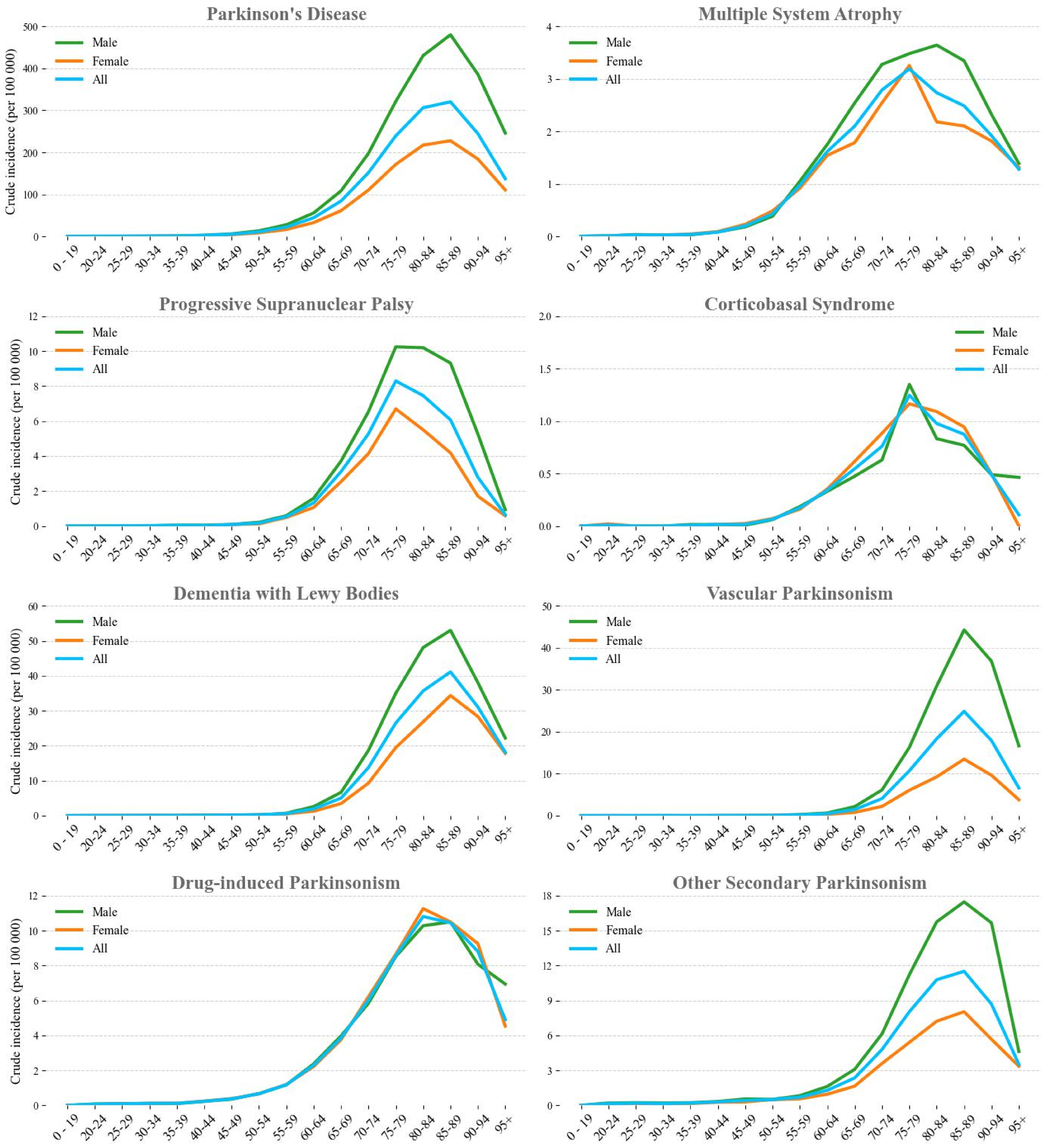
Parkinson's Disease
Multiple System Atrophy
Progressive Supranuclear Palsy
Corticobasal Syndrome
Dementia with Lewy Bodies
Vascular Parkinsonism
Drug-induced Parkinsonism
Other Secondary Parkinsonism
Male
Female
All
Crude incidence (per 100 000)
0 - 19
20-24
25-29
30-34
35-39
40-44
45-49
50-54
55-59
60-64
65-69
70-74
75-79
80-84
85-89
90-94
95+
