# Supplementary material for: Comparative Analysis of the Incidence, Prevalence, and Survival of 8 Types of Parkinsonism in a Population‐Based Study with 367 Million Person Years of Observation over 21 Years
Source: Mov Disord Clin Pract. 2025 Oct 22;13(4):933–48. doi: 10.1002/mdc3.70368 (PMC13071333; doi:10.1002/mdc3.70368)
Supplement: Supplementary file 8 — Figure S5. Crude prevalence of Parkinson's disease and other types of parkinsonism in 2023 by age and sex. Crude prevalence of parkinsonisms increased to a peak, then declined (numbers were very small for some diagnoses in the oldest age band). Corticobasal syndrome was more common in women, while the remaining parkinsonisms, except drug‐induced, were more common in men. [file MDC3-13-933-s005.pdf]

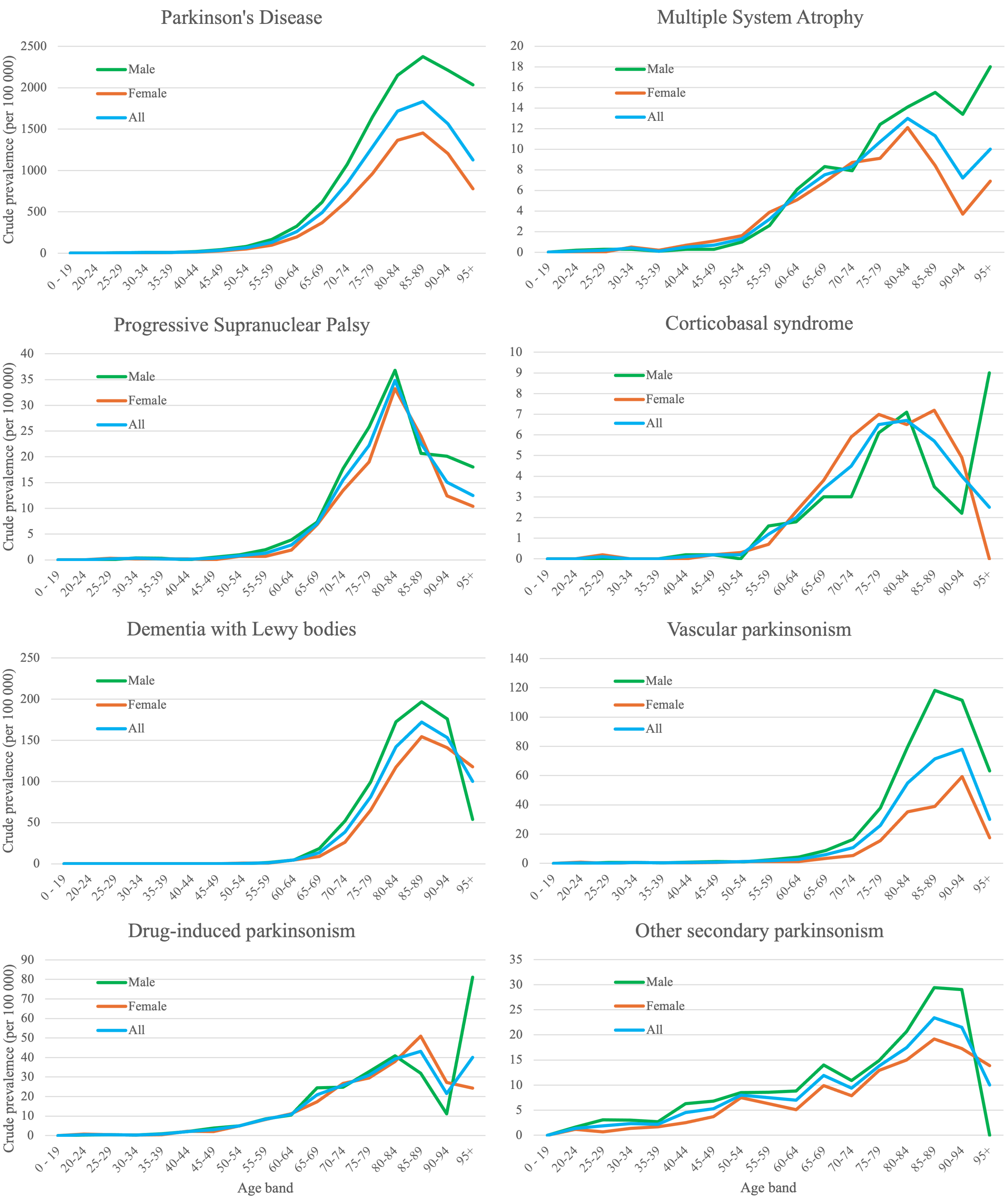
Parkinson's Disease
Multiple System Atrophy
Progressive Supranuclear Palsy
Corticobasal syndrome
Dementia with Lewy bodies
Vascular parkinsonism
Drug-induced parkinsonism
Other secondary parkinsonism
Male
Female
All
Crude prevalemce (per 100 000)
Age band
0 - 19
20-24
25-29
30-34
35-39
40-44
45-49
50-54
55-59
60-64
65-69
70-74
75-79
80-84
85-89
90-94
95+
